# Evolution of the shut-off steps of vertebrate phototransduction

**DOI:** 10.1098/rsob.170232

**Published:** 2018-01-10

**Authors:** Trevor D. Lamb, Hardip R. Patel, Aaron Chuah, David M. Hunt

**Affiliations:** 1 Eccles Institute of Neuroscience, John Curtin School of Medical Research, The Australian National University, Australian Capital Territory 2600, Australia; 2 National Centre for Indigenous Genomics, John Curtin School of Medical Research, The Australian National University, Australian Capital Territory 2600, Australia; 3 Genome Discovery Unit, John Curtin School of Medical Research, The Australian National University, Australian Capital Territory 2600, Australia; 4 The Lions Eye Institute, The University of Western Australia, Western Australia 6009, Australia; 5 School of Biological Sciences, The University of Western Australia, Western Australia 6009, Australia

**Keywords:** evolution, phototransduction, shut-off, G-protein receptor kinase, arrestin, regulator of G-protein signalling

## Abstract

Different isoforms of the genes involved in phototransduction are expressed in vertebrate rod and cone photoreceptors, providing a unique example of parallel evolution via gene duplication. In this study, we determine the molecular phylogeny of the proteins underlying the shut-off steps of phototransduction in the agnathan and jawed vertebrate lineages. For the G-protein receptor kinases (GRKs), the GRK1 and GRK7 divisions arose prior to the divergence of tunicates, with further expansion during the two rounds of whole-genome duplication (2R); subsequently, jawed and agnathan vertebrates retained different subsets of three isoforms of GRK. For the arrestins, gene expansion occurred during 2R. Importantly, both for GRKs and arrestins, the respective rod isoforms did not emerge until the second round of 2R, just prior to the separation of jawed and agnathan vertebrates. For the triplet of proteins mediating shut-off of the G-protein transducin, RGS9 diverged from RGS11, probably at the second round of 2R, whereas Gβ5 and R9AP appear not to have undergone 2R expansion. Overall, our analysis provides a description of the duplications and losses of phototransduction shut-off genes that occurred during the transition from a chordate with only cone-like photoreceptors to an ancestral vertebrate with both cone- and rod-like photoreceptors.

## 1. Introduction

The rod and cone photoreceptors of the vertebrate duplex retina used, respectively, for night and day vision employ distinct protein isoforms for many of the components of the transduction cascade. These cells therefore represent a unique evolutionary system, where the same process (detection of light) uses a distinct set of genes in different classes of cells. For the four proteins underlying activation of the response (Gα, PDE6, CNGα and CNGβ), it is clear that the distinct rod and cone isoforms arose during the two rounds of whole-genome duplication (2R) [1] that occurred during early vertebrate evolution [2–8].

The recovery steps in phototransduction are crucial to the organism's ability to detect rapid changes in visual stimuli, but less is known about the evolution of the rod and cone isoforms of these components. The rapid shut-off of activated visual pigment (rhodopsin or its cone equivalent) is a two-step process that first involves phosphorylation by a G-protein receptor kinase (GRK), and then capping of the phosphorylated pigment by a visual arrestin. Both of these proteins have distinct rod and cone isoforms, GRK1 and GRK7, and Arr-S and Arr-C. The subsequent rapid shut-off of activated G-protein (and thereby of activated PDE6) is accomplished by a triplet of proteins, RGS9, Gβ5 and R9AP, that are common to both rods and cones.

In this paper, we investigate the evolution of these shut-off proteins in vertebrate organisms. For each protein family, we extract cDNA transcripts from the database we previously constructed for three agnathan species and five species of basal fish [8]. We then curate a set of protein sequences for each component of interest, by including sequences from a broad range of taxa in public databases (primarily NCBI). Next, we align these selected sequences and conduct phylogenetic inference, using two recently available tools: SATé (simultaneous alignment and tree estimation) [9] and IQ-Tree [10]. Where appropriate, we apply constraints on the tree inference procedure, in order to test the likelihood that expansion of the family resulted from 2R whole-genome duplication. In addition, we examine synteny in two cases where this is feasible. Together, these approaches permit us to determine the likely patterns of gene duplication and loss in the each of the protein families involved in termination of the vertebrate photoresponse. Thereafter, we are able to compare the molecular features of the phototransduction proteins used in agnathan photoreceptors with those used in the photoreceptors of jawed vertebrates.

## 2. Results

### 2.1. G-protein receptor kinases

#### 2.1.1. Background

GRKs are members of the protein kinase A, G and C (AGC) families. They phosphorylate specific residues of activated G-protein-coupled receptors (GPCRs), typically in the carboxy-terminal region of the GPCR. Mammals possess seven GRKs that fall into three families: (i) the ‘visual’ GRKs (GRK1 and GRK7) that are considered here; (ii) a set of three nearest relatives (GRK4, GRK5 and GRK6); and (iii) a pair of more distant ‘β-adrenergic GRKs’ (GRK2 and GRK3). The molecular structure of GRKs has been reviewed recently [11]. An investigation of the origin of GRKs [12] indicated that the divergence of the β-adrenergic GRKs occurred prior to the emergence of metazoa, whereas the divergence of the visual and GRK4/5/6 families occurred around the time that vertebrates evolved.

In photoreceptors, the function of GRKs is to phosphorylate photoactivated visual pigment (rod or cone opsin) and thereby permit the binding of arrestin, which quenches the activity of the pigment. Although the existence of the two main classes of photoreceptor-specific GRKs (GRK1 and GRK7) has long been known, it is barely a decade since the existence of two distinct isoforms of GRK1, named GRK1A and GRK1B, was discovered [13]. These isoforms were shown to have diverged at an early stage in the evolution of vertebrates [13], and subsequently, it has become clear that both isoforms are present in most vertebrate taxa. One exception is that mammals have lost GRK1B, so that any reference to GRK1 in a mammal signifies the GRK1A group.

For jawed vertebrate photoreceptor classes, the pattern of expression of GRK isoforms has been summarized in a number of species by Osawa & Weiss [14] in their table 1. Many examples of co-expression of GRK1 and GRK7 have been reported, and the observed distribution of isoforms does not have an obvious pattern. Nevertheless, we suggest that the following two rules apply to all jawed vertebrate species that have been studied: (i) if the GRK1A isoform exists in a species, then it is expressed in the rod photoreceptors; (ii) if the GRK7 isoform exists, then it is expressed in the cone photoreceptors. Subsequently, we will present evidence supporting the notion that the third isoform, GRK1B, is normally expressed in cones. Based on these proposed rules, we adopt the shorthand that GRK1A is a ‘scotopic isoform’ (which we indicate with blue in the figures) and that GRK7 and GRK1B are ‘photopic isoforms’ (for which we use red). However, we stress that this shorthand is a simplification, in part because of the occurrence of co-expression in many cases, and also because of the loss of isoforms in many species. For example, reptiles and birds have lost GRK1A and (at least in the case of chicken) their rods express GRK1B [15]. In a more extreme example, members of the Muridae family of rodents (i.e. mice and rats) have lost both GRK7 and GRK1B, so that their cones (and rods) can express only GRK1A; on the other hand, GRK7 is retained in the cone-dominant ground squirrel, a member of the Sciuridae family of rodents [16].

#### 2.1.2. Molecular phylogeny of visual G-protein receptor kinases

**Figure 1.**
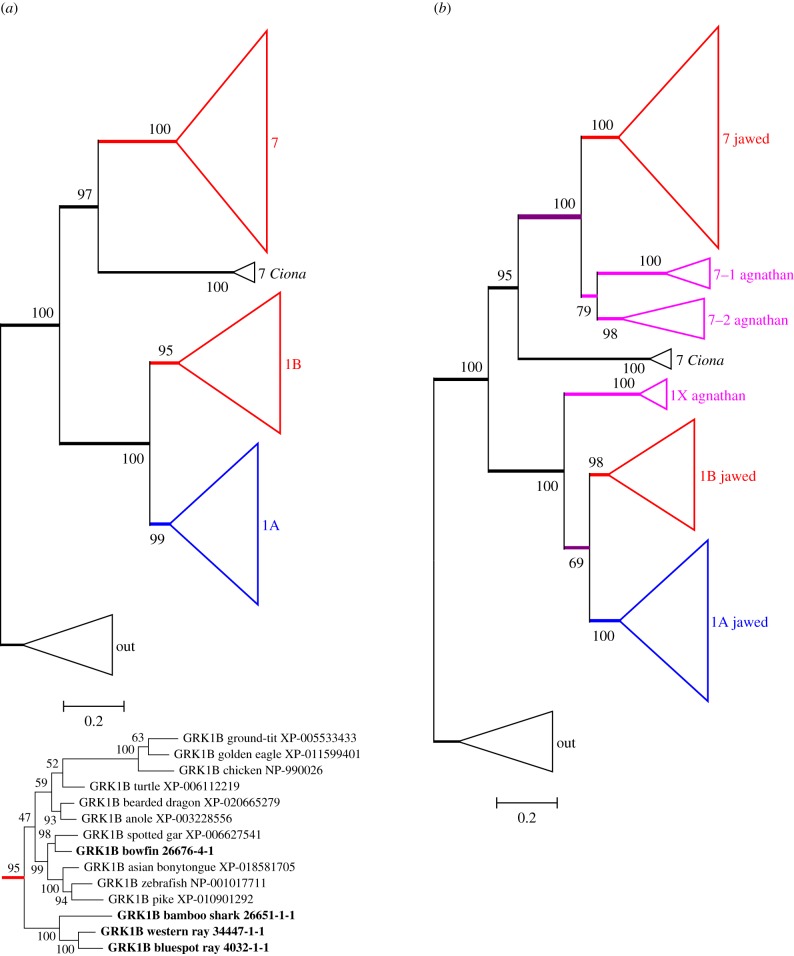
Unconstrained ML molecular phylogenies for vertebrate visual GRKs. (*a*) Jawed vertebrates; inset below shows the expanded GRK1B subtree. Colour coding for jawed vertebrate clades is according to our interpretation of the ancestral function of each GRK class: red, photopic; blue, scotopic (see text). (*b*) Jawed and agnathan vertebrates, but excluding bird GRK1 sequences. Agnathan vertebrate clades are shown as magenta. The fully expanded tree for (*b*) is presented in electronic supplementary material, figure S1. Constrained trees are presented in electronic supplementary material, figure S2.

Figure 1*a* presents the unconstrained maximum-likelihood (ML) molecular phylogeny that we obtained for photoreceptor GRKs from jawed vertebrate species, with the major clades shown in collapsed form. Four features are noteworthy. First, it is clear that there are three clades of jawed vertebrate GRKs. Second, each of these has high bootstrap support, of at least 95%. Third, a pair of *Ciona* sequences is seen to clade with the GRK7 branch with 97% support, providing strong evidence that the duplication that separated GRK7 from the pair of GRK1s predated the divergence of tunicates from proto-vertebrates. Finally, in this unconstrained tree, the three bird GRK1 sequences are placed firmly within a ‘reptilian + avian’ subtree of the GRK1B clade (see inset at the bottom of figure 1*a*). However, from the length of the avian branch, it is clear that bird GRK1B sequences are divergent. As a result, in our next step, when we also include agnathan sequences, special consideration of the bird GRK1s is required.

With the addition of 10 sequences (including our six transcripts) from agnathan vertebrates, but with the bird GRK1 sequences excluded, the unconstrained ML molecular phylogeny that we obtained is presented in figure 1*b* in collapsed form; the fully expanded tree is shown in electronic supplementary material, figure S1. The agnathan GRK sequences can be seen to form three clades (shown in magenta), distinct from the jawed vertebrate clades and each with at least 98% support. Interestingly, the three agnathan isoforms comprise a pair of GRK7s plus a single GRK1, which is inverted from the arrangement for jawed vertebrates. This tree (with the bird GRK1 sequences excluded) provides what we consider to be the most likely representation of the evolution of the GRKs that can be obtained using an unconstrained phylogeny.

When the GRK1 sequences from birds as well as agnathans were included in the phylogeny, the resulting unconstrained tree appeared implausible (see electronic supplementary material, figure S2A), with the bird and agnathan clades positioned as sisters, basal to the other GRK1B sequences. We consider this placement spurious, and we suspect that it arose from a combination of long branch attraction and convergent evolution in these distant lineages. We therefore applied a pair of constraints, to position the bird GRK1 sequences as previously obtained in the inset of figure 1*a* and the agnathan GRK1 sequences as obtained in figure 1*b*. The resulting tree is illustrated in electronic supplementary material, figure S2B; in collapsed form, it has an identical topology to that shown in figure 1*b*. The change in log likelihood elicited by these constraints was ΔLogL ≈ 9.8, and the constrained tree passed all three tests of topology, with *p*-AU ≈ 0.29, indicating that there was no reason to reject it.

We wondered whether the tendency of bird and agnathan GRK1 sequences to clade together might have been caused by the existence (in these sequences alone) of an insertion of 12 or more residues (electronic supplementary material, table S2). However, when we removed that region from the alignment, prior to tree inference, the resulting tree had identical topology and similar bootstrap support (data not shown), so we conclude that the inserts had negligible effect.

This analysis shows that when GRK1 sequences from agnathans and birds are included in the phylogeny, the unconstrained tree is suspect. One solution is to constrain the bird GRK1 sequences to clade with reptilian GRK1s. However, an alternative solution that avoids any constraints is simply to omit the bird GRK1s, as has been done in figure 1*b*.

#### 2.1.3. Interpretation of G-protein receptor kinase gene duplications and losses

**Figure 2.**
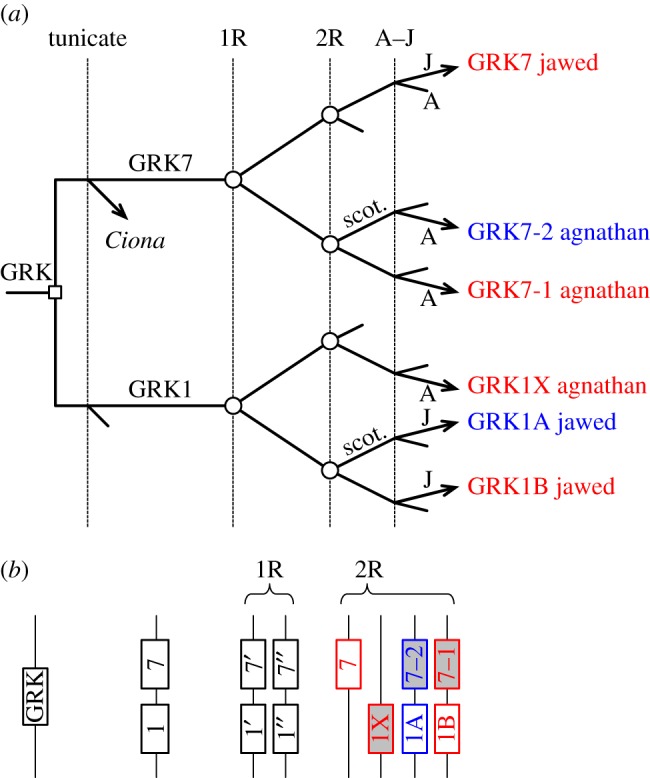
Proposed scenario for the duplications, losses and divergences underlying the expansion of visual GRKs in jawed and agnathan vertebrates. (*a*) Vertical lines denote hypothesized timing of genome duplications and speciation: 1R (first) and 2R (second) rounds of whole-genome duplication; A–J, divergence of the ancestors of extant agnathan vertebrates (A) and jawed vertebrates (J). Branch marked with open square denotes tandem gene duplication; branches marked with open circles indicate genome duplications; unmarked branches denote speciation; incomplete lines indicate gene loss. Red and blue denote presumed ancestral function in photopic and scotopic photoreception, respectively. (*b*) Diagrammatic representation of the hypothesized block arrangement of genes at four time points: ancestral; after tandem duplication; after 1R and after 2R. In the final post-2R set, grey shading denotes isoforms retained only in agnathans, whereas white denotes isoforms retained only in jawed vertebrates.

Based on the phylogeny in figure 1*b*, our proposal for the gene duplications and losses likely to have given rise to the expansion of GRK isoforms during early vertebrate evolution is presented in figure 2. We think it indisputable that the ancestral GRK1 and GRK7 originated in an ancient duplication, prior to the divergence of tunicates. It is also clear that, following 2R WGD, jawed vertebrates retained two GRK1 isoforms but only a single GRK7, whereas agnathan vertebrates retained two GRK7 isoforms but only a single GRK1. Although we favour the branching pattern indicated in figure 2, there are other possibilities that we cannot rule out. For example, agnathan GRK7-1 might be orthologous to jawed vertebrate GRK7, and agnathan GRK1X might be orthologous to jawed vertebrate GRK1B, but we consider these possibilities less likely.

A noteworthy feature of the branching pattern in figure 2*a* concerns the distribution of genes on chromosomes, as depicted in figure 2*b* at four time points. The illustrated pattern of gene losses accounts for the finding that in extant mammals, the *GRK1A* and *GRK7* genes reside on different chromosomes. Furthermore, it predicts that, for those jawed vertebrates that possess all three isoforms, the three genes (*GRK1A*, *GRK1B* and *GRK7*) would reside on different chromosomes; in a subsequent section, we shall show that this prediction holds for the spotted gar genome. And for agnathan vertebrates, it predicts that the three genes (*GRK1X*, *GRK7-1* and *GRK7-2*, shown with grey shading) would likewise reside on different chromosomes. In other words, in this illustrated scenario, none of the four duplicated blocks/chromosomes have retained both members of the ancestral tandem duplication, either in jawed vertebrates or in agnathans.

The tandem duplication that gave rise to GRK1 and GRK7 took place prior to the split between tunicates and proto-vertebrates, and hence long before the emergence of vertebrate rod photoreceptors. On the assumption (examined below) that the ancestral forms that became specialized for scotopic phototransduction were GRK1A (retained only in jawed vertebrates) and GRK7-2 (retained only in agnathans), the scenario in figure 2 is consistent with the notion that such specialization of GRK isoforms for scotopic vision did not occur until after the second round of genome duplication. This complements and extends our recent finding [7] that for the proteins of activation (i.e. the opsins, GNATs, PDE6s and CNGCs), scotopic versus photopic specialization did not arise until at least the first round of 2R.

#### 2.1.4. Expression of G-protein receptor kinase isoforms in different classes of photoreceptors

Although Osawa & Weiss [14] have listed the reported expression of visual GRK isoforms for a range of species of bony vertebrates, comparable results do not appear to be available for any species of cartilaginous fish. For jawed vertebrates, the isoform used by rods is almost always GRK1A, whereas cones of different species use either GRK7 or a GRK1, or both. We can tentatively extend that analysis to cartilaginous fish and agnathan vertebrates by examining our transcriptome data in exemplary species that lack different classes of photoreceptor. Table 1 lists the transcript levels (in RPKM calculated over the coding region) for visual GRK isoforms in four species of interest.

**Table 1.** Transcript levels (in RPKM-CDS) of GRK1/GRK7 isoforms for selected species.

	reef shark	bluespot ray	broad-gilled hagfish	short-headed lamprey
	*Carcharhinus amblyrhynchos*	*Neotrygon kuhlii*	*Eptatretus cirrhatus*	*Mordacia mordax*
opsins	Rh1, Rh2	Rh1, Rh2, LWS	Rh1 only	LWS only
GRK1A	450	172	–	—
GRK1B	—	41	—	—
GRK7	437	75	—	—
GRK1X	—	—	—	33
GRK7-1	—	—	—	28
GRK7-2	—	—	55	—

All cartilaginous fish have lost the genes for the short-wavelength opsin genes, *SWS1* and *SWS2*. The reef shark is of interest because it has additionally lost the *LWS* gene, while the fourth cone opsin (Rh2) is present at a level of approximately 5000-fold lower than for the Rh1 rod opsin (N. S. Hart 2017, personal communication). In this study, we found high levels of transcript for both GRK1A and GRK7 (table 1). We did not detect GRK1B, and although we cannot rule out its presence, its level (if present) would appear to have been at least 100-fold lower than for the other two. The simplest explanation is that reef shark rods co-express GRK1A and GRK7, and that the sparse Rh2 cones contribute negligibly to GRK transcript levels.

In the bluespot ray, it has been reported that the transcript levels for LWS and Rh2 cone opsins are approximately 100-fold lower than for the Rh1 rod opsin (N. S. Hart 2017, personal communication) and, as for all cartilaginous fish, the *SWS1* and *SWS2* genes have been lost. Here, we found moderate levels of all three isoforms of visual GRKs (table 1). Taken in conjunction with the interpretation above for reef shark, this result is consistent with the expression of GRK1B in cones (either LWS or Rh2 or both) of the bluespot ray.

For the agnathan vertebrates, the hagfish is interesting because it has only a single class of photoreceptors and only a single visual opsin, Rh1 [8,17,18]. Here, the only visual GRK we detected was GRK7-2 (table 1), indicating that the single class of hagfish (scotopic) photoreceptors most probably expresses only GRK7-2. Of the lampreys, *Mordacia mordax* is important because it also possesses only a single class of photoreceptors and only a single visual opsin, LWS [19]. Here, we detected GRK1X and GRK7-1 at comparable levels (table 1), indicating that the single class of cone-like LWS photoreceptors most probably co-expresses GRK1X and GRK7-1.

In the Discussion section, we will use these observations to propose a likely scenario for the inheritance in vertebrate species of the GRK isoforms used in photopic and scotopic phototransduction.

### 2.2. Arrestins

#### 2.2.1. Background

Arrestins mediate termination of the response and desensitization in numerous G-protein signalling cascades. Jawed vertebrate genomes typically possess four arrestin genes—*SAG* (retinal S-antigen), *ARR3*, *ARRB1* and *ARRB2*—that encode proteins that we denote, respectively, as Arr-S (expressed in rods), Arr-C (in cones), Arr-B1 and Arr-B2. These last two are often referred to as β-arrestins, though they are by no means restricted to the β-adrenergic system, and instead are widely distributed. Analysis of the phylogeny of arrestins indicates a likely origin from distantly related sequences in archaea and bacteria [20,21]. The syntenic arrangement of the genes in human and chicken is strongly suggestive of the possibility that the four members in jawed vertebrates arose during 2R [2,3], and we will examine this further for spotted gar in a subsequent section.

In jawed vertebrate photoreceptors, the ‘visual arrestins’ bind to their respective photoactivated visual pigment, in most cases after it has first been phosphorylated by a GRK, and thereby block access of the G-protein, transducin. The β-arrestins may have a similar blocking function for other activated GPCRs, but in addition they play a role in receptor internalization, mediated at least in part by a clathrin-binding site defined by the motif (Ile/Leu)_2_GlyXLeu near the C-terminus [22–24].

#### 2.2.2. Molecular phylogeny of arrestins

**Figure 3.**
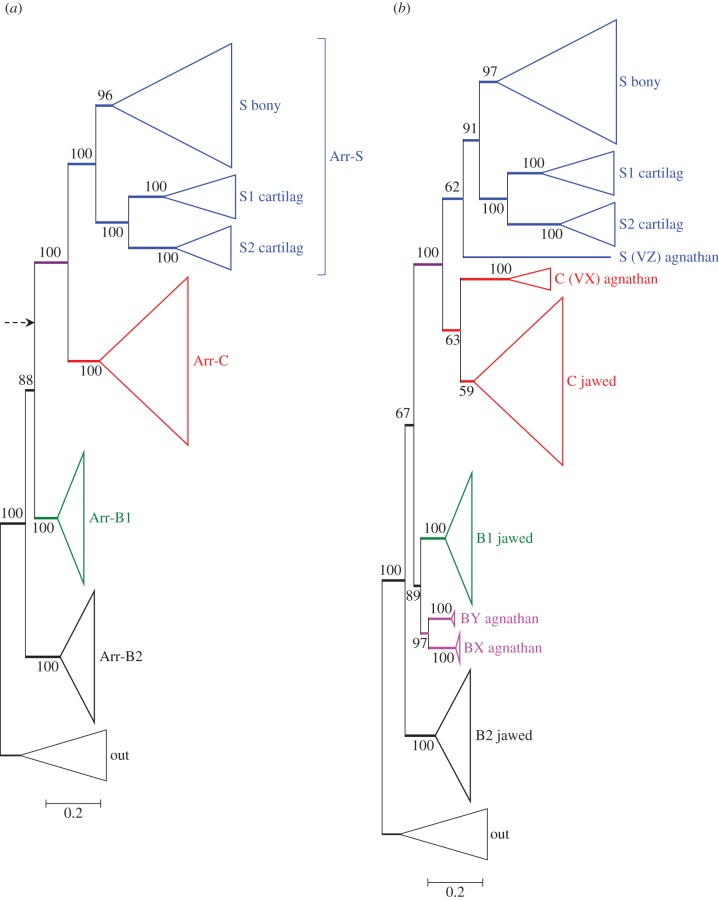
Unconstrained ML molecular phylogeny for arrestins, in collapsed form. (*a*) Jawed vertebrate arrestins. The dashed arrow shows the alternative position of the root that was sometimes found with other combinations of alignment and tree inference method. (*b*) Jawed and agnathan arrestins, excluding the single sequence, lamprey Arr-VY (see text). The fully expanded tree for (*b*) is presented in electronic supplementary material, figure S3.

We again began by restricting analysis to sequences from jawed vertebrates (together with an invertebrate out-group), and the resulting unconstrained tree is presented in figure 3*a*. Perhaps surprisingly, the branching pattern does not conform to the simplest model of 2R quadruplication, and instead, Arr-B2 is positioned basally, though with only 88% support. With other combinations of alignment or tree inference method, the root could sometimes be positioned, as indicated by the arrow, in a more conventional 2R configuration, but with lower support. When we recomputed these trees with the root constrained to the alternative position indicated by the arrow, the resulting change in score was small (ΔLogL < 4) and the constrained tree passed all tests of phylogeny, with *p-*AU > 0.2. We conclude that both branching patterns are entirely plausible, either with Arr-B2 having diverged prior to 2R or with all four isoforms having diverged during 2R.

One notable feature of the tree for jawed vertebrate arrestins in figure 3*a* (and, indeed, for the trees that we subsequently obtained upon inclusion of agnathan sequences) is the existence in all six species of cartilaginous fish of a pair of Arr-S clades, labelled Arr-S1 and Arr-S2. This is strong evidence for duplication of the Arr-S gene in a stem cartilaginous fish. In our four species (two sharks and two rays), the levels of transcript in the retina were 2 to 10 times higher for Arr-S1 than for Arr-S2, and for the three species in which we detected Arr-C, its level was lower than for Arr-S2 (see electronic supplementary material, table S1). This suggests that the isoform used in the rods of cartilaginous fish is likely to be Arr-S1, and that Arr-S2 may play a role elsewhere than in photoreceptors.

**Figure 4.**
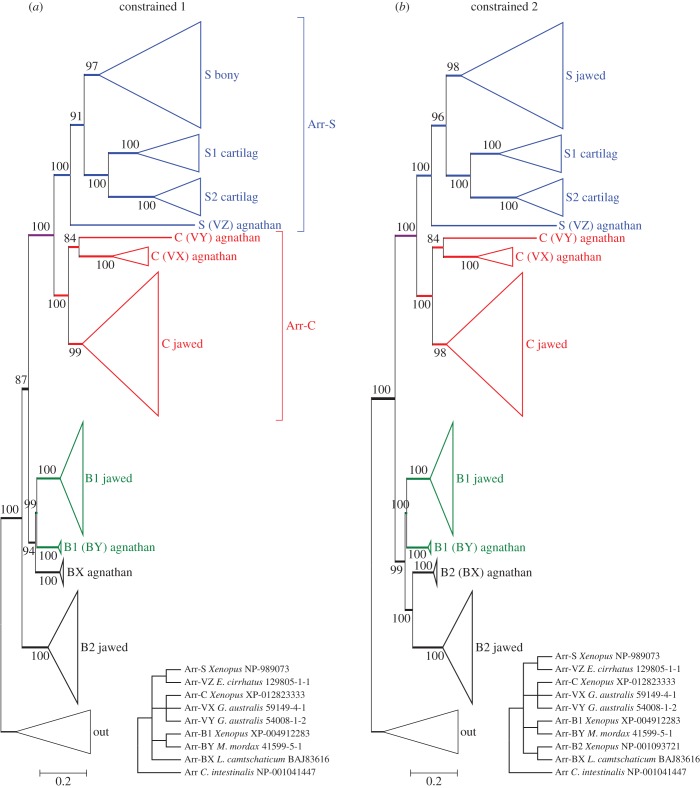
Constrained molecular phylogenies for vertebrate arrestins, in collapsed form; the lamprey Arr-VY sequence has been included. Both panels represent constraints that are consistent with 2R genome duplication, followed by A–J speciation. (*a*) With no constraint on jawed vertebrate Arr-B2. (*b*) With jawed vertebrate Arr-B2 also constrained. Both trees passed all three tests of topology, with *p*-AU = 0.25 (*a*) and 0.18 (*b*).

Our unconstrained phylogenetic analysis, with all but one of the agnathan arrestin sequences included, is shown in collapsed form in figure 3*b* and fully expanded in electronic supplementary material, figure S3. We had noted an issue of apparent attraction between the hagfish visual arrestin and one of the lamprey visual arrestins that we suspect arose from the divergent nature of the hagfish sequence. Accordingly, we tried omitting either or both of these sequences with the following results. Figure 3*b* presents the unconstrained ML tree when the lamprey sequence (Arr-VY) was omitted; it shows the hagfish sequence (Arr-VZ) clading with jawed vertebrate Arr-S sequences, as would be expected given that the hagfish has Rh1 as its only opsin. When, instead, the hagfish Arr-VZ was omitted and the lamprey Arr-VY was included, the resulting tree had high support and placed all four lamprey visual arrestins with the jawed vertebrate Arr-C sequences (data not shown). But when the hagfish Arr-VZ sequence and the lamprey Arr-VY sequence were both included, those two sequences were placed together, within the Arr-C clade, though with low support (data not shown). In the analysis of functional motifs that we present in §2.7, we will see that the lamprey Arr-Y sequence has features of an Arr-C, whereas the hagfish Arr-Z has features of an Arr-S. Therefore, our view is that their tendency to clade together within the Arr-C subtree is artefactual; this artefact could be avoided either by omitting the single lamprey Arr-Y sequence (figure 3*b*), or by constraining the hagfish Arr-Z sequence to clade with Arr-S (figure 4).

In the unconstrained tree of figure 3*b*, the topology of the visual arrestin sequences appears entirely reasonable. On the other hand, the placement of the lamprey β-arrestins (Arr-BX and Arr-BY) as sisters is not easy to reconcile with 2R genome duplication. It could, for example, be interpreted to suggest that an agnathan-specific duplication occurred after the agnathan–jawed (A–J) vertebrate split, or alternatively that the unconstrained ML tree does not accurately reflect the duplications that occurred during 2R. To try to distinguish between these possibilities, we tested a number of different constraints on the positions of the agnathan clades, consistent with models of 2R duplication followed by A–J speciation; in all cases, we included both the Arr-VY and Arr-VZ sequences.

Figure 4 presents the two constrained phylogenies that had the highest probability according to the approximately unbiased test. (One point to note in all constrained trees is that the level of bootstrap support at node(s) that have been constrained is necessarily increased, on occasion to 100%.) In both cases, we had constrained hagfish Arr-VZ with Arr-S, lamprey visual arrestins with Arr-C and lamprey Arr-BY with Arr-B1, as indicated in the two insets. The difference between the two panels is that in figure 4*a*, we placed no constraint on Arr-B2, whereas in figure 4*b* we constrained Arr-B2 with lamprey Arr-BX. The differences in log likelihood (from the unconstrained ML tree) were ΔLogL ≈ 13.4 in figure 4*a*, and 20.8 in figure 4*b*, and both trees passed all three tests of topology, with *p-*AU ≈ 0.25 in figure 4*a* and 0.18 in figure 4*b*, indicating that both trees represent acceptable descriptions of the data. Other constrained ‘2R’ topologies failed at least two of the three tests (e.g. with the positions of Arr-BX and Arr-BY interchanged, or with either of these agnathan beta arrestins constrained with Arr-B2 in the basal position).

#### 2.2.3. Interpretation of arrestin gene duplications and losses

**Figure 5.**
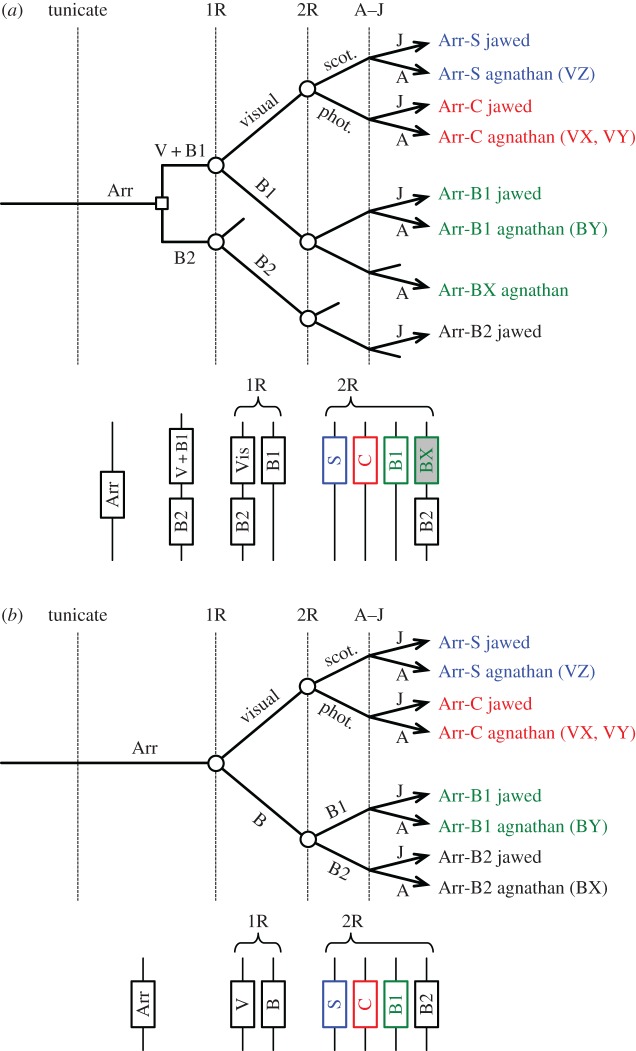
Two possible scenarios for the duplications, losses and divergences, underlying the expansion of arrestins in jawed and agnathan vertebrates, corresponding to the constrained phylogenetic trees in figure 4*a,b*. In each panel, the lower diagram shows the pattern of genes on blocks/chromosomes, at the indicated time points.

In the light of these analyses, figure 5 presents the two scenarios for the evolution of vertebrate arrestins shown by the phylogenies in figure 4 that were consistent with 2R genome duplication and that passed the tests of topology. As we cannot be certain that either of these is the ‘correct’ scenario, we have deemed it best to retain our non-judgemental annotation of agnathan isoforms. Nevertheless, we think it highly probable that the following are orthologues: agnathan Arr-VZ = jawed Arr-S and agnathan Arr-VX = jawed Arr-C. However, the identity of the remaining agnathan isoforms Arr-VY, Arr-BX and Arr-BY is less clear-cut, and they need not necessarily be orthologous to jawed vertebrate isoforms. Importantly, though, our analysis shows that scotopic versus photopic specialization of arrestin did not occur until after the second round of 2R genome duplication.

**Figure 6.**
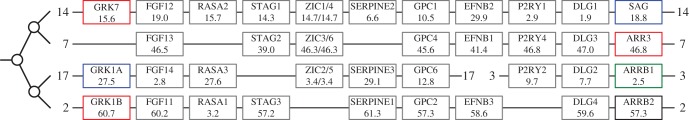
Syntenic arrangement of genes in the vicinity of the visual *GRK*s and arrestins, for spotted gar linkage groups LG14, LG7, LG17/LG3 and LG2. It is clear that the sets of genes form a paralogon, with one set split across LG17 and LG3. Numbers below the gene names give positions on the respective LG group in Mb. Only genes with at least three paralogues located on these linkage groups are shown; a number of additional gene families have two members on these linkage groups. In the spotted gar assembly, the *ARR3* gene is partial.

The lower section in each panel of figure 5 shows the corresponding block arrangements of genes for the pattern of duplication in the upper section, at several time points. In both cases, the post-2R pattern conforms to the syntenic arrangement described originally by Nordstrom *et al*. [2] (their Figure 8) and Larhammar *et al*. [3] (their Figure 5), and extended below (figure 6), in which the four jawed vertebrate arrestin genes reside in a paralogon on different chromosomes.

### 2.3. Gene synteny for visual G-protein receptor kinases and arrestins

The syntenic arrangement of spotted gar genes in the vicinity of visual *GRK* genes and arrestin genes is shown in figure 6. This diagram represents the spotted gar equivalent of the synteny for chicken and human genes presented by Larhammar *et al*. [3] in their Figure 5. We have chosen to analyse spotted gar because, in this species, all three visual *GRK* genes and all four arrestin genes are present. (In the current assembly, *Arr3*, which encodes Arr-C, is partial; but for the phylogenies above, our transcriptome data provided a full-length Arr-C for Florida gar.) Eleven gene families comprising at least three members are illustrated, and in addition, several more pairs of genes were found but are not shown.

From figure 6, it is clear that the illustrated chromosomal regions, linkage groups LG14, LG7, LG17/LG3 and LG2, form a paralogon. In addition, by applying our interpretations of the phylogenies derived in figures 2 and 5, we can specify the 1R and 2R branchings, as indicated at the left. Importantly, the pattern of chromosomal 2R pairings is found to be consistent between the *GRK* phylogeny (figure 2) and the arrestin phylogeny (figure 5).

### 2.4. Regulator of G-protein signalling, RGS9

#### 2.4.1. Background

We now turn our attention to the components that mediate shut-off of the activated G-protein, transducin. These comprise a complex of three proteins: RGS9 (regulator of G-protein signalling 9), Gβ5 (the so-called fifth class of G-protein β subunit) and R9AP (the anchor protein for RGS9). The rod and cone photoreceptors of any given species are thought to express a common form of the triplet RGS9–Gβ5–R9AP.

The primary function of RGS9 is to upregulate the GTPase activity that is intrinsic to the G-protein α subunit, so as to accelerate hydrolysis of GTP's terminal phosphate, and thereby provide rapid shut-off of activated transducin. RGS9 sequences display two main variants: a photoreceptor-specific short isoform, termed RGS9S or RGS9-1 (of approx. 480 residues), and a long isoform termed RGS9L or RGS9-2 (of approx. 670 residues), which is more broadly distributed; both are encoded by alternate splicing from a single gene. In phototransduction, the GTPase activity is maximally accelerated upon formation of the complex (Gα·GTP–PDE*γ*)–(RGS9S–Gβ5–R9AP). In other systems, it has been postulated that the C-terminal extension of the long RGS9L may perform a comparable role to that played by PDEγ in the bound form PDEγ–RGS9S.

RGS9 is a member of the R7 family of RGS proteins, comprising RGS6, RGS7, RGS9 and RGS11. RGS9 and RGS11 are closely similar (as described below) and both are expressed in the retina, with RGS9 restricted to photoreceptors, and RGS11 present in the synaptic regions of inner retinal neurons; see, for example, [25]. RGS6 and RGS7, which are more distantly related, are also both expressed in the inner retina.

#### 2.4.2. Molecular phylogeny of RGS9

**Figure 7.**
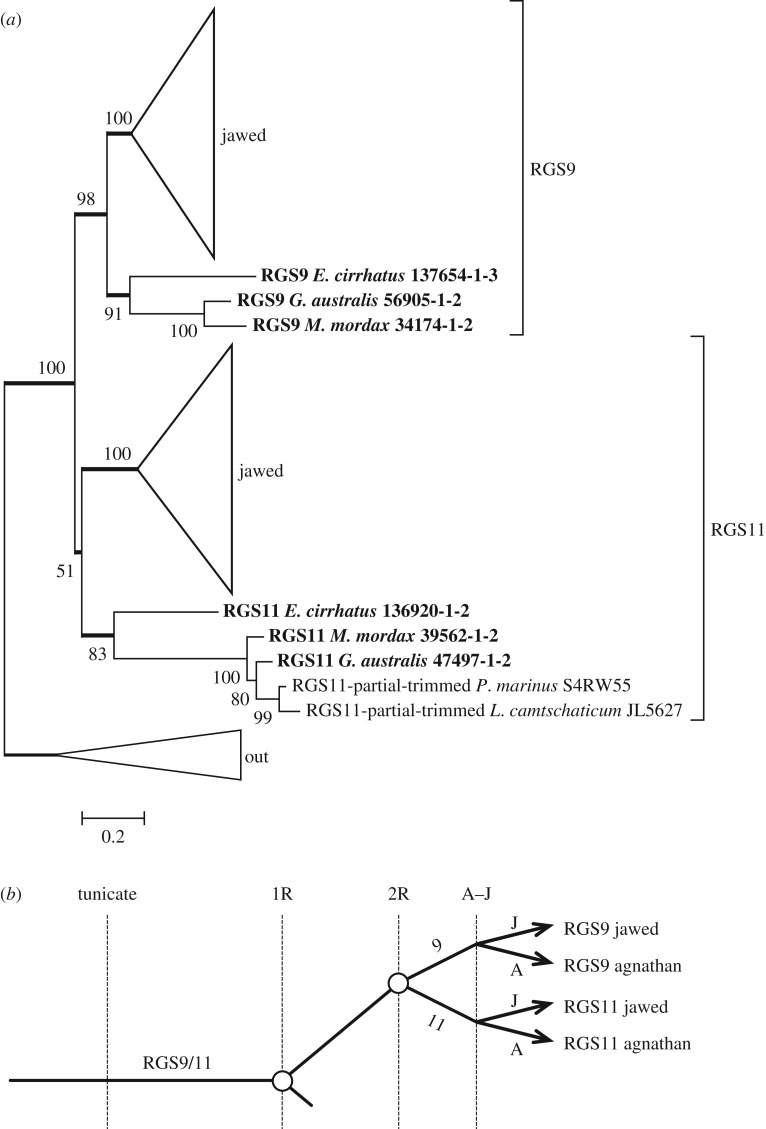
Molecular phylogeny for vertebrate RGS9 and RGS11. (*a*) Unconstrained ML phylogeny, with jawed vertebrate subtrees collapsed; the fully expanded tree is shown in electronic supplementary material, figure S4. (*b*) Proposed scenario for the origin of RGS9 and RGS11.

For phylogenetic analysis of RGS9 proteins, we used only the short (RGS9S or RGS9-1) photoreceptor form; the only amphibian RGS9 we could locate was from *Xenopus laevis* and so we used that species. The resulting molecular phylogeny that we obtained for jawed and agnathan vertebrate sequences is shown in figure 7*a* with the jawed vertebrate subtrees condensed; the fully expanded tree is presented in electronic supplementary material, figure S4. The branch at the base of the RGS11 subtree is very short and poorly supported (51%), suggesting the possibility that the agnathan ‘RGS11’ clade ought instead to have been placed basally. We tested that possibility by constraining the clade to be basal (not shown) and found that the change in log likelihood was very small, at ΔLogL < 1, and that the resulting tree passed the three tests of topology. The same (collapsed) topology was obtained whether the aligner was MAFFT or ClustalW, and whether the substitution model was WAG or LG, though in the three cases other than that illustrated in figure 7*a*, the level of support for the RGS11 subtree was marginally higher (56–61%).

We interpret these findings to indicate that, based on the data we have, the two possible positions for the agnathan ‘RGS11’ clade are almost equally likely. However, the topology shown in figure 7*a* is more parsimonious, in requiring fewer gene losses. We think that an ambiguity in the placement of the ‘agnathan RGS11’ clade is just what might be expected if the A–J split had occurred very soon after the duplication that generated RGS9 and RGS11. Accordingly, we propose that the RGS9/RGS11 duplication occurred at the second round of 2R, as illustrated by the schematic in figure 7*b*. However, this proposal is tentative, and it would be useful to examine synteny to test the possibility further.

### 2.5. G-protein beta subunit 5, Gβ5

Each of the members of the R7 family of RGS proteins (RGS6, RGS7, RGS9 and RGS11) forms an obligate complex with Gβ5, which is encoded by the *GNB5* gene. Despite its name, Gβ5 does not appear to associate with G-proteins and is only distantly related to the four conventional G-protein β subunits, Gβ1–Gβ4.

**Figure 8.**
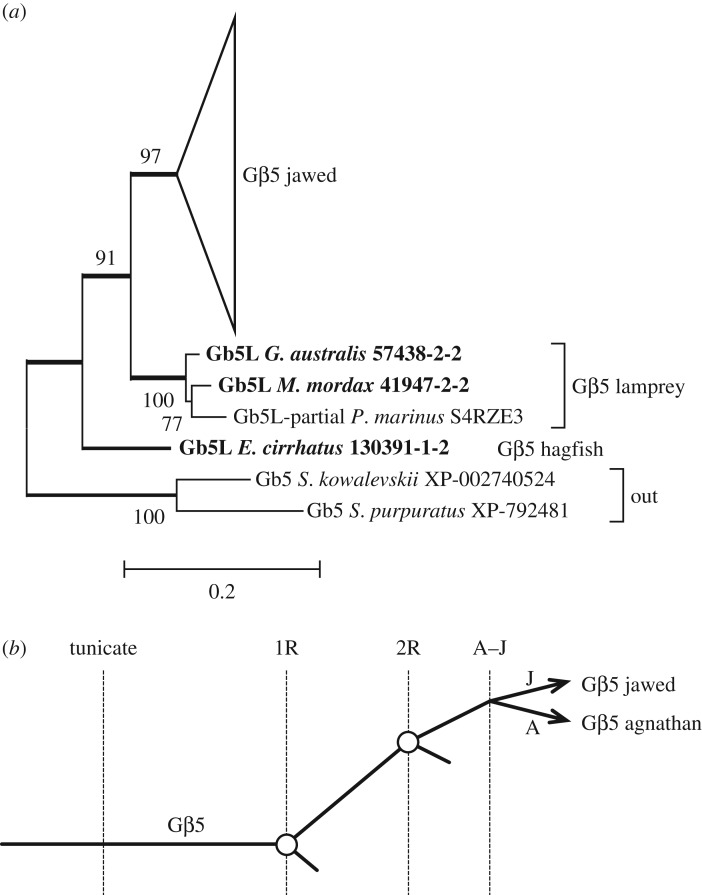
(*a*) Unconstrained molecular phylogeny for Gβ5, with the jawed vertebrate subtree collapsed. The fully expanded tree is presented in electronic supplementary material, figure S5. (*b*) Simple scenario for the origin of Gβ5.

Figure 8*a* presents the molecular phylogeny that we obtained for Gβ5; the fully expanded tree is shown in electronic supplementary material, figure S5. The jawed vertebrate subtree shows a high degree of conservation, with a mean within-group distance of less than 0.1 substitutions per residue. The lamprey sequences formed a unanimously supported clade, but the single hagfish sequence was placed as sister to the lamprey and jawed vertebrate groups. Unless the position of the hagfish sequence genuinely reflects a duplication, this phylogeny provides no evidence for a duplication of the *GNB5* gene within the chordate lineage. The highly conserved nature of the protein is consistent with the need for this single protein to interact with the four members of the R7 RGS family. A straightforward model of 2R gene duplication and loss is shown in figure 8*b*.

### 2.6. RGS9 anchor protein, R9AP

#### 2.6.1. Background

For each of the four members of the R7 group of RGS proteins, the RGS–Gβ5 complex is tethered to the membrane by one of two anchor/binding proteins: R7BP (encoded by *RGS7BP*) or R9AP (encoded by *RGS9BP*). In vertebrate photoreceptors, this anchor protein is R9AP, and the tethered complex RGS9S–Gβ5L–R9AP interacts with activated transducin, Gα·GTP–PDE*γ*, to trigger its shut-off.

In the NCBI database, there are three related ‘R9AP’ proteins, typically named R9AP, R9AP-B and R9AP-like. Most taxa possess at least two of the corresponding genes, and several possess all three; on the other hand, almost all placental mammals appear to possess only the first. Taxa that have been shown to possess all three isoforms include amphibians (*Xenopus tropicalis* and *Nanorana parkeri*), coelacanths (where the R9AP-B isoform is partial) and bony fish (spotted gar, zebrafish and Asian bonytongue). To date, there is just a single species of placental mammal, the degu (*Octodon degus*), that has been shown to possess the R9AP-like isoform.

The gene tree in Ensembl likewise suggests three subtrees, and the three gene families generally have zero, two and one intron(s), respectively, within the coding region; the third gene (that encodes the R9AP-like isoform) has an additional intron in the 5′-UTR, and many teleost species exhibit two additional introns. Interestingly, the Ensembl gene tree lists no taxa outside of vertebrates, and the only sequences we found that were sufficiently close to use as an out-group were from basal deuterostomes (a hemichordate and an echinoderm).

#### 2.6.2. Molecular phylogeny of R9AP

**Figure 9.**
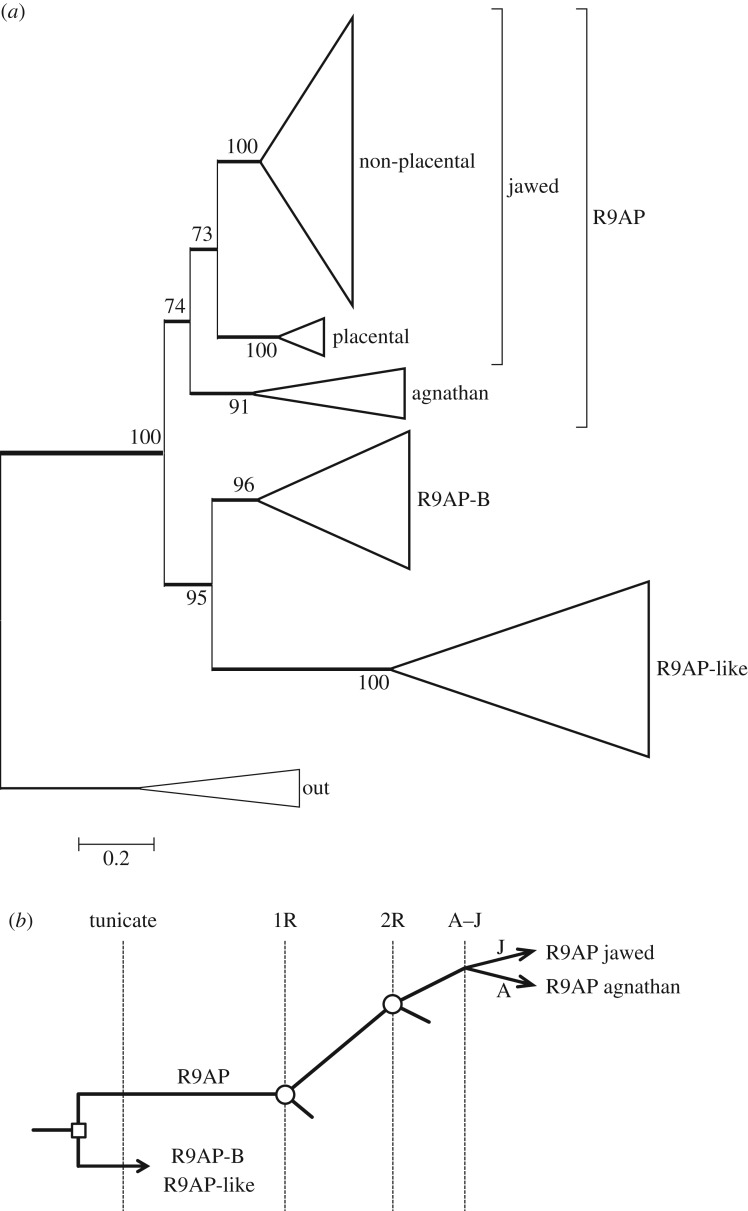
(*a*) Unconstrained molecular phylogeny for R9AP. The fully expanded tree is presented in electronic supplementary material, figure S6. (*b*) Proposed scenario for the origin of R9AP isoforms.

The molecular phylogeny that we obtained for R9AP and its homologues is presented in figure 9*a*; the fully expanded tree is shown in electronic supplementary material, figure S6. Although the sequences for the first variant (R9AP) from jawed vertebrates form a clade with unanimous support, that clade contains a subtree for placental mammals that appears divergent from the subtree for all other jawed vertebrates, including non-placental mammals. In every phylogeny that we calculated, these two subtrees were separated by a distance of greater than 0.3 substitutions per residue.

The four agnathan sequences form a tight clade, which in unconstrained trees was sometimes placed as shown, but in other analyses was alternatively placed as sister to the subtree for placental mammals, though with low support. In each case, when we constrained the agnathan clade to the alternate position, the resulting change in log likelihood was very small (ΔLogL ≈ 1.5) and the tree passed all three tests of topology (data not shown). Therefore, although we cannot assign the position of the agnathan clade with certainty, we think that the tree in figure 9*a* is likely to represent the true branching pattern.

To examine whether the three isoforms of R9AP might have arisen during 2R WGD, we inspected surrounding genes, in the genomes of three species that possess all three isoforms; namely: spotted gar (on linkage groups LG23, LG9 and LG18), *X. tropicalis* (on scaffolds GL172730, GL173397 and GL172665) and zebrafish (on chromosomes 18, 7 and 6). However, in none of these cases were we able to find evidence for a paralogon. Although zebrafish possessed a number of pairs of genes on chromosomes 18 and 7, there was negligible correlation with chromosome 6, and we suspect that the pairs arose from 3R duplication. Finally, examination of the phylogeny in figure 9*a* shows the net distances between the three paralogues to be larger than is typical for members of a paralogon. We therefore think it likely that the three ‘R9AP’ isoforms originated prior to 2R WGD, as sketched in figure 9*b*.

### 2.7. Functional motifs of agnathan versus jawed vertebrate proteins

In this section, we consider the conservation, between agnathan and jawed vertebrates, of functional motifs in the proteins.

#### 2.7.1. Functional motifs of visual G-protein receptor kinases

**Figure 10.**
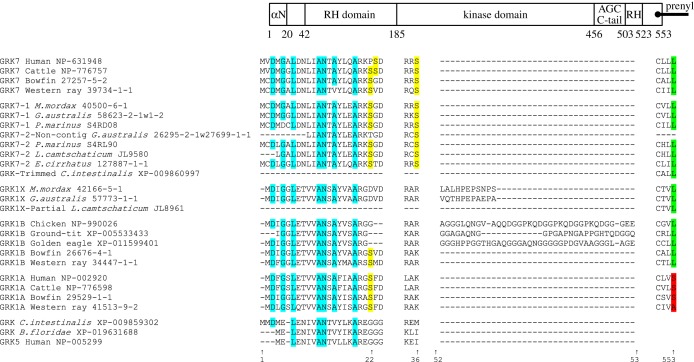
Comparison of residues between visual GRK isoforms for a selection of taxa and positions. Schematic at top shows overall organization of the GRK1/GRK7 protein molecule. All numbering is for human GRK7. Cyan highlighting indicates the presence of the expected residue at the seven sites implicated in the binding of recoverin/*S-*modulin. Yellow highlighting indicates the Ser residues (at sites 22/23 and 36) that are subject to phosphorylation by PKA. The next segment shows the insertions (found immediately after the residue corresponding to 52 in human GRK7) in lamprey GRK1Xs and avian GRK1Bs. At the far right, green highlighting denotes that the terminal residue of the ‘CaaX’ prenylation motif is Leu (which provides the signal for geranylgeranylation), whereas red denotes a Ser or Ala (which provides the signal for farensylation). The entire alignment for all GRK sequences is shown in electronic supplementary material, table S2, and modelled molecular structures are presented in electronic supplementary material, figure S7.

Figure 10 presents a schematic overview of the structure of the visual GRKs (see [11]), together with the residues present at a number of key sites for several jawed vertebrates and each of the agnathan vertebrates. The entire alignment for all the GRK sequences we examined is presented as electronic supplementary material, table S2.

**Figure 11.**
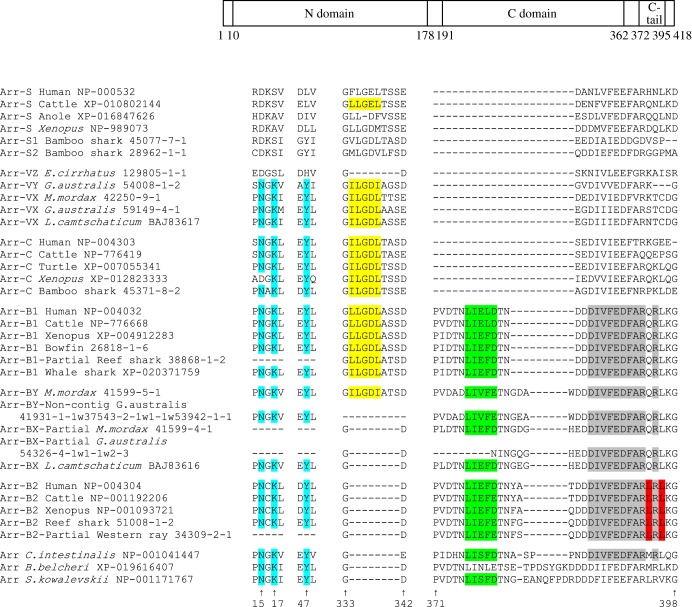
Comparison of residues between arrestin isoforms for a selection of taxa and positions. Schematic at top shows overall organization of arrestin protein molecule. All numbering is for human Arr-B1. Cyan highlighting indicates the ‘NKY’ H-binding network in the N-terminus (note that the residue numbering in this region is one greater than in bovine Arr-C numbering). The remaining sections are in the C-terminus. Yellow highlighting indicates the ‘(I/L)_2_GX(I/L)’ clathrin-binding motif located within an 8-residue splice loop. This splice loop is generally present in the visual arrestins, and in Arr-B1 and Arr-BX, but it is missing from Arr-B2; it is also missing from *E. cirrhatus* Arr-VZ (and from gecko Arr-C; see electronic supplementary material, table S3). Green highlighting indicates the ‘LϕXϕ(D/E)’ clathrin-binding motif, where ϕ is a bulky hydrophobic residue and X is any polar amino acid. Grey highlighting indicates the binding site for the clathrin adapter AP-2. Red highlighting indicates the Leu-rich nuclear export signal, NES. The entire alignment for all arrestin sequences is shown in electronic supplementary material, table S3, and modelled molecular structures are presented in electronic supplementary material, figure S8.

For all GRKs, the enzyme's kinase domain is inserted within an ‘RH domain’ (RGS homology domain), so-called because of its homology to regulator of G-protein signalling proteins (see top of figure 10). The core catalytic domain comprises a small N-terminal lobe, a large C-terminal lobe and an AGC tail. The small lobe consists of five-stranded β-sheets and a conserved helix, αC, whereas the large lobe is largely α-helical. The active kinase site is located at the interface between the lobes, with the small lobe providing a nucleotide-binding pocket and the large lobe providing the phospho-acceptor-binding site. The RH domain contains approximately 150 amino acids that form a bundle of nine helices, followed by a 10th helix after the kinase domain, a structure that is conserved in all RGS proteins [26]. The specificity of interaction with target GPCRs is determined by the RH domain, and although the regions critical for receptor interaction have been identified in the case of mammalian GRK1A in a study by He *et al*. [27], their analysis did not extend to examining the specificity of GRK7 versus GRK1A.

The N-terminus forms an α-helical region (αN in figure 10, top). Domain mapping has shown that the first 30 residues are not required for interaction with the GPCR [27]. On the other hand, seven sites in this region have been shown to be involved in the interaction with recoverin/*S*-modulin [28]. For the visual GRK sequences that we obtained, we found the amino acids present at these seven sites to be quite highly conserved (cyan highlighting in figure 10), across both jawed and agnathan vertebrates.

GRKs can be subject to phosphorylation by cAMP-dependent protein kinase (PKA), resulting in reduced efficacy of GRK phosphorylation of the GPCR. *In vitro* analysis has identified targets for this process as Ser residues at site 21 in GRK1A, and at sites 22/23 and 36 in GRK7 [29]; the PKA consensus motif has the form ‘RaS’ or ‘RaaS’. The yellow highlighting in figure 10 shows that Ser residues at sites 21 or 22 are absent from bird and agnathan GRK1 sequences, but present in all other visual GRK sequences that have coverage in this region, apart from *Geotria australis* GRK7-1, which has a Thr. In addition, Ser36 is universally present in GRK7 sequences of jawed and agnathan vertebrates, but absent from all GRK1s. The presence of the two corresponding targets in the GRK7s of agnathan and jawed vertebrates indicates an ancient origin for the sites of Ser phosphorylation in visual GRKs.

An intriguing feature of the GRK1 sequences for birds and lampreys is the occurrence of an insertion at a common location in the N-terminal region of the RH domain. As shown in figure 10, this insertion for the three avian GRK1B sequences is between 26 and 37 residues in length, while for the two lamprey GRK1X sequences (that were represented in this region), the insert is only 12 residues in length. When we modelled these sequences onto a common template sequence (PDB reference 4pni) using the SWISS-MODEL application (swissmodel.expasy.org; see Material and methods), we found that the inserts were predicted to form an extended coil within the RH domain (electronic supplementary material, figure S7).

The C-terminus of each of the visual GRKs ends with a four-residue prenylation motif ‘CaaX’, where the final residue ‘X’ specifies the moiety to be attached to the Cys residue, thereby mediating membrane binding. As shown at the far right of figure 10 (and more comprehensively in the complete alignment in electronic supplementary material, table S2), the final residue ‘X’ was L (Leu, highlighted in green) for every visual GRK sequence other than GRK1As (i.e. for every GRK1B and GRK7, including the two *Ciona* GRK7s). For the GRK1As, the final residue was A, S or C (Ala, Ser or Cys), highlighted in red. This indicates that, whereas the GRK1As are anchored by a farensyl moiety, the GRK1Bs and the GRK7s are anchored by a geranylgeranyl moiety. It is possible that this distinction relates to the fact that, in rods, GRK1A is anchored to the pinched-off disc membranes, whereas in cones it is the plasma membrane to which the proteins are anchored. None of the out-group sequences (including another *Ciona* GRK) contained a prenylation motif at the C-terminus.

#### 2.7.2. Functional motifs of visual arrestins

The rod arrestin molecule (figure 11*a*) is composed of two domains (N- and C-domains), each of which consists of a seven-stranded β sandwich, comprising four- and three-stranded β sheets packed against each other [30]. Using SWISS-MODEL, we have modelled the visual arrestins we obtained from agnathan vertebrates and confirmed that this basic structure is retained (electronic supplementary material, figure S8).

Mammalian cone arrestins possess an H-binding network resulting from the ‘NKY’ motifs Asn14, Lys16 and Tyr46, in the numbering of bovine Arr-C [31]. As illustrated in figure 11*b* (and electronic supplementary material, table S3), we found this motif in all cone arrestins, β-arrestins and members of the out-group, indicating that it is most probably the ancestral state. By contrast, this H-binding network was not present in any rod arrestins, with the equivalent sites occupied by Asp14, Ala/Gly/Ser16 and Leu/Tyr46 (although in a few species Ile, Gln, Phe or Val was found at site 46). All of the lamprey (but not hagfish) visual arrestins have the ‘NKY’ motif (figure 11*b*) and would therefore be expected to possess the H-binding network. The hagfish Arr-VZ sequence has Asp14 and Ser16, as found in the rod isoforms, but His46 rather than Leu46; it therefore lacks the H-binding network, consistent with our identification of hagfish Arr-VZ as orthologous to Arr-S.

The C-termini of arrestin isoforms differ in the motifs they contain [32]. For example, in contrast to visual arrestins, both classes of β-arrestin contain motifs involved in binding clathrin and the clathrin adaptor, AP-2 [33,34], while Arr-B2 contains a Leu-rich nuclear export signal, NES, that is involved in docking of c-Jun N-terminal protein kinase, JNK3 [35]. In addition, there is an 8-residue splice loop that is generally present in Arr-B1 and visual arrestins, but absent from Arr-B2 and the out-group sequences. This loop is also present in the only agnathan Arr-BY with coverage in this region; however, it is missing from agnathan ArrBX and from *Eptatretus cirrhatus* Arr-VZ (and also from gecko Arr-C; see electronic supplementary material, table S3). Within this loop, there is typically an ‘(I/L)_2_GX(I/L)’ motif (yellow highlight) that is implicated in clathrin binding. The β arrestins also contain an ‘LϕXϕ(D/E)’ clathrin-binding motif (green highlighting), where ϕ is a bulky hydrophobic residue and X is any polar amino acid. In addition, the β arrestins contain a binding site for the clathrin adapter AP-2 (grey highlighting). Finally, the Arr-B2 sequences contain the NES motif (red highlighting). Figure 11 shows these final three motifs to be absent from the visual arrestins of agnathan vertebrates, cartilaginous fishes and bony fishes, supporting our identification of those sequences as visual arrestins.

All cone arrestins (Arr-C) possess both the H-binding network (cyan) and the (I/L)_2_GX(I/L) motif (yellow), apart from amphibian Arr-Cs which alone among Arr-Cs lack Asn14 (figure 11; electronic supplementary material, table S3). Likewise, all four lamprey arrestins (the three Arr-VXs and the single Arr-VY) possess these same motifs, whereas the hagfish Arr-VZ possesses neither. Therefore, despite the tendency of the lamprey Arr-VY and hagfish Arr-VZ sequences to clade together, the presence and absence of the two motifs provides strong support for our identification of Arr-VY as Arr-C and Arr-VX as Arr-S.

#### 2.7.3. Functional motifs of RGS9 and its binding protein

The major domains of the RGS9 protein are a DEP domain (residues 22–109) necessary for interaction with R9AP [36,37], a GGL domain (residues 221–280) and a canonical RGS domain (residues 292–412). In all cases, the agnathan proteins show extensive sequence conservation over these regions.

R9AP and its relative, R7BP, both possess an N-terminal tri-helical region and a heptad/SNARE motif [38–40]. However, they differ in that R9AP possesses a C-terminal transmembrane segment that serves to anchor the protein to the photoreceptor membrane. This domain, which stretches from residues 208 to 234, is highly conserved across all jawed vertebrates, but shows divergence in the agnathan species, with 17–18 of the 27 residues substituted. Nevertheless, when analysed by the TMHMM program (see Material and methods), these agnathan sequences are predicted to form a transmembrane domain and therefore to provide a membrane anchor.

## 3. Discussion

Our phylogenetic analyses, combined in two cases with syntenic analyses, have permitted us to determine the likely patterns of gene duplication and loss in the several protein families involved in termination of the vertebrate photoresponse. We next draw together an overview of the coevolution of these components, and then we discuss the features of our analysis that we consider to have been important in reaching our conclusions. Then, for the GRKs, we present some new perspectives on the inheritance (and loss) of isoforms in different vertebrate lineages.

### 3.1. Coevolution of the shut-off components of vertebrate phototransduction

**Figure 12.**
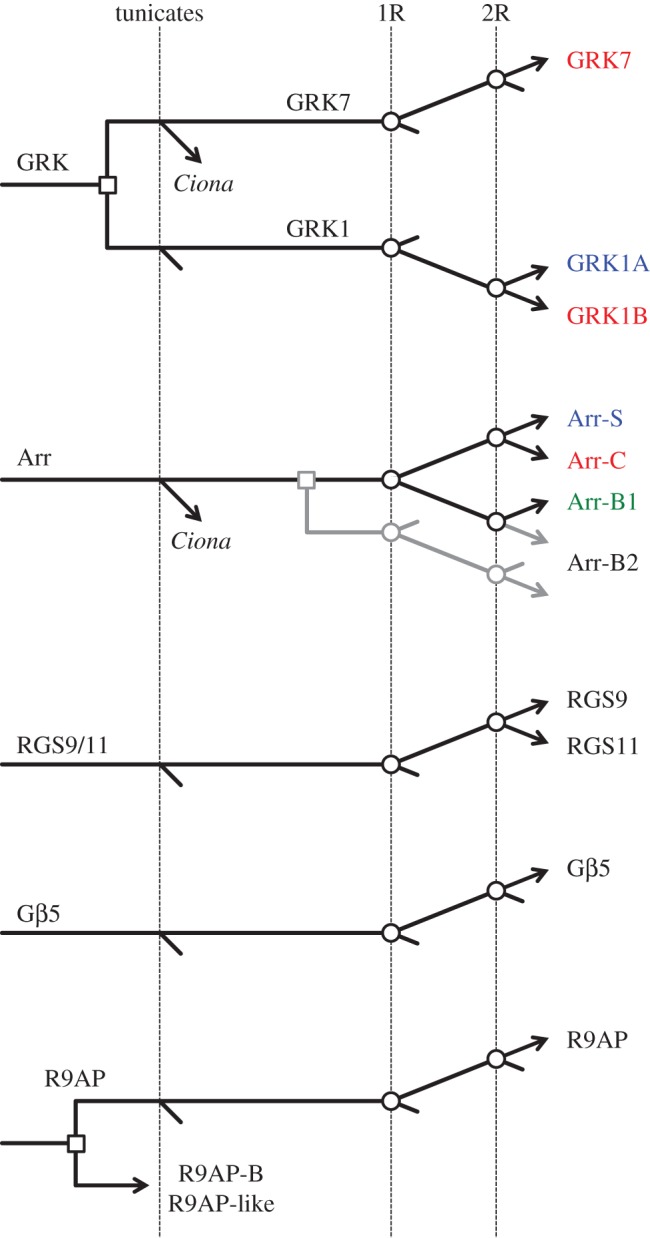
Gene duplications and losses hypothesized to have occurred during 2R. The first vertical line indicates divergence of the tunicate lineage from the stem vertebrate lineage. The subsequent two vertical lines indicate the two whole-genome duplications, labelled 1R and 2R, for the first and second rounds, respectively. For arrestins, the grey lines indicate two alternative possibilities for the origin of Arr-B2. Agnathan vertebrates are not shown here, but can be found in the preceding individual scenarios. The time scale is arbitrary. Open squares denote individual (tandem) gene duplications, whereas open circles denote genome duplications.

The analyses we have presented in this paper provide an account of the coevolution of the proteins mediating the shut-off steps of vertebrate phototransduction, as summarized in figure 12.

For the first class of the proteins mediating shut-off of activated visual pigment, namely the GRKs, expansion occurred through a duplication prior to 2R as well as through the two rounds of genome duplication. This led to the utilization of three distinct visual GRKs (GRK7, GRK1A and GRK1B) in jawed vertebrate cones and rods and, as shown in figure 2, to an alternative set of three distinct visual GRKs (GRK7-1, GRK7-2 and GRK1X) in agnathan photoreceptors.

For the second class of the proteins mediating shut-off of activated visual pigment, the arrestins, expansion clearly occurred during the two rounds of genome duplication. In addition, the phylogenetic analysis (figures 3–5) suggested that Arr-B2 might have diverged from the other arrestins prior to 2R. Although cursory examination of the paralogon arrangement in figure 6 might suggest that the four arrestins arose through 2R quadruplication, this conclusion cannot, in fact, be drawn. Thus, the syntenic arrangement in figure 6 does not rule out the possibility of a local (e.g. tandem) gene duplication prior to 2R, then expansion followed by gene loss, that left only a single member of the original tandem pair on each of the four blocks. Accordingly, we cannot distinguish between the two possible branching patterns of figure 5 on the basis of the gene synteny in figure 6.

For the triplet of proteins mediating shut-off of the activated G-protein transducin, namely RGS9-Gβ5L-R9AP, a common set of isoforms is used across placental mammals. Our evidence suggests that RGS9 probably diverged from RGS11 at the second round of 2R. On the other hand, Gβ5 did not expand during 2R. For R9AP, we cannot rule out the possibility that the expansion into a trio of isoforms occurred during 2R, but this looks unlikely. Only one of the isoforms, R9AP, is used in mammalian phototransduction, and it has not yet been established how or indeed whether the other two isoforms, R9AP-B and R9AP-like, participate in phototransduction in other taxa.

An important interpretation from this analysis is that the isoforms of shut-off proteins that are specialized for scotopic (low-light) vision, GRK1A in jawed vertebrates or GRK7-2 in agnathans, and Arr-S in both, did not emerge until the second round of whole-genome duplication. This is even later than for the proteins mediating activation of the cascade, where we recently showed that the scotopic isoforms Rh1, GNAT1, PDE6A/B and CNGA1 arose at the first duplication [7]. Despite differences in their overall sensitivity, mammalian rods and cones exhibit quite similar activation parameters [41], and the substantially speedier responses of cones compared with the sluggish responses of rods result from considerable differences in the parameters of the shut-off steps. Accordingly, our analysis supports the idea that the ancestral cones and the precursors of rods were likely to have exhibited very similar response kinetics until after the second round of WGD. In the relatively short time that elapsed before the A–J speciation event, each of the phototransduction proteins appears to have undergone fairly minor changes in the scotopic branch relative to the photopic branch. This further supports our recent proposal that little of the specialization that now distinguishes rod and cone transduction was present when agnathans diverged, and that instead much of the ‘duplex’ rod/cone specialization evolved subsequently.

### 3.2. Strengths and weaknesses of our phylogenetic analyses

A number of factors have contributed to the ability of our phylogenetic analyses to determine what we regard as the likely patterns of gene duplication and loss. First, we think it important that we included a substantial number of sequences from across a diverse set of vertebrate taxa, and especially that we were able to use several representatives from each of two basal groups: agnathan vertebrates and cartilaginous vertebrates. Second, our work benefitted from the use of two freeware packages available for use on personal computers: SATé [9] for multiple sequence alignment and IQ-Tree [10] for phylogenetic tree inference. Our subjective impression is that IQ-Tree undertakes a more thorough search for the maximum-likelihood tree than some other packages do; and in addition, we found its ultrafast bootstrap approximation [42] to be more than an order of magnitude faster than conventional methods. Third, in examining potential models of gene duplication for consistency (or otherwise) with 2R genome duplication, we consider it very important to have been able to apply constraints to the tree inference procedure, followed by the application of tree topology tests to the resulting constrained trees, and in this regard we found IQ-Tree to be ideal.

Uncertainties in our analyses pertain primarily to the branching pattern for agnathan members. This was a particular problem for hagfish sequences, where in many cases we had only a single member (from *E. cirrhatus*) in any class, and sometimes that was a partial sequence. This problem was exacerbated by the fact that hagfish apparently diverged from lampreys relatively soon after their common ancestor diverged from what would become jawed vertebrates, with the consequence that each of our hagfish sequences has diverged substantially from its nearest relative. Because of this issue, we omitted several hagfish sequences that were placed on their own on very long branches; as far as we could tell, omission of these hagfish sequences did not disrupt the overall topology of the trees (data not shown).

A further complication with the inclusion of agnathan taxa was that the resulting agnathan clades in the ML tree sometimes appeared subject to ‘mutual attraction’, presumably as a result of their long branches, and possibly also as a result of compositional differences compared with jawed vertebrate proteins; for example, lamprey proteins frequently had strings of G (Gly) in close proximity (see, e.g. electronic supplementary material, table S2). However, these agnathan branch attractions appeared rather weak, as constraining the positions of agnathan clades to conform with plausible patterns of 2R duplication resulted in only small changes in log likelihood (from the ML tree) and tests of topology gave no grounds for rejecting the constrained tree.

The phylogenetic position of bird and agnathan GRK1 sequences (§2.1.2) provides an example of the strengths and weaknesses of our approaches. In our unconstrained analysis of jawed vertebrate sequences, the clade of three avian GRK1Bs was positioned with reptilian GRK1Bs, though on a long branch (inset to figure 1*a*). Given that this topology conforms very closely with expectations from species phylogeny, we think it very likely to be correct. However, in some preliminary attempts, this avian clade was placed in an unexpected position, basal to the other GRK1Bs (data not shown), in what we presume to have been an artefact resulting from its long branch length. This situation was exacerbated when we included agnathan sequences, as the avian and agnathan GRK1 sequences were placed as sisters, and jointly as sister to the remaining GRK1Bs, but with low support (electronic supplementary material, figure S2A). When we then omitted the bird GRK1B sequences, the resulting unconstrained tree (electronic supplementary material, figure S1) placed the agnathan clade basally and provided very high support (98%) for the jawed vertebrate GRK1B clade. Thus, it was only when *both* bird and agnathan GRK1 sequences were included that the phylogeny showed unexpected results, combined with low bootstrap support.

This was one circumstance in which the ability to constrain the phylogeny was very important. With sequences from birds and agnathans included, we constrained chicken and turtle GRK1B to be sisters, and GRK1Bs to be sister to GRK1As (see inset in electronic supplementary material, figure S2B), whereupon the topology became highly plausible, and tests of topology gave no grounds for rejecting the constrained tree (*p*-AU ≈ 0.29). In this case, all five nodes within the GRK1 subtree showed unanimous support, no doubt due to the constraints that we had applied.

As a final point in relation to the phylogeny of bird and agnathan GRK1s, we tested whether the existence of an insertion in these sequences (figure 10; electronic supplementary material, table S2) might have contributed. However, when we removed that region from the alignment, prior to tree inference, the resulting tree had identical topology and similar support (data not shown), so we reject the notion that the inserts had much effect on the tree.

### 3.3. Inheritance of G-protein receptor kinase isoforms in photopic and scotopic phototransduction

We now discuss the conclusions that can be drawn about the inheritance of GRK isoforms in vertebrate taxa, by combining data from the literature with our new description for GRK gene duplications and losses (§2.1.3) and our results on the expression (or apparent absence) of isoforms in agnathan species (§2.1.4). First, it is clear that there are numerous examples across disparate species of both jawed and agnathan vertebrates where photoreceptors co-express two visual GRKs.

In considering this, we need to recall that the tandem duplication that gave rise to GRK1 and GRK7 took place prior to the split between tunicates and proto-vertebrates (figures 2 and 12), and hence long before the emergence of vertebrate rod photoreceptors. To account for the widespread co-expression of GRKs in extant species, the most parsimonious explanation is that, prior to the 2R duplication, the ancestral cone-like photoreceptors of proto-vertebrates co-expressed both members of the ancestral pair of visual GRKs (GRK1 and GRK7). Furthermore, we propose that the multiple classes of photoreceptor in descendent organisms generally inherited the property of co-expression of one GRK1 and one GRK7, though in some cases the ability to express one member of the pair was lost, and, in other cases, one of the genes was lost. Based on these concepts, table 2 presents our description of the manner in which co-expression of the different isoforms has been inherited (or lost) in a variety of taxa of interest.

**Table 2.** Proposed inheritance, and loss, of co-expression of visual GRK isoforms in photopic and scotopic photoreceptors of extant taxa. ✓, protein is expressed in this class of photoreceptors; –, loss of expression of this isoform in this class; ✗, loss of gene from this taxon; →, expression of alternate isoform following loss of gene; N, loss of this class of photoreceptors; ?, uncertain.

	rods		cones	
jawed vertebrates	GRK1A	GRK7	GRK1B	GRK7
ancestral	✓	✓	✓	✓
cartilaginous fish	✓	✓	✓	?
bony vertebrates	✓	–	✓	✓
birds	✗→1B	–	✓	✓
mammals	✓	–	✗→1A	✓
pig, dog	✓	–	✗	✓
murid rodents	✓	✗	✗→1A	✗

	rod-like		cone-like	
agnathan vertebrates	GRK1X	GRK7-2	GRK1X	GRK7-1
ancestral	✓	✓	✓	✓
lampreys	✓	✓	✓	✓
*M. mordax*	✗N	✗N	✓	✓
hagfish	×	✓	✗N	✗N

In the stem jawed vertebrate lineage, we propose that the three remaining post-2R visual GRKs were expressed according to the ancestral arrangement of one GRK1 and one GRK7, as follows: cones and rods both expressed the single GRK7, with cones co-expressing GRK1B and rods co-expressing GRK1A. It is on this basis that we denote GRK1B as ‘photopic’ (red) and GRK1A as ‘scotopic’ (blue). In the cartilaginous fish lineage, this arrangement continued (with the proviso that we have no reliable information about the expression of GRK7 in the cones of extant cartilaginous fish). In the bony vertebrate lineage (i.e. jawed vertebrates excluding cartilaginous fish), we propose that the primary change was that rods ceased to express GRK7 (indicated by dash in table 2) and therefore expressed GRK1A alone. Subsequently, several exceptions arose. Thus, birds and reptiles lost the *GRK1A* gene (cross in table 2), and their rods instead co-opted the remaining GRK1, GRK1B, which elsewhere has a photopic function. Mammals, on the other hand, lost the *GRK1B* gene, with extant mammals expressing one or both of GRK7 and GRK1A. We suggest that this began as co-option of the alternative GRK1, GRK1A, along with GRK7 in cones, and that subsequently some species (e.g. pig and dog) dispensed with co-expression of GRK1A in cones, whereas others (e.g. primates) continued. In murid rodents, the *GRK7* gene has additionally been lost, leaving their cones expressing GRK1A alone, as in their rods.

### 3.4. Conclusion

The principal outcome of our work has been the formulation of a description, shown in figure 12, for the likely pattern of duplications and losses in the genes involved in shut-off of vertebrate phototransduction. Of particular note is our conclusion that the rod isoforms of GRK and arrestin did not emerge until the second round of whole-genome duplication. This has the implication that little of the specialization that now distinguishes rod and cone phototransduction was present when agnathans diverged, and that instead much of the rod versus cone specialization of jawed vertebrates evolved subsequently.

## 4. Material and methods

### 4.1. Transcriptome data

The methods for obtaining the eye transcriptomes from basal vertebrate species were described by Lamb *et al.* [8], and here we use transcripts from that work. Sequences were available for each of the following species obtained from Australian waters: *E. cirrhatus*, broad-gilled hagfish; *G. australis*, pouched lamprey; *M. mordax*, short-headed lamprey; *Aptychotrema vincentiana*, western ray; *Aptychotrema rostrata*, eastern ray; *Neotrygon kuhlii* (*N. australiae*), bluespot ray; *Chiloscyllium punctatum*, bamboo shark; *Carcharhinus amblyrhynchos*, reef shark. Sequences were also obtained from bowfin, *Amia calva*, and Florida gar, *Lepisosteus platyrhincus*. Searching of our transcriptomes was performed using a custom program, TriPyGDU [8], and augmented using a Blast server, SequenceServer [43]. Here, we report 99 new sequences, which have been submitted to GenBank and assigned nucleotide accession numbers MG063622–MG063720.

In two cases for *G. australis*, we merged non-overlapping sequences to create a non-contiguous sequence. In these cases, we ensured that (i) the transcript levels of the component sequences were similar; (ii) the component sequences exhibited a high level of identity to a single complete sequence from a closely related species and (iii) the resulting joined sequence exhibited a high level of identity to that same sequence. (We additionally formed a non-contiguous sequence for eastern ray RGS9, which, apart from the gap, was identical to the complete sequence for western ray, and was therefore not used; see below.) These three ‘non-contiguous joined’ sequences are marked ‘NJ’ in electronic supplementary material, table S1.

### 4.2. Sequence selection

We tried to use as uniform a set of taxa as possible, aiming to select: two placental mammals (human and cattle), two marsupials, three birds, three reptiles, two amphibians, bowfin and gar; two sharks, two rays and elephant shark (a chimaera). For eastern and western ray, the orthologous sequences were identical (or nearly so) when we had both, and in those cases, we used only the western ray sequence. Likewise, for the two species of gar, we used only the Florida gar sequence when we had nearly identical orthologues. For agnathan vertebrates, we used every available sequence, except for those partial sequences that we deemed to be too short (e.g. less than about half the expected length). For several partial sequences from agnathan species, we noted a deterioration of the alignment near the end of the sequence. In these cases, we removed the poorly aligned terminal residues; these sequences are listed as ‘Trimmed’ in the figures. We also substantially trimmed the *Ciona* GRK sequence XP_009860997 (figure 1; electronic supplementary material, figure S1), because the C-terminal half failed to align with other GRK sequences. For out-groups, we searched for closely similar sequences from tunicates (*Ciona intenstinalis* and *C. savigni*) and lancelets (*Branchiostoma floridae* and *B. belcheri*), and from two other more basal deuterostomes (*Strongylocentrotus purpuratus*, an echinoderm, and *Saccoglossus kowalevskii*, a hemichordate), as well as from the fruit fly, *Drosophila melanogaster*.

We sometimes encountered problems with the placement of coelacanth (*Latimeria chalumnae*) and hagfish (*E. cirrhatus*) sequences as ‘single taxon clades’ in positions that did not fit with accepted descriptions of species phylogeny. In most cases, we therefore excluded coelacanth sequences. In addition, we excluded two hagfish β-arrestins that caused problems for the alignments and that appeared to be far more divergent than other β-arrestins; likewise, we omitted one highly divergent hagfish R9AP.

### 4.3. Multiple sequence alignment

We performed multiple sequence alignment of protein sequences using SATé-II (v. 2.2.7) [9]. For the illustrated phylogenies, we standardized on the following settings: aligner, MAFFT; merger, MUSCLE; tree estimator, FASTTREE; model, WAG + G20; decomposition, centroid; maximum sub-problem size, 12. We generally obtained very similar results using ClustalW as the aligner, but these are not illustrated. To avoid introducing bias, we did not manually adjust any alignments, and we always used the entire alignment. The alignments we obtained are presented in electronic supplementary material, tables S2–S6.

### 4.4. Tree inference

We constructed unconstrained ML phylogenetic trees using IQ-Tree (Windows multicore v. 1.5.5, [10]), using the ultrafast bootstrap approximation [42]. For the phylogenies presented, we standardized on the following settings: 10 000 bootstrap replicates; protein substitution model, WAG [44]. We generally obtained very similar results using the LG substitution model [45], but these are not illustrated. Numbers at each node represent percentage bootstrap support.

Constrained trees were constructed using the ‘-g’ constraint option in IQ-Tree. In specifying the constraints, we used the minimum set of sequences that would constrain the tree as we intended. Typically, we used just a single sequence representative of the relevant isoform, and we relied on the tightness of clading to constrain the other orthologues in the same manner. Each constraint tree that we used is shown as an inset by the constrained tree. One point to bear in mind when examining constrained trees is that the level of bootstrap support at any node that has been constrained is necessarily (i.e. artificially) increased, in many cases to 100%, because of the constraint.

For each constrained tree obtained, we conducted tree topology tests using the ‘-z’ option in IQ-Tree, in order to test whether or not the constrained tree needed to be rejected in comparison with the unconstrained ML tree. The tests applied were *bp*-RELL, *c-*ELW and *p-*AU, representing, respectively, the bootstrap proportion test using the RELL method [46], the expected likelihood weight test [47] and the approximately unbiased test [48]. Only those trees that passed all tests at the 95% confidence level (i.e. *p* ≥ 0.05) were considered further.

### 4.5. Molecular modelling

The existence of transmembrane domains was predicted using the ‘Prediction of transmembrane helices in proteins' program, TMHMM v. 2 (www.cbs.dtu.dk) [49]. The structure of GRKs and arrestins was predicted using SWISS-MODEL (swissmodel.expasy.org) [50]. Protein sequences were used to search for appropriate templates; the same template was then used for each sequence in the class.

## Supplementary Material

Supplementary Figures

## Supplementary Material

Supplementary Tables

## Data accessibility

The data supporting the findings of this article have been uploaded as part of the electronic supplementary material, and mRNA sequences have been deposited in GenBank with accession numbers MG063622–MG063720.
