# Supplementary material for: Evolution of the shut-off steps of vertebrate phototransduction
Source: Open Biol. 2018 Jan 10;8(1):170232. doi: 10.1098/rsob.170232 (PMC5795056; doi:10.1098/rsob.170232)
Supplement: Supplementary Figures [file rsob170232supp1.pdf]

Supplementary Figures
for

Evolution of the shut-off steps of vertebrate phototransduction

Trevor D. Lamb[1], Hardip R. Patel[2], Aaron Chuah[3], and David M. Hunt[4,5]

[1]Eccles Institute of Neuroscience, [2]National Centre for Indigenous Genomics, and [3]Genome Discovery Unit, John Curtin School of Medical Research, The Australian National University, ACT 2600, Australia, and [4]The Lions Eye Institute and [5]School of Biological Sciences, The University of Western Australia, WA 6009, Australia.

Contents

Page

Supplementary Figure S1. Fully-expanded unconstrained molecular phylogeny for vertebrate GRKs excluding birds GRK1s, corresponding to the collapsed tree shown in Fig. 1B. 2

Supplementary Figure S2. Molecular phylogeny for vertebrate GRKs, including GRK1 sequences from birds and agnathans, in collapsed form. 3

Supplementary Figure S3. Fully expanded unconstrained molecular phylogeny for jawed and agnathan vertebrate arrestins, corresponding to the collapsed tree shown in Fig. 3B. 4

Supplementary Figure S4. Unconstrained molecular phylogeny for vertebrate RGS9 and RGS11, corresponding to Fig. 7A. 6

Supplementary Figure S5. Fully expanded molecular phylogeny for jawed and agnathan vertebrate Gβ5 subunits, corresponding to the collapsed tree shown in Fig. 8A. 7

Supplementary Figure S6. Fully expanded molecular phylogeny for R9APs, corresponding to the collapsed tree shown in Fig. 9A. 8

Supplementary Figure S7. Molecular models of GRK isoforms. 9

Supplementary Figure S8. Molecular models of visual arrestin isoforms. 10

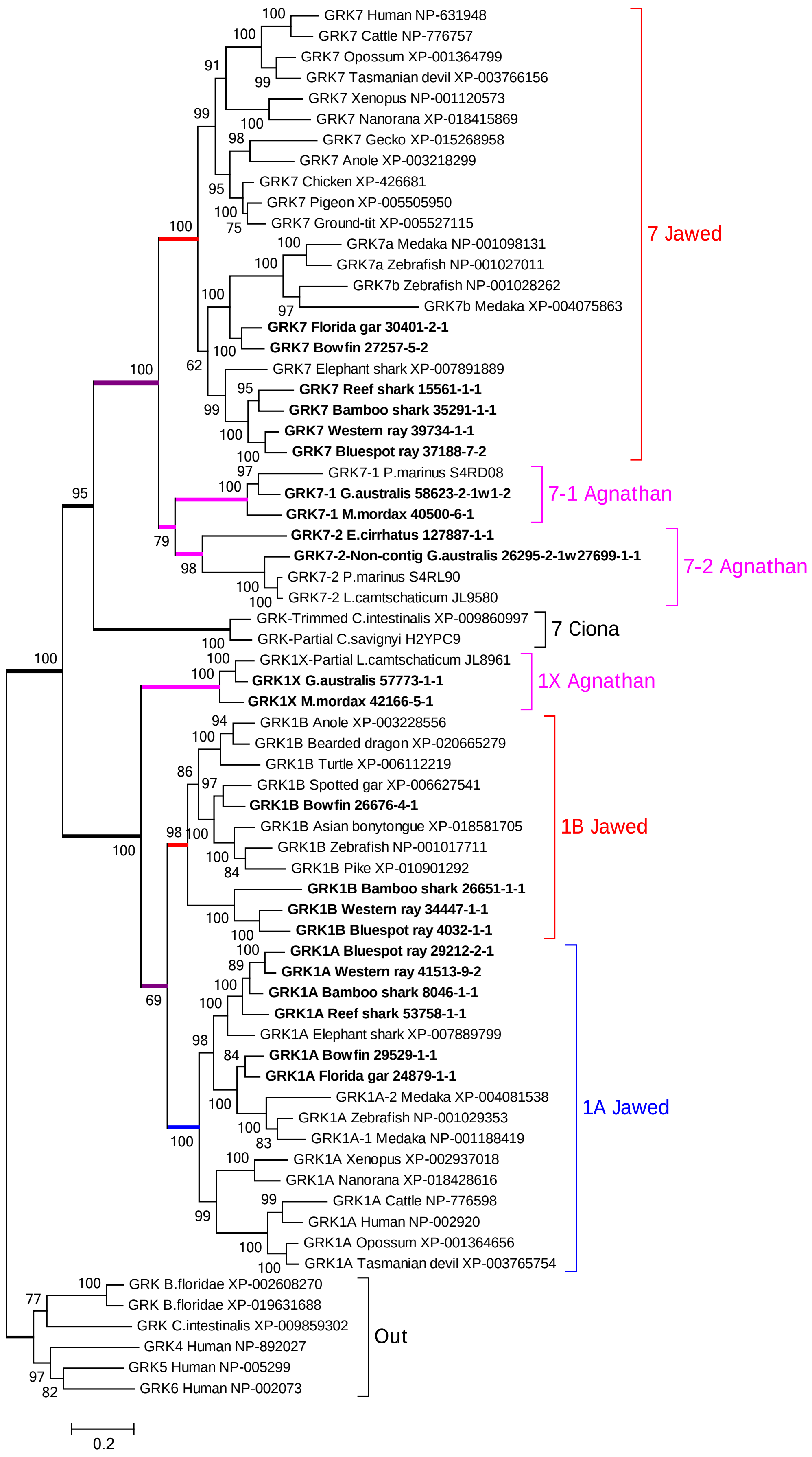

Supplementary Figure S1. Fully-expanded unconstrained molecular phylogeny for vertebrate GRKs excluding birds GRK1s, corresponding to the collapsed tree shown in Fig. 1B.

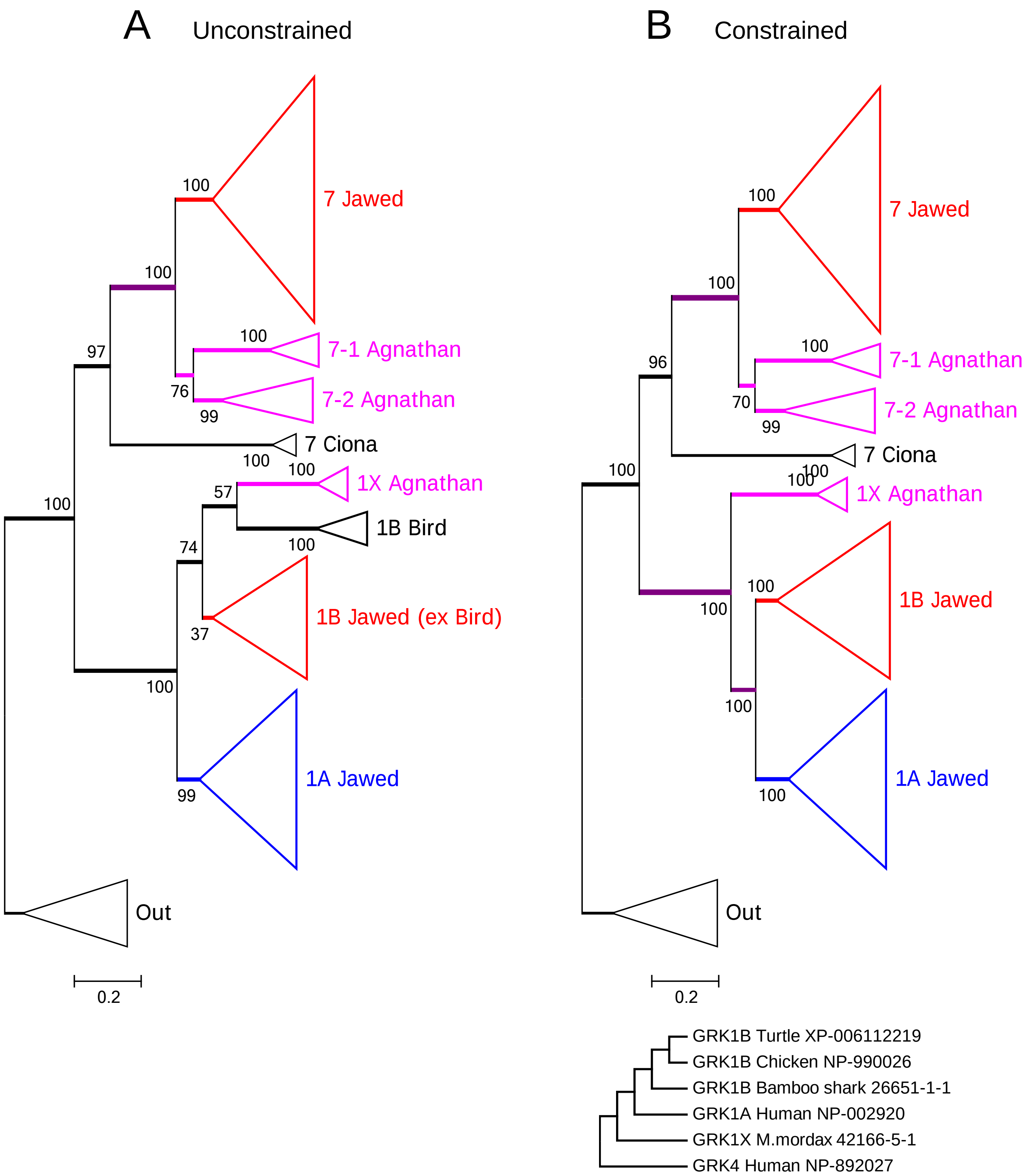

Supplementary Figure S2. Molecular phylogeny for vertebrate GRKs, including GRK1 sequences from birds and agnathans, in collapsed form. A, Unconstrained. B, Constrained to conform to the collapsed tree shown in Fig. 1B, where bird GRK1 sequences had been omitted and no constraint had been applied. Constraint tree is shown below. The constrained tree in B differed in log likelihood from the unconstrained tree in A by ΔLogL $\approx$ 9.8, and it passed the three tests of topology, with *p*-AU $\approx$ 0.29.

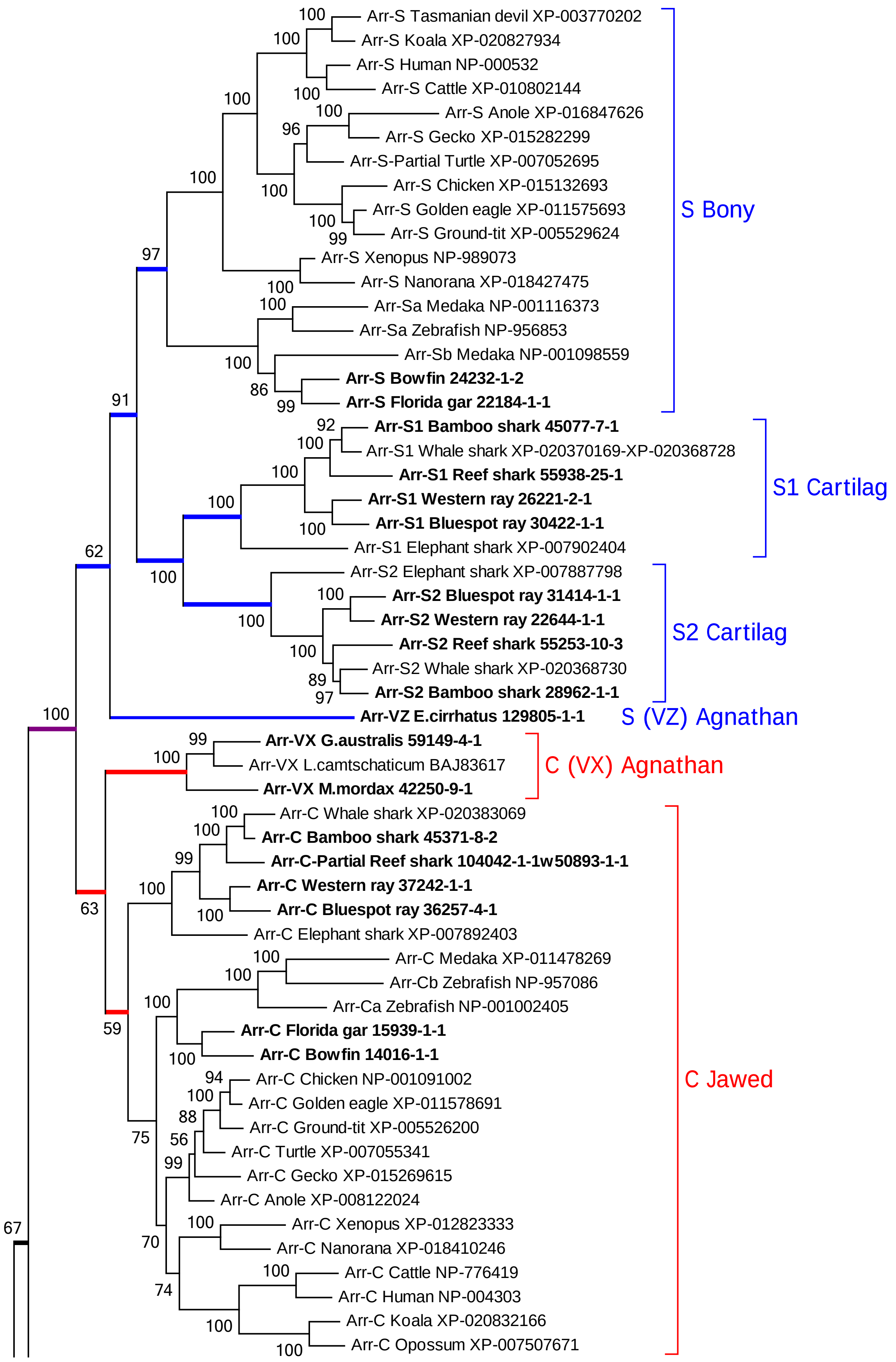

Continued next page …

Continued from previous page …

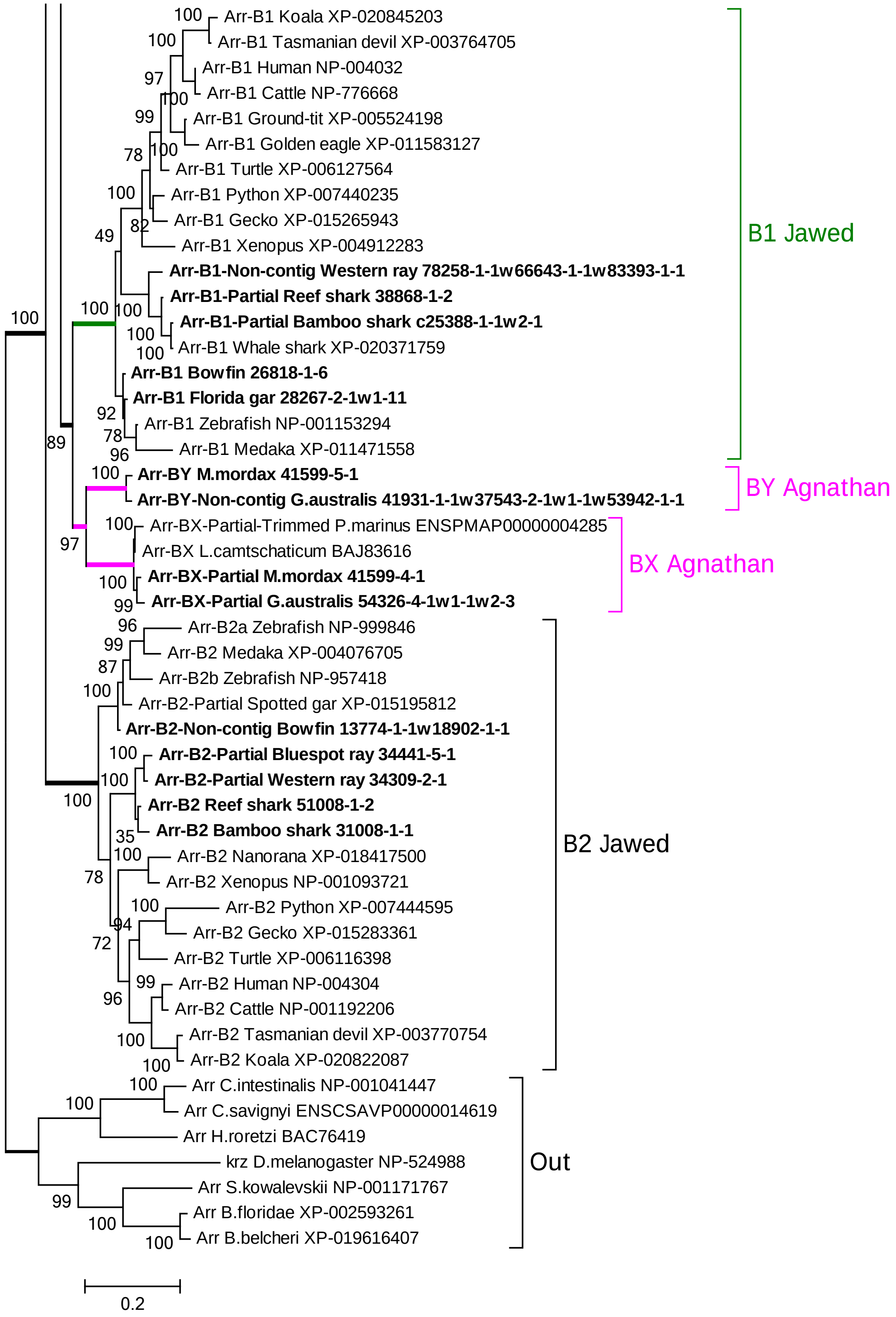

Supplementary Figure S3. Fully expanded unconstrained molecular phylogeny for jawed and agnathan vertebrate arrestins (excluding the single sequence lamprey Arr-VY), corresponding to the collapsed tree shown in Fig. 3B.

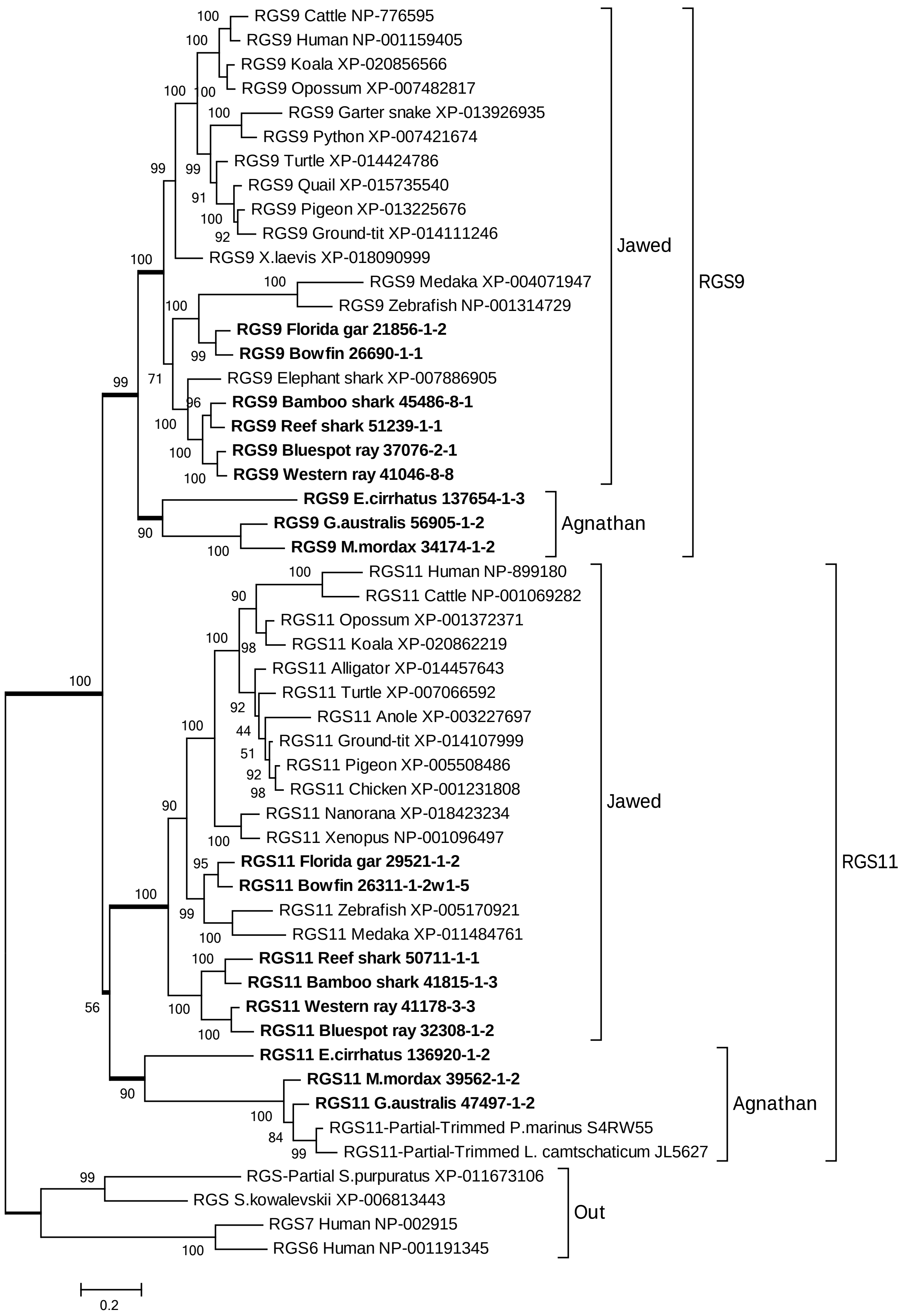

Supplementary Figure S4. Unconstrained molecular phylogeny for vertebrate RGS9 and RGS11, corresponding to Fig. 7A.

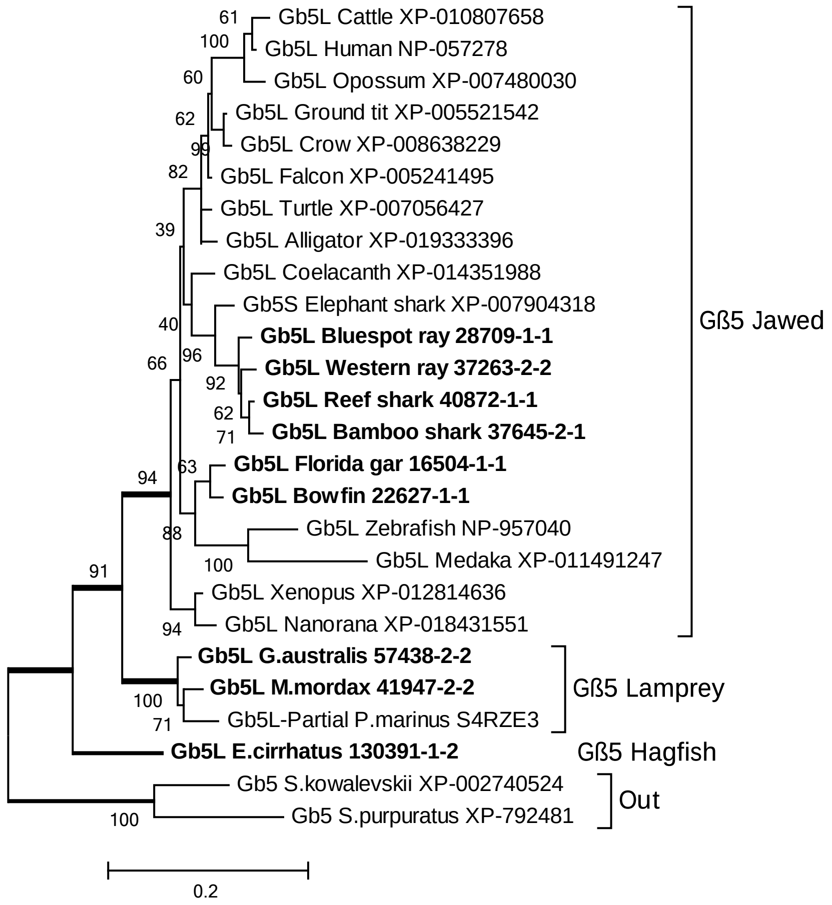

Supplementary Figure S5. Fully expanded molecular phylogeny for jawed and agnathan vertebrate Gβ5 subunits, corresponding to the collapsed tree shown in Fig. 8A.

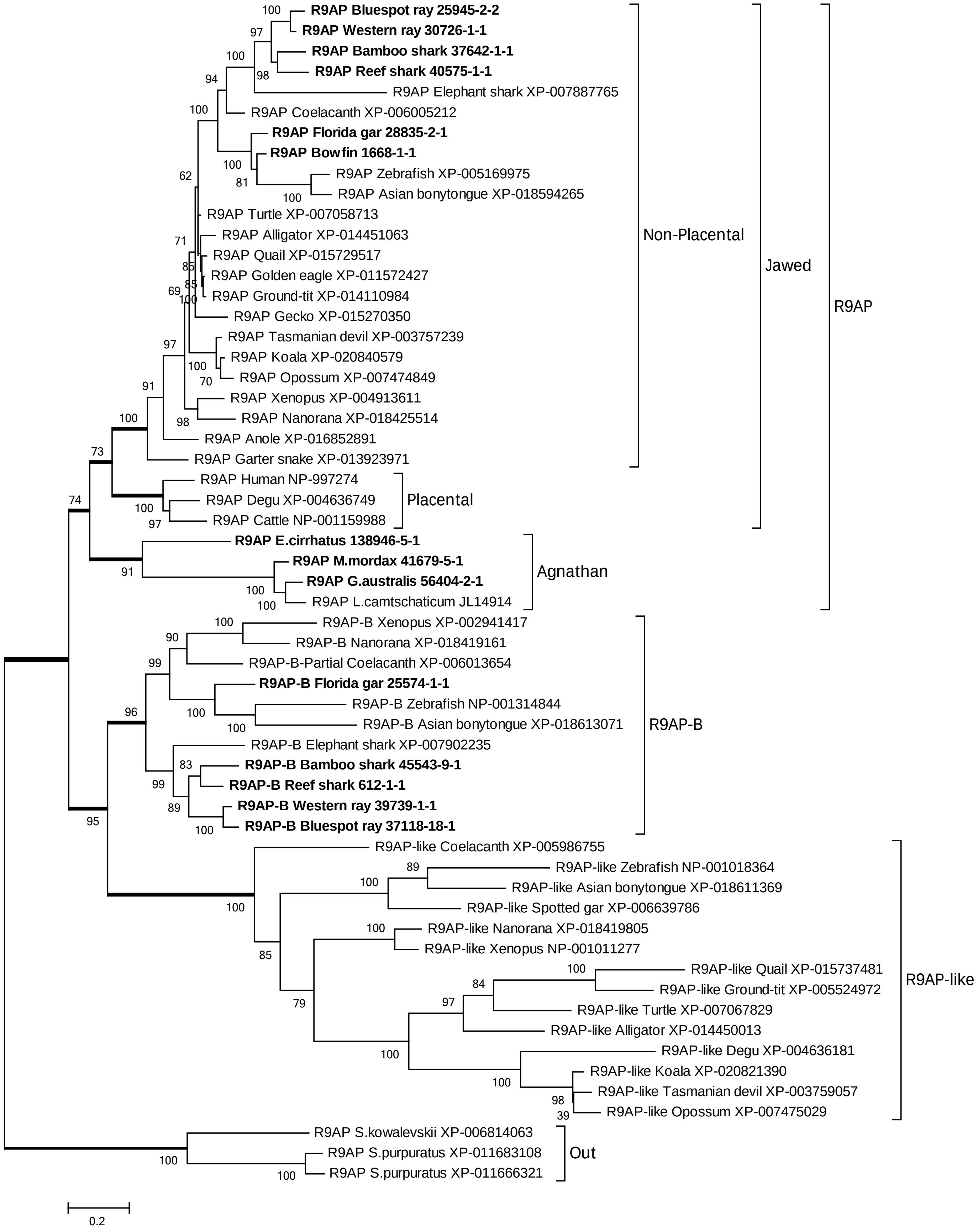

Supplementary Figure S6. Fully expanded molecular phylogeny for R9APs, corresponding to the collapsed tree shown in Fig. 9A.

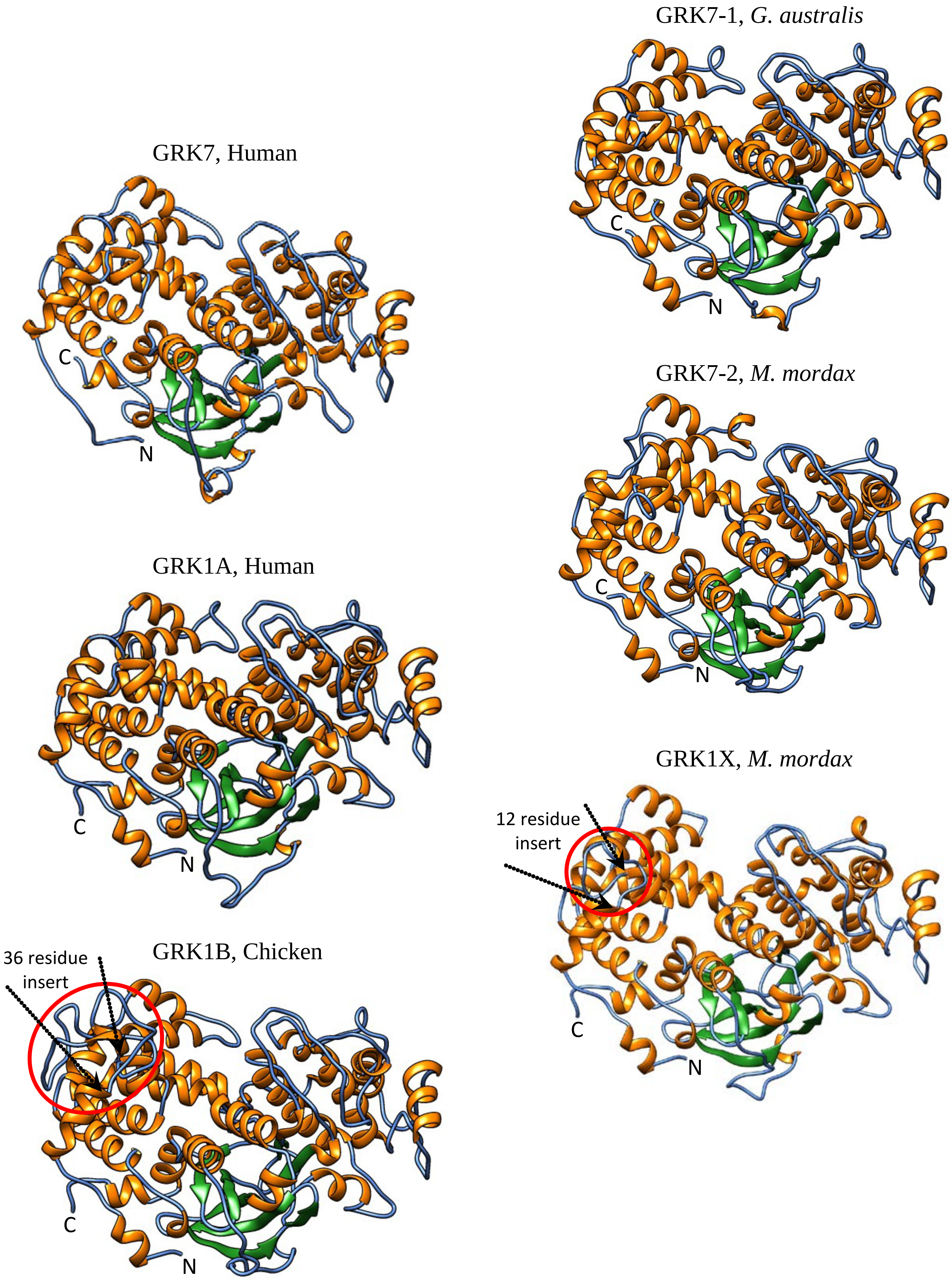

Supplementary Figure S7. Molecular models of GRK isoforms. The structures predicted by SWISS-MODEL (see MATERIALS AND METHODS) for the six isoforms are shown. A kinase template (Protein Data Base ID 4PNI) was used as template for the modelling. Colours identify structural features: Blue, coil; orange, helix; green, strand. N- and C-termini are indicated.

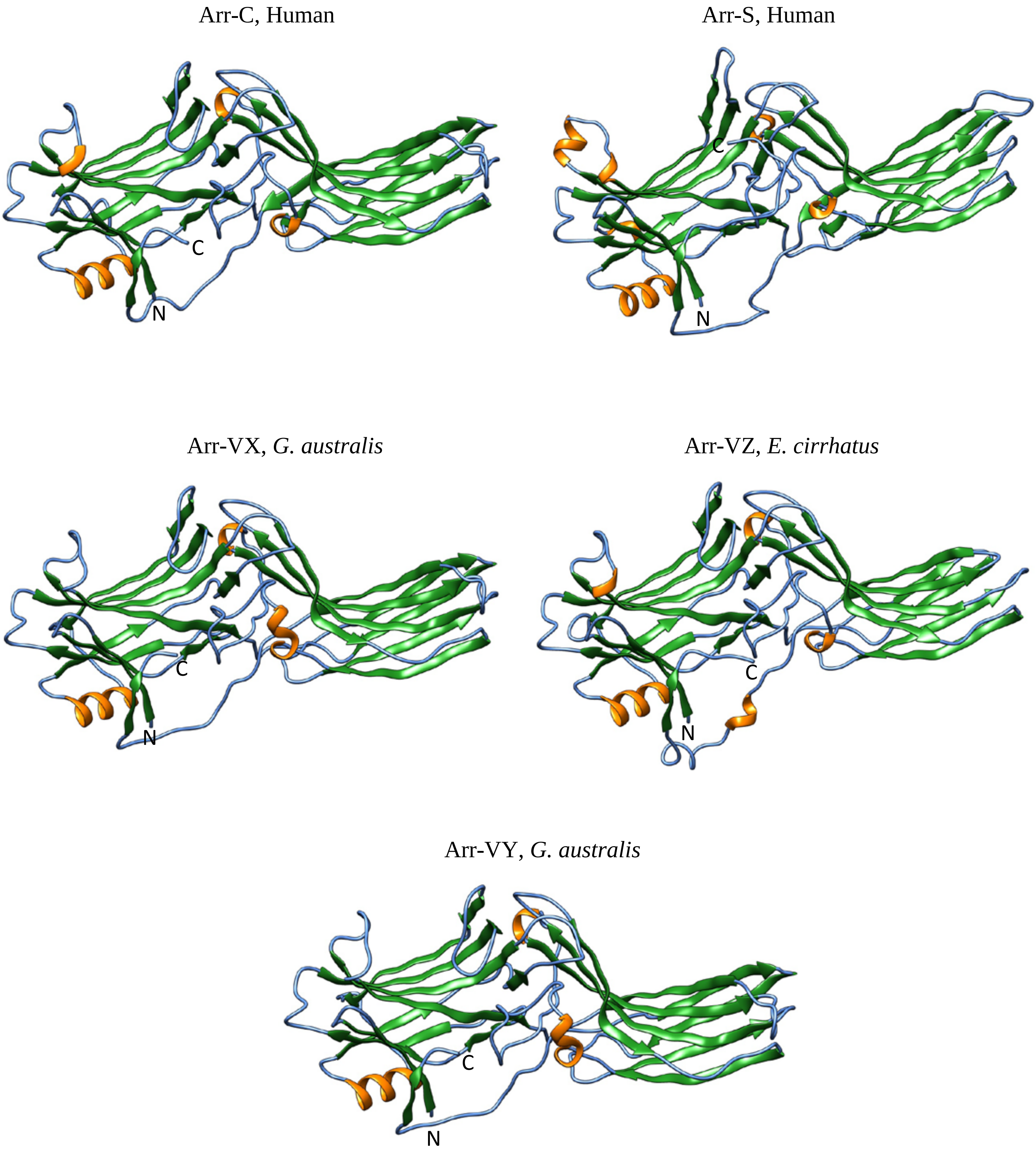

Supplementary Figure S8. Molecular models of arrestin isoforms. The structures predicted by SWISS-MODEL (see Materials and Methods) for jawed and agnathan vertebrate isoforms are shown. An arrestin template (Protein Data Base ID 1SUJ) was used as template for the modelling. Colours identify structural features: Blue, coil; orange, helix; green, strand. N- and C-termini are indicated.
