# Supplementary material for: Evolution of the shut-off steps of vertebrate phototransduction
Source: Open Biol. 2018 Jan 10;8(1):170232. doi: 10.1098/rsob.170232 (PMC5795056; doi:10.1098/rsob.170232)
Supplement: Supplementary Tables [file rsob170232supp2.pdf]

Supplementary Tables
for

Evolution of the shut-off steps of vertebrate phototransduction

Trevor D. Lamb[1], Hardip R. Patel[2], Aaron Chuah[3], and David M. Hunt[4,5]

[1]Eccles Institute of Neuroscience, [2]National Centre for Indigenous Genomics, and [3]Genome Discovery Unit, John Curtin School of Medical Research, The Australian National University, ACT 2600, Australia, and [4]The Lions Eye Institute and [5]School of Biological Sciences, The University of Western Australia, WA 6009, Australia.

Contents

		Page
**Supplementary Table S1**.	Summary of transcripts examined.	2
**Supplementary Table S2**.	Multiple sequence alignment for GRKs.	5
**Supplementary Table S3**.	Multiple sequence alignment for arrestins.	11
**Supplementary Table S4**.	Multiple sequence alignment for RGS9/11s.	15
**Supplementary Table S5**.	Multiple sequence alignment for Gβ5s.	21
**Supplementary Table S6**.	Multiple sequence alignment for R9APs.	23

Supplementary Table S1. Summary of transcripts examined

#	Comp.	Label on Figures	Accession number	aa's	Join	M	*	RPKM	RPKM -CDS
1	GRK1X	GRK1X G.australis 57773-1-1	MG063622	571		M	*	18.1	13.2
2	GRK1X	GRK1X M.mordax 42166-5-1	MG063623	569		M	*	37.1	32.5
3	GRK1A	GRK1A Western ray 41513-9-2	MG063624	564		M	*	104.7	107.5
4	GRK1A	[GRK1A Eastern ray 24652-2-1]	MG063625	564		M	*	13.8	13.3
5	GRK1A	GRK1A Bluespot ray 29212-2-1	MG063626	564		M	*	191.0	171.8
6	GRK1A	GRK1A Bamboo shark 8046-1-1	MG063627	565		M	*	270.5	315.7
7	GRK1A	GRK1A Reef shark 53758-1-1	MG063628	565		M	*	354.5	449.6
8	GRK1A	GRK1A Florida gar 24879-1-1	MG063629	563		M	*	248.2	206.8
9	GRK1A	GRK1A Bowfin 29529-1-1	MG063630	563		M	*	264.1	322.3
10	GRK1B	GRK1B Western ray 34447-1-1	MG063631	558		M	*	8.0	8.7
11	GRK1B	[GRK1B-Partial Eastern ray 22403-1-1]	MG063632	500		M		4.4	4.7
12	GRK1B	GRK1B Bluespot ray 4032-1-1	MG063633	558		M	*	37.3	41.0
13	GRK1B	GRK1B Bamboo shark 26651-1-1	MG063634	559		M	*	2.9	2.8
14	GRK1B	GRK1B Bowfin 26676-4-1	MG063635	561		M	*	21.1	21.8
15	GRK7	GRK7 Western ray 39734-1-1	MG063636	554		M	*	160.6	182.3
16	GRK7	[GRK7 Eastern ray 24666-1-1]	MG063637	554		M	*	184.0	58.1
17	GRK7	GRK7 Bluespot ray 37188-7-2	MG063638	554		M	*	62.6	75.3
18	GRK7	GRK7 Bamboo shark 35291-1-1	MG063639	552		M	*	339.8	466.6
19	GRK7	GRK7 Reef shark 15561-1-1	MG063640	555		M	*	384.3	437.0
20	GRK7	GRK7 Florida gar 30401-2-1	MG063641	550		M	*	27.2	27.2
21	GRK7	GRK7 Bowfin 27257-5-2	MG063642	551		M	*	73.6	111.4
22	GRK7-1	GRK7-1 G.australis 58623-2-1w1-2	MG063643	556	J	M	*	8.8	18.4
23	GRK7-1	GRK7-1 M.mordax 40500-6-1	MG063644	553		M	*	25.6	27.7
24	GRK7-2	GRK7-2 E.cirrhatus 127887-1-1	MG063645	549		M	*	40.7	55.0
25	GRK7-2	GRK7-2-Non-contig G.australis 26295-2-1w27699-1-1	MG063646	486	NJ			6.4	6.4
26	Arr-S1	Arr-S1 Western ray 26221-2-1	MG063647	390		M	*	1189.5	2436.4
27	Arr-S1	[Arr-S1 Eastern ray 41685-1-1]	MG063648	390		M	*	2745.4	687.7
28	Arr-S1	Arr-S1 Bluespot ray 30422-1-1	MG063649	390		M	*	3503.3	4155.8
29	Arr-S1	Arr-S1 Bamboo shark 45077-7-1	MG063650	384		M	*	2115.6	2268.8
30	Arr-S1	Arr-S1 Reef shark 55938-25-1	MG063651	383		M	*	2475.3	4997.8
31	Arr-S2	Arr-S2 Western ray 22644-1-1	MG063652	427		M	*	384.5	461.4
32	Arr-S2	[Arr-S2 Eastern ray 20756-1-1]	MG063653	427		M	*	125.2	175.0
33	Arr-S2	Arr-S2 Bluespot ray 31414-1-1	MG063654	420		M	*	746.8	852.2
34	Arr-S2	Arr-S2 Bamboo shark 28962-1-1	MG063655	422		M	*	931.8	1104.3
35	Arr-S2	Arr-S2 Reef shark 55253-10-3	MG063656	422		M	*	228.3	232.1
36	Arr-S	Arr-S Florida gar 22184-1-1	MG063657	391		M	*	1390.9	1199.4
37	Arr-S	Arr-S Bowfin 24232-1-2	MG063658	392		M	*	1527.0	2067.2
38	Arr-VY	Arr-VY E.cirrhatus 129805-1-1	MG063659	385		M	*	431.6	626.4
39	Arr-VY	Arr-VY G.australis 54008-1-2	MG063660	405		M	*	976.0	1170.1
40	Arr-VX	Arr-VX G.australis 59149-4-1	MG063661	392		M	*	988.0	1243.1
41	Arr-VX	Arr-VX M.mordax 42250-9-1	MG063662	391		M	*	921.9	1039.0
42	Arr-C	Arr-C Western ray 37242-1-1	MG063663	394		M	*	145.9	268.3
43	Arr-C	[Arr-C Eastern ray 20877-1-1]	MG063664	394		M	*	319.1	50.9
44	Arr-C	Arr-C Bluespot ray 36257-4-1	MG063665	394		M	*	198.7	205.3
45	Arr-C	Arr-C Bamboo shark 45371-8-2	MG063666	394		M	*	79.7	87.5

46	Arr-C	Arr-C Florida gar 15939-1-1	MG063667	388		M	*	1139.6	1172.2
47	Arr-C	Arr-C Bowfin 14016-1-1	MG063668	386		M	*	2604.9	3140.9
48	Arr-BX	Arr-BX-Partial G.australis 54326-4-1w1-1w2-3	MG063669	332	J		*	18.3	19.4
49	Arr-BX	Arr-BX-Partial M.mordax 41599-4-1	MG063670	243			*	46.7	49.8
50	Arr-BY	Arr-BY-Non-contig G.australis 41931-1-1w37543-2-1w1-1w53942-1-1	MG063671	354	NJ	M	*	5.5	6.1
51	Arr-BY	Arr-BY M.mordax 41599-5-1	MG063672	423		M	*	24.7	26.4
52	Arr-B1	Arr-B1 Florida gar 28267-2-1w1-11	MG063673	418	J	M	*	11.8	7.2
53	Arr-B1	Arr-B1 Bowfin 26818-1-6	MG063674	418		M	*	19.5	12.9
54	RGS9	RGS9 E.cirrhatus 137654-1-3	MG063675	498		M	*	128.9	156.7
55	RGS9	RGS9 G.australis 56905-1-2	MG063676	478		M	*	50.8	56.7
56	RGS9	RGS9 M.mordax 34174-1-2	MG063677	478		M	*	8.8	10.0
57	RGS9	RGS9 Western ray 41046-8-8	MG063678	490		M	*	23.1	25.4
58	RGS9	[RGS9-Non-contig Eastern ray 1867-2-1w1-1]	MG063679	478	NJ	M	*	3.2	3.3
59	RGS9	RGS9 Bluespot ray 37076-2-1	MG063680	490		M	*	83.9	96.6
60	RGS9	RGS9 Bamboo shark 45486-8-1	MG063681	490		M	*	51.1	56.1
61	RGS9	RGS9 Reef shark 51239-1-1	MG063682	491		M	*	70.6	81.9
62	RGS9	RGS9 Florida gar 21856-1-2	MG063683	489		M	*	112.7	85.9
63	RGS9	RGS9 Bowfin 26690-1-1	MG063684	487		M	*	74.5	60.7
64	RGS11	RGS11 E.cirrhatus 136920-1-2	MG063685	433		M	*	5.6	6.1
65	RGS11	RGS11 G.australis 47497-1-2	MG063686	419		M	*	13.0	15.7
66	RGS11	RGS11 M.mordax 39562-1-2	MG063687	413		M	*	2.8	6.2
67	RGS11	RGS11 Western ray 41178-3-3	MG063688	465		M	*	26.4	28.3
68	RGS11	[RGS11-Partial Eastern ray 22212-1-3]	MG063689	391			*	15.9	16.3
69	RGS11	RGS11 Bluespot ray 32308-1-2	MG063690	467		M	*	32.7	29.2
70	RGS11	RGS11 Bamboo shark 41815-1-3	MG063691	465		M	*	10.3	10.2
71	RGS11	RGS11 Reef shark 50711-1-1	MG063692	461			*	9.3	10.9
72	RGS11	RGS11 Florida gar 29521-1-2	MG063693	471		M	*	7.6	5.3
73	RGS11	RGS11 Bowfin 26311-1-2w1-5	MG063694	469	J	M	*	18.9	31.0
74	Gb5L	Gb5L E.cirrhatus 130391-1-2	MG063695	395		M	*	72.0	94.2
75	Gb5L	Gb5L G.australis 57438-2-2	MG063696	395		M	*	37.7	55.9
76	Gb5L	Gb5L M.mordax 41947-2-2	MG063697	395		M	*	30.5	33.2
77	Gb5L	Gb5L Western ray 37263-2-2	MG063698	395		M	*	127.0	137.6
78	Gb5L	[Gb5L Eastern ray 13249-1-1]	MG063699	395		M	*	141.0	17.3
79	Gb5L	Gb5L Bluespot ray 28709-1-1	MG063700	395		M	*	186.6	198.6
80	Gb5L	Gb5L Bamboo shark 37645-2-1	MG063701	395		M	*	90.0	95.6
81	Gb5L	Gb5L Reef shark 40872-1-1	MG063702	395		M	*	221.0	252.4
82	Gb5L	Gb5L Florida gar 16504-1-1	MG063703	395		M	*	102.4	66.4
83	Gb5L	Gb5L Bowfin 22627-1-1	MG063704	395		M	*	41.0	77.3
84	R9AP	R9AP E.cirrhatus 138946-5-1	MG063705	246		M	*	7.7	4.7
85	R9AP	R9AP G.australis 56404-2-1	MG063706	238		M	*	7.9	9.8
86	R9AP	R9AP M.mordax 41679-5-1	MG063707	243		M	*	16.0	12.7
87	R9AP	R9AP Western ray 30726-1-1	MG063708	244		M	*	19.6	30.0
88	R9AP	[R9AP Eastern ray 17388-1-1]	MG063709	244		M	*	25.9	21.9
89	R9AP	R9AP Bluespot ray 25945-2-2	MG063710	238		M	*	41.4	51.9
90	R9AP	R9AP Bamboo shark 37642-1-1	MG063711	239		M	*	8.7	9.7
91	R9AP	R9AP Reef shark 40575-1-1	MG063712	234		M	*	4.8	5.5
92	R9AP	R9AP Florida gar 28835-2-1	MG063713	238		M	*	27.3	52.3
93	R9AP	R9AP Bowfin 1668-1-1	MG063714	238		M	*	36.0	63.4

94	R9AP-B	R9AP-B Western ray 39739-1-1	MG063715	254		M	*	21.0	46.2
95	R9AP-B	[R9AP-B Eastern ray 23740-1-1]	MG063716	254		M	*	31.2	4.0
96	R9AP-B	R9AP-B Bluespot ray 37118-18-1	MG063717	254		M	*	41.7	58.7
97	R9AP-B	R9AP-B Bamboo shark 45543-9-1	MG063718	255		M	*	59.7	112.8
98	R9AP-B	R9AP-B Reef shark 612-1-1	MG063719	254		M	*	140.0	213.4
99	R9AP-B	R9AP-B Florida gar 25574-1-1	MG063720	254		M	*	44.2	41.7

Notes, and explanation of columns

Column 3, ‘Label on Figures’: Sequences are indicated ‘-Partial’ if they included fewer than 95% of the expected residues. Entries in square brackets do not appear in phylogenies because the protein sequence was identical to another sequence included in the tree; for example, all entries for Eastern ray were omitted in favour of the identical protein sequences for Western ray.

Column 6, ‘Join’: An entry ‘J’ indicates that the sequence was manually joined from more than one contig. These joined sequences overlapped, except where indicated ‘NJ’ for a non-contiguous join.

Column 7: An entry ‘M’ indicates that the sequence started with a Met.

Column 8. An entry ‘*’ indicates that sequence ended with a stop codon.

Column 9: RPKM = Reads per kilobase per million mapped reads.

Column 10: RPKM-CDS = Same, but calculated across only the coding region of the transcript.

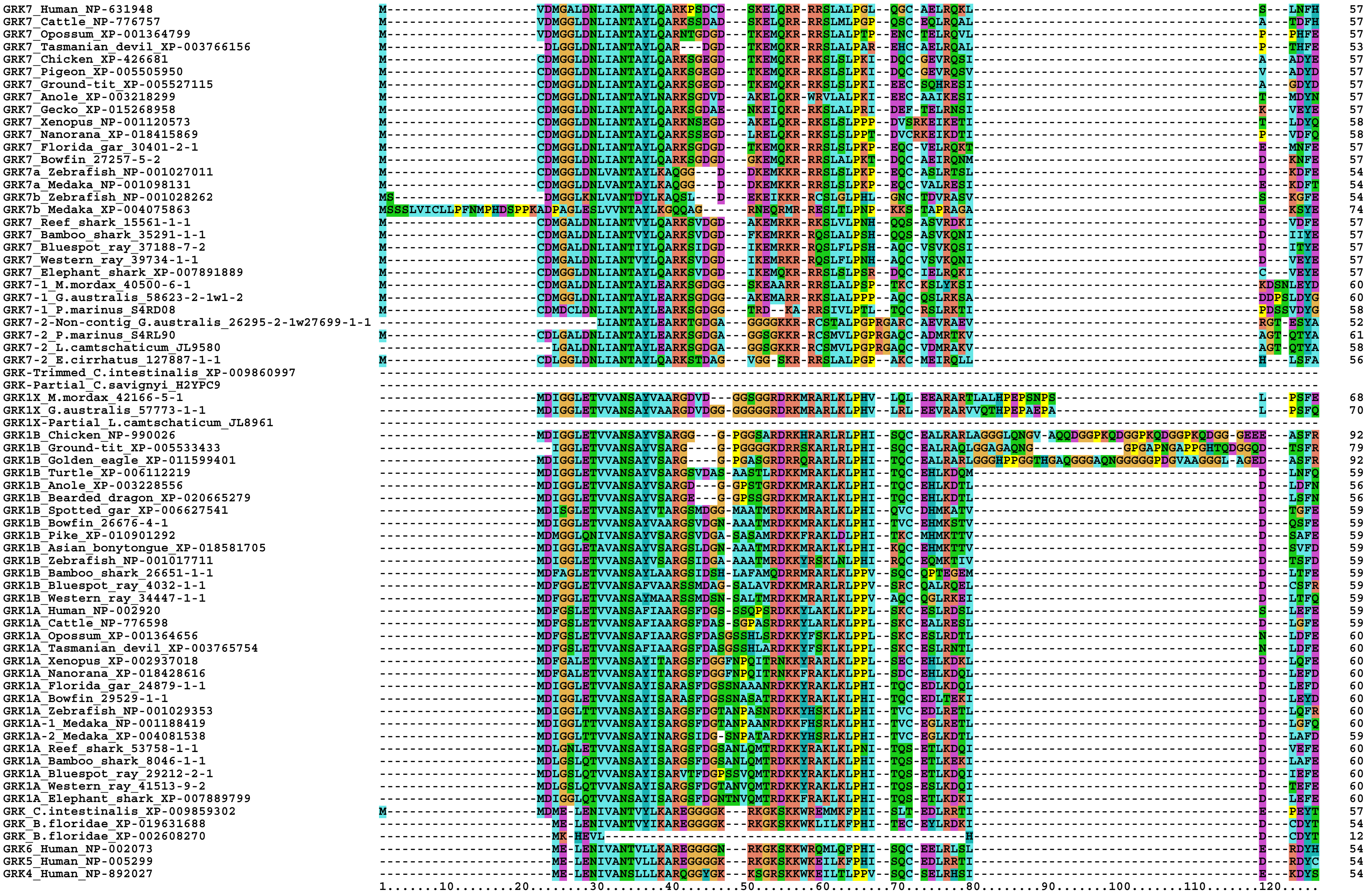
GRK7_Human_NP-631948 57
GRK7_Cattle_NP-776757 57
GRK7_Opossum_XP-001364799 57
GRK7_Tasmanian_devil_XP-003766156 53
GRK7_Chicken_XP-426681 57
GRK7_Pigeon_XP-005505950 57
GRK7_Ground-tit_XP-005527115 57
GRK7_Anole_XP-003218299 57
GRK7_Gecko_XP-015268958 57
GRK7_Xenopus_NP-001120573 58
GRK7_Nanorana_XP-018415869 58
GRK7_Florida_gar_30401-2-1 57
GRK7_Bowfin_27257-5-2 57
GRK7a_Zebrafish_NP-001027011 54
GRK7a_Medaka_NP-001098131 54
GRK7b_Zebrafish_NP-001028262 54
GRK7b_Medaka_XP-004075863 74
GRK7_Reef_shark_15561-1-1 57
GRK7_Bamboo_shark_35291-1-1 57
GRK7_Bluespot_ray_37188-7-2 57
GRK7_Western_ray_39734-1-1 57
GRK7_Elephant_shark_XP-007891889 57
GRK7-1_M.mordax_40500-6-1 60
GRK7-1_G.australis_58623-2-1w1-2 60
GRK7-1_P.marinus_S4RD08 58
GRK7-2-Non-contig_G.australis_26295-2-1w27699-1-1 52
GRK7-2_P.marinus_S4RL90 61
GRK7-2_L.camtschaticum_JL9580 58
GRK7-2_E.cirrhatus_127887-1-1 56
GRK-Trimmed_C.intestinalis_XP-009860997
GRK-Partial_C.savignyi_H2YPC9
GRK1X_M.mordax_42166-5-1 68
GRK1X_G.australis_57773-1-1 70
GRK1X-Partial_L.camtschaticum_JL8961
GRK1B_Chicken_NP-990026 92
GRK1B_Ground-tit_XP-005533433 79
GRK1B_Golden_eagle_XP-011599401 92
GRK1B_Turtle_XP-006112219 59
GRK1B_Anole_XP-003228556 56
GRK1B_Bearded_dragon_XP-020665279 56
GRK1B_Spotted_gar_XP-006627541 59
GRK1B_Bowfin_26676-4-1 59
GRK1B_Pike_XP-010901292 59
GRK1B_Asian_bonytongue_XP-018581705 59
GRK1B_Zebrafish_NP-001017711 59
GRK1B_Bamboo_shark_26651-1-1 59
GRK1B_Bluespot_ray_4032-1-1 59
GRK1B_Western_ray_34447-1-1 59
GRK1A_Human_NP-002920 59
GRK1A_Cattle_NP-776598 59
GRK1A_Opossum_XP-001364656 60
GRK1A_Tasmanian_devil_XP-003765754 60
GRK1A_Xenopus_XP-002937018 60
GRK1A_Nanorana_XP-018428616 60
GRK1A_Florida_gar_24879-1-1 60
GRK1A_Bowfin_29529-1-1 60
GRK1A_Zebrafish_NP-001029353 60
GRK1A-1_Medaka_NP-001188419 60
GRK1A-2_Medaka_XP-004081538 59
GRK1A_Reef_shark_53758-1-1 60
GRK1A_Bamboo_shark_8046-1-1 60
GRK1A_Bluespot_ray_29212-2-1 60
GRK1A_Western_ray_41513-9-2 60
GRK1A_Elephant_shark_XP-007889799 60
GRK_C.intestinalis_XP-009859302 57
GRK_B.floridae_XP-019631688 54
GRK_B.floridae_XP-002608270 12
GRK6_Human_NP-002073 54
GRK5_Human_NP-005299 54
GRK4_Human_NP-892027 54
1.......10........20........30........40........50........60........70........80........90.......100.......110.......120.....

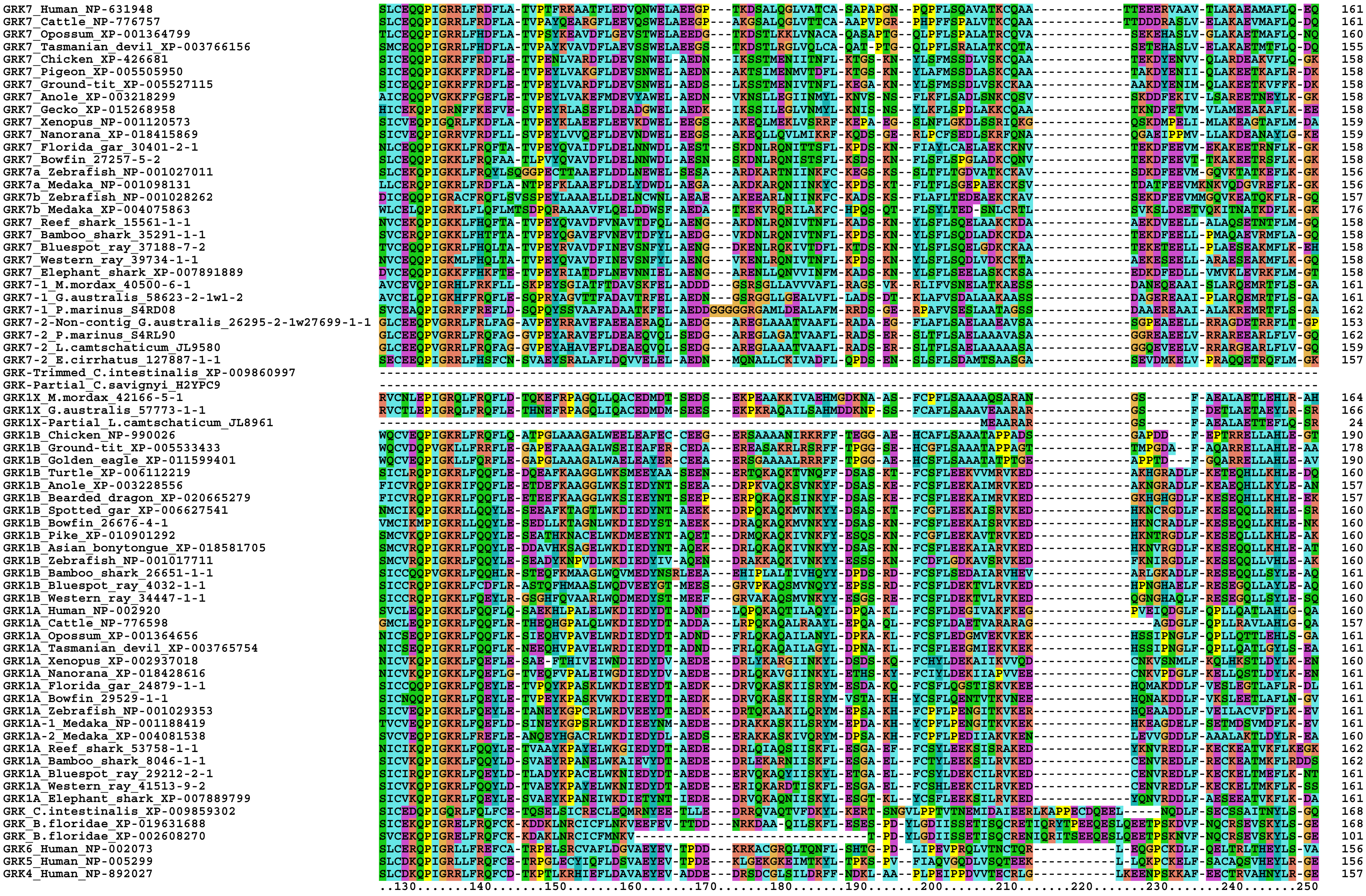
GRK7_Human_NP-631948 161
GRK7_Cattle_NP-776757 161
GRK7_Opossum_XP-001364799 160
GRK7_Tasmanian_devil_XP-003766156 155
GRK7_Chicken_XP-426681 158
GRK7_Pigeon_XP-005505950 158
GRK7_Ground-tit_XP-005527115 158
GRK7_Anole_XP-003218299 158
GRK7_Gecko_XP-015268958 158
GRK7_Xenopus_NP-001120573 159
GRK7_Nanorana_XP-018415869 159
GRK7_Florida_gar_30401-2-1 158
GRK7_Bowfin_27257-5-2 158
GRK7a_Zebrafish_NP-001027011 156
GRK7a_Medaka_NP-001098131 156
GRK7b_Zebrafish_NP-001028262 157
GRK7b_Medaka_XP-004075863 176
GRK7_Reef_shark_15561-1-1 158
GRK7_Bamboo_shark_35291-1-1 158
GRK7_Bluespot_ray_37188-7-2 158
GRK7_Western_ray_39734-1-1 158
GRK7_Elephant_shark_XP-007891889 158
GRK7-1_M.mordax_40500-6-1 161
GRK7-1_G.australis_58623-2-1w1-2 161
GRK7-1_P.marinus_S4RD08 162
GRK7-2-Non-contig_G.australis_26295-2-1w27699-1-1 153
GRK7-2_P.marinus_S4RL90 162
GRK7-2_L.camtschaticum_JL9580 159
GRK7-2_E.cirrhatus_127887-1-1 157
GRK-Trimmed_C.intestinalis_XP-009860997
GRK-Partial_C.savignyi_H2YPC9
GRK1X_M.mordax_42166-5-1 164
GRK1X_G.australis_57773-1-1 166
GRK1X-Partial_L.camtschaticum_JL8961 24
GRK1B_Chicken_NP-990026 190
GRK1B_Ground-tit_XP-005533433 178
GRK1B_Golden_eagle_XP-011599401 190
GRK1B_Turtle_XP-006112219 160
GRK1B_Anole_XP-003228556 157
GRK1B_Bearded_dragon_XP-020665279 157
GRK1B_Spotted_gar_XP-006627541 160
GRK1B_Bowfin_26676-4-1 160
GRK1B_Pike_XP-010901292 160
GRK1B_Asian_bonytongue_XP-018581705 160
GRK1B_Zebrafish_NP-001017711 160
GRK1B_Bamboo_shark_26651-1-1 161
GRK1B_Bluespot_ray_4032-1-1 160
GRK1B_Western_ray_34447-1-1 160
GRK1A_Human_NP-002920 160
GRK1A_Cattle_NP-776598 157
GRK1A_Opossum_XP-001364656 161
GRK1A_Tasmanian_devil_XP-003765754 161
GRK1A_Xenopus_XP-002937018 160
GRK1A_Nanorana_XP-018428616 161
GRK1A_Florida_gar_24879-1-1 161
GRK1A_Bowfin_29529-1-1 161
GRK1A_Zebrafish_NP-001029353 161
GRK1A-1_Medaka_NP-001188419 161
GRK1A-2_Medaka_XP-004081538 160
GRK1A_Reef_shark_53758-1-1 162
GRK1A_Bamboo_shark_8046-1-1 162
GRK1A_Bluespot_ray_29212-2-1 161
GRK1A_Western_ray_41513-9-2 161
GRK1A_Elephant_shark_XP-007889799 161
GRK_C.intestinalis_XP-009859302 168
GRK_B.floridae_XP-019631688 168
GRK_B.floridae_XP-002608270 101
GRK6_Human_NP-002073 156
GRK5_Human_NP-005299 156
GRK4_Human_NP-892027 157
..130.......140.......150.......160.......170.......180.......190.......200.......210.......220.......230.......240.......250

```
                                                                                                   ::* *:: *: :**  .    .  **  *     . ::* *  *::.:  : * : :* .**:::*:.
GRK7_Human_NP-631948                                PFKDFV-TSAFYDKFLQWKLFEMQPVSDKYFTEFRVLGKGGFGE----VCAVQVKNTGKMYACKKLDKKRLKKKGGEKMALLEKEILEKVSSPFIVSLAYAFESKTHLCLVMSLMNGGDLKFHIY  281
GRK7_Cattle_NP-776757                               PFREFL-ASPFYDKFLQWKVFEMQPVSDKYFEEFRVLGKGGFGE----VCAVQVKNTGKMYACKKLDKKRLKKKNGEKMALLEKEILERVSSPFIVSLAYAFESKSHLCLVMSLMNGGDLKFHIY  281
GRK7_Opossum_XP-001364799                           PFQDFQ-ASPFFDKFLQWKAFEQQPITEKYFYEFRVLGKGGFGE----VCAVQVKNTGKMYACKKLDKKRLKKKRGEQMALLEKEILEKVNSPFIVTLAYAFESKSHLCLVMSLMNGGDLKFHIY  280
GRK7_Tasmanian_devil_XP-003766156                   PFQDFQ-ASPFFDRFLQWKAFEQQPITEKYFEEFRVLGKGGFGE----VCAVQVKNTGKMYACKKLDKKRLKKKRGEQMALLEKEILEKVNSPFIVTLAYAFESKSHLCLVMSLMNGGDLKYHIY  275
GRK7_Chicken_XP-426681                              PFQDFQ-TSPFYDKFLQWKVFEKQPVTEKYFYEFRVLGKGGFGE----VCAIQVKNTGKMYACKKLDKKRLKKKCGEKMALLEKEILEKVNSPFIVTLAYAYESKSHLCLVMSLMNGGDLKYHIY  278
GRK7_Pigeon_XP-005505950                            PFQDFQ-TSPLFDKFIQWKVFEKQPVTEKYFYEFRVLGKGGFGE----VCAIQVKNTGKMYACKKLDKKRLKKKGGEKMALLEKEILEKVNSPFIVTLAYAYESKSHLCLVMSLMNGGDLKYHIY  278
GRK7_Ground-tit_XP-005527115                        PFQDFQ-NSPFYDKFIQWKVFEKQPVTEKYFSEFRVLGKGGFGE----VCAIQVKNTGKMYACKKLDKKRLKKKSGEKMALLEKEILEKVNSPFIVTLAYAYETKTHLCLVMSLMNGGDLKYHIY  278
GRK7_Anole_XP-003218299                             PFQDFQ-TSPFFDKFVQWKGFEKQPVNEKLFDEFRVLGKGGFGE----VCAIQARNTGKMYACKKLDKKRLKKKNGEKMALLEKEILEKVNSRFIVTLSYAYQSKAALCLVMSLMNGGDLKYHIY  278
GRK7_Gecko_XP-015268958                             PFEEFQ-KSQYFEKFLQWKAFEKQPVSERHFEEFRVLGKGGFGE----VCAVQVKNTGKMYACKKLDKKRLKKKNGEKMALLEKQILERVNSRFIVSLAYAYQTKTDLCLVMSLMNGGDLRFHIY  278
GRK7_Xenopus_NP-001120573                           PFQDYQ-NSSFYDRFLQWKAFERQQITQKYFYEFRILGKGGFGE----VCAIQVKNTGQMYACKKLDKKRLKKKNGEKMALLEKEILEKVHSPFIVSLAYAYETKSHLCLVMSLMNGGDLKFHIY  279
GRK7_Nanorana_XP-018415869                          PFEAYQ-NSDYYDKFLQWKAFERQPITHKFFYEFRVLGKGGFGE----VCAIQVKNTGQMYACKKLDKKRLKKKNGEKMALLEKEILEKVHSPFIVSLAYAYESKTHLCLVMSLMNGGDLKYHIY  279
GRK7_Florida_gar_30401-2-1                          AFDEYQ-NSPFFERFLRWKVFERQPITEKYFYEFRILGKGGFGE----VCAIQVKVSGQMYACKKLDKKRLKKKGEKMALLEKEILEKVNSLFIVKLAYAYETKSHLCLVMSLMNGGDLKYHIY  278
GRK7_Bowfin_27257-5-2                               AFEEYQ-NSFFFEKFLQWKVFERQPITEKYFYEFRILGKGGFGE----VCAIQVKNTGQMYACKKLDKKRLKKKGGEKMALLEKEILEKVNSPFIVKLAYAYESKSHLCLVISLMNGGDLKYHIY  278
GRK7a_Zebrafish_NP-001027011                        PFTEYQ-ASPFFDKFLQWKEYEKQPISEKYFYEFRTLGKGGFGE----VCAVQVKNTGQMYACKKLCKKRLKKHGEKMALLEKKILERVNSLFIVSLAYAYDTKTHLCLVMSLMNGGDLKYHIY  276
GRK7a_Medaka_NP-001098131                           PFTEYQ-GSQYFDKFLQWKEYEKQPISDKYFYEFRTLGKGGFGE----VCAVQVKNTGQMYACKKLCKKRLKKGGEKMALLEKQILEKVNSLFLVNLAYAYDTKTHLCLVMTLMNGGDLKYHIY  276
GRK7b_Zebrafish_NP-001028262                        PFEEYQ-TSLFFDRFVQWKKFEKQPITDKYFYEFRTLGKGGFGE----VCGVQVKTTGQMYACKKLDKKRLKKKSGEKMALLEKEILEMVNSLFIVNLAYAFDTKTHLCLVMTLMSGGDLKYHIF  277
GRK7b_Medaka_XP-004075863                           PFLEYL-KSPHFYRFLQWKECERQKITDKYFYEFRTLGRGGFGE----VCVVQVKYTGHMYACKKLDKRRLKKKSGERLALQEKQILEKVNSPFIVNLAYAYESRTHLCLVMDLMSGGDLKFHIY  296
GRK7_Reef_shark_15561-1-1                           PFQDFQ-NSPFFSKFLQWKTFERQPITEKYFYQFRVLGKGGFGE----VCAIQAKTSGKMYACKQLDKKRLKKQGHEMALLEKEILEQTNSRFIVNLAYAYQSKDRLCLVMTLMNGGDLKFHIY  278
GRK7_Bamboo_shark_35291-1-1                         PFQDFQ-NSPFFTKFLQWKTFERQPITEKYFYQFRVLGKGGFGE----VCAIQVKTTGKMYACKQLDKKRLKKHGEEMALLEKEILEKANSPFIVSLAYAYETKNYLCLVMTLMNGGDLKFHIY  278
GRK7_Bluespot_ray_37188-7-2                         AFQDFL-NSPFFEMFLQWKTFERQPITEKYFYQFRILGKGGFGE----VCAMQVKITGKMYACKQLDKKRLKKHGEEMALLEKEILETVNSRFIVNLAYAYETKTHLCLVMTLMNGGDLKFHIY  278
GRK7_Western_ray_39734-1-1                          AFRDFL-NSPFFDKFLQWKTLERQPITEKYFYQFRVLGKGGFGE----VCAIQVKVTGKMYACKQLDKKRLKKHGEEMALLEKEILEKANSRFIVNLAYAYETKTHLCLVMTLMNGGDLKFHIY  278
GRK7_Elephant_shark_XP-007891889                    PFQDFQ-NSPFFDKYTQWKVFERQPINEKYFYEFRVLGKGGFGE----VCAIQVKISGKMYACKKLDKKRLKKKNGEKMTLLEKEILEKTNSRFIVNLSYAFETKSHVCLVMTLMNGGDLKFHIY  278
GRK7-1_M.mordax_40500-6-1                           VFHEFL-QSAFYEKFLQWKMLEKKPVTEKTFYEFRTLGKGGFGE----VCAVQAKVSGKMYACKKLDKKRLKKKGEKLVLVEKEVLEIVNSPFVVQLAYAYETKTHLCLTMSLMIGGDLRYHIY  281
GRK7-1_G.australis_58623-2-1w1-2                    AFREFI-DSASYEKFLQWKMLEKKPVTDKTFYEFRMLGKGGFGE----VCAVQAKVSGKMYACKKLEKKRLKKKNGEKMALLEKEVLEIVSSPFVVQLAYAYETKTHLCLTMSLMNGGDLKYHIY  281
GRK7-1_P.marinus_S4RD08                             PFRG-D-NFRYYRQCLAVKTDRRTEVMQKYFHPHLKGSKTPHSECTCFVCAMQAKMSGKMYACKKLDKKRLKKKNGEKMALLEKELLEMVNSAFVVQLAYAYETKTHLCLTMSLMNGGDLKYHIY  285
GRK7-2-Non-contig_G.australis_26295-2-1w27699-1-1   PFQEFL-ASPFFEKLLQWKVLERQPVNERSFYEFRTLGKGGFGE----VCAIQSKLSGKMYACKKLDKKRLKKKRGEQMALLEKEILESVNSHFVVRLAYAYQTKTHLCLVMSLMNGGDLKYHIY  273
GRK7-2_P.marinus_S4RL90                             PFREFL-ASPFFDRLLQWKVLERQPVSERSFYEFRTLGKGGFGE----VCAIQGKLSGKMYACKKLDKKRLKKKRGEQMALLEKEILESVNSPFVVRLAYAYQTKTHLCLVMSLMNGGDLKYHIY  282
GRK7-2_L.camtschaticum_JL9580                       PFQEFL-ASPFFDRLLQWKVLERQPVNERSFYEFRTLGKGGFGE----VCAIQGKLSGKMYACKKLDKKRLKKKHGEQMALLEKEILESVNSPFVVRLAYAYQTKTHLCLVMSLMNGGDLKYHIY  279
GRK7-2_E.cirrhatus_127887-1-1                       PFREYL-ESPFFQRFLQWKALEQQKITEKHFYEFRVLGKGGFGE----VCAIQVKLTRKMHACKKLNKKSLKKKGQKMALLEKEILESVNSSMVVRLAYAFETKEHLCLVMTLMNGGDLKFHIY  277
GRK-Trimmed_C.intestinalis_XP-009860997             ------------------------------------LGKGGFGE----VYAYQVKVTGKMYACKKIDKKRMKKMHAQGVVMSEKITLEAVNSPFVVSLTYAYENKNHLNLVMTLMGAGDLRFHIY  85
GRK-Partial_C.savignyi_H2YPC9                       ----------------------------------------------------------MYACKKIDKKKMKKQHAESVVMSEKITLEAVNSPFVVSLTYAYENKNQLNLVMTLMGAGDLRFHIY  66
GRK1X_M.mordax_42166-5-1                            ALAKYV-GSPHYLRYLQFKWLEMQPMGEEWFSDFRVLGKGGFGE----VCACQAKATGKLYANKKLNKKRLKKRGGYEGAMVEKLILEKVHSRFIVSLSYAFQTKQELCLVMTIMNGGDLRFHIY  284
GRK1X_G.australis_57773-1-1                         SLAEYA-ESPRYLRYLQFKWLEMQPMGEEWFSDFRVLGKGGFGE----VCACQAKATGKLYANKKLNKKRLKKRGGYEGAIVEKVILEKVHSRFIVSLSYAFQSKQELCLVMTIMNGGDLRFHIY  286
GRK1X-Partial_L.camtschaticum_JL8961                SLAEYV-ESPRYLRYLQFKWLEMQPMGEEWFSDFRVLGKGGFGE----VCACQAKATGKLYANKKLNKKRLKKRGGYEGAIVEKVILEKVHSRFIVSLSYAFQSKLELCLVMTIMNGGDLRFHIY  144
GRK1B_Chicken_NP-990026                             AWAPYL-SSPYFGRFTQFKWLEGQPVGADAFADFRVLGKGGFGK----VCACQRRATGKMYANKKLNKKRLKKRQGYEAAMVEKRILARVHSRFIVSLACAFQTKTDLCLVMTLMNGGDLRYHVY  310
GRK1B_Ground-tit_XP-005533433                       AWAPYR-ASPEFSRFVQFKWLEGQAVNADAFAEFRVLGKGGFGE----VCACQRRATGKMYANKRLNKKRLKKRKGYEAALVEKRILARVHSRFIVSLACAFQTKTDLCLVMTIMNGGDLRYHIY  298
GRK1B_Golden_eagle_XP-011599401                     AWTPYR-ASPEFGRFVQFKWLEGQTVSAEAFVDFRVLGKGGFGK----VCACQRRTTGKMYANKRLNKKRLKKRQGYEAALVEKRILARVHSRFIVSLACAFQTKTDLCLVMTLMNGGDLRYHVY  310
GRK1B_Turtle_XP-006112219                           ALAKYK-DSLFFARFLQFKWLEGQSVTEEWFLDFRVLGKGGFGE----VCACQMRATGKMYANKKLNKKRLKKRNGYEGAIVEKRILAKVHSRFIVTLAYAFQTKVDLCLVMTLMNGGDLRYHVY  280
GRK1B_Anole_XP-003228556                            TMAKYK-ESLHFGRFLQFKWLESQSVTDEWFMDFRVLGKGGFGE----VCACQMKATGKMYANKRLNKKRLKKRNGYEGAIVEKRILAKVHSRFIVTLAYAFQTKLDLCLVMTLMNGGDLRYHVY  277
GRK1B_Bearded_dragon_XP-020665279                   ALGKYK-ESLHFYRFLQFKWLESQSVTDEWFMDFRVLGKGGFGE----VCACQMKATGKMYANKRLNKKRLKKRKGYEGAIVEKRILAKVHSRFIVTLAYAFQTKTDLCLVMTLMNGGDLRYHVY  277
GRK1B_Spotted_gar_XP-006627541                      ALEGFR-GSLYFLRFVQFKWLEAQAVTEEWFMDFRVLGKGGFGE----VHACQMKATGKMYANKKLNKKRLKKRKGYEGAIVEKRILAKVHSRFIVTLAYAFQTKTDLCLVMTIMNGGDLRYHIY  280
GRK1B_Bowfin_26676-4-1                              ALEGFK-GSLYFLRYLQFKWLEAQSVTEEWFMDFRVLGKGGFGE----VHACQMKATGKMYANKKLNKKRLKKRKGYEGAIVEKRILAKVHSRFIVTLAYAFQTKNDLCLVMTIMNGGDLRFHVY  280
GRK1B_Pike_XP-010901292                             ALEGFK-NSMYFLRYVQFKWLEAQPIDEEWFMDFRVLGKGGFGE----VHACQMKATGKLYANKKLNKKRLKKRKGYQGAIVEKRILAKVHSRFIVSLAYAFQTKTNLCLVMTIMNGGDLRFHMY  280
GRK1B_Asian_bonytongue_XP-018581705                 AVQGFK-DSIFFLRYVQFKWLESQSVTEEWFMDFRVLGKGGFGE----VHACQMKATGKMYANKKLNKKRLKKRKGYDGAMVEKRILAKVHSRFIVTLAYAFQTKMDLCLVMTIMNGGDLRYHIY  280
GRK1B_Zebrafish_NP-001017711                        ALTGFK-DSMYFLRYVQFKWLESQPVAEDWFMDFRVLGKGGFGE----VHACQMKATGKMYANKKLNKKRLKKRKGYDGAIIEKRILAKVHSRFIVTLAYAFQTKTDLCLVMTIMNGGDLRFHMY  280
GRK1B_Bamboo_shark_26651-1-1                        AFEGYK-TSQFFQRFLQFKGLEGQPVGDEWFADLRVLGKGGFGE----VCACQMRATGKLYANKKLNKKRLKKRNGHEAVKIEKQILAKVHSRFIVTLAYAFQTKHELCLVMTLMNGGDLRYHIY  281
GRK1B_Bluespot_ray_4032-1-1                         AAESYK-ESTFFLRFLQFKWLEAQPIGEEWFTDFRVLGKGGFGE----VCACQMRATGKMYANKKLNKKRLKKRKGYEATMLEKQILAKVHNRFIVSLAYAFQTKYELCLVMTLMNGGDLRFHIY  280
GRK1B_Western_ray_34447-1-1                         ATEGYK-ASAFFLRFLQFKWLEAQPVSEEWFMDFRVLGKGGFGE----VCACQMRATGKMYANKKLNKKRLKKRKGYEAAIVEKQSLAKVHNRFIVSLAYAFQTKYELCLVMTLMNGGDLRFHIY  280
GRK1A_Human_NP-002920                               PFQEYL-GSLYFLRFLQWKWLEAQPMGEDWFLDFRVLGKGGFGE----VSACQMKATGKLYACKKLNKKRLKKRKGYQGAMVEKKILMKVHSRFIVSLAYAFETKADLCLVMTIMNGGDIRYHIY  280
GRK1A_Cattle_NP-776598                              PFQEFL-DSLYFLRFLQWKWLEAQPMGEDWFLDFRVLGRGGFGE----VFACQMKATGKLYACKKLNKKRLKKRKGYQGAMVEKKILAKVHSRFIVSLAYAFETKTDLCLVMTIMNGGDTRYHIY  277
GRK1A_Opossum_XP-001364656                          PFKEYL-ESLHFLRFLQWKWLEAQPIGEDWFLDFRVLGKGGFGE----VSGCQMKATGKMYACKKLNKKRLKKRKGYQGAMVEKKILEKVHSRFIVSLAYAFETKTDLCLVMTIMNGGDLRYHIY  281
GRK1A_Tasmanian_devil_XP-003765754                  PFREYL-ESLYFMRFLQWKWLEAQPIGEDWFLDFRVLGKGGFGE----VSGCQMKASGKMYACKKLNKKRLKKRKGYQGAIVEKRILSKVHSRFIVSLAYAFETKTDLCLVMTIMNGGDLRYHIY  281
GRK1A_Xenopus_XP-002937018                          AFAQYK-ESKYFSKFLQWKKLEAQPIGDDWFMDFRILGKGGFGE----VSATQMRATGKMYACKKLSKKRLKKRKGFEGAMVEKRILAKVHSRFIVSLAYAFQTKNDVCLVMTIMNGGDLRFHIY  280
GRK1A_Nanorana_XP-018428616                         AFSQYK-EGKIFSRFLQWKWLEAQPVGEDWFMDFRILGKGGFGE----VSASQMRATGKMYACKKLNKKRLKKRKGFDGAMVEKRILAKVHSRFIVSLAYAYQTKNEVCLIMTIMNGGDLRFHIY  281
GRK1A_Florida_gar_24879-1-1                         PFTNYL-ETMYLKRFLQWKWLEAQPIGEDWFLDFRILGKGGFGE----VSATQMRATGKLYACKKLSKKRLKKRKGYEGAMVEKRILAKVHSRFIVSLAYAFQTKTDLCLVMTIMNGGDLRYHIY  281
GRK1A_Bowfin_29529-1-1                              PFSNYK-ETMYFKRFLQWKWLEAQPIGEDWFLDFRILGKGGFGE----VSATQMRGTGKLYACKKLNKKRLKKRKGYEGAMVEKRILARVHSRFIVSLAYAFQTKTDLCLVMTIMNGGDLRYHIY  281
GRK1A_Zebrafish_NP-001029353                        PFTFYL-ETMYFKRFLQWKWLEMQPVAEDWFLDFRVLGKGGFGE----VSACQMRATGKLYACKKLNKKRLKKRKGYEGAMVEKRILARVHSRFIVSLAYAFQTKTELCLVMTIMNGGDLRYHIY  281
GRK1A-1_Medaka_NP-001188419                         PYTFFL-ESMYLKRFLQWKWLEMQPIGEDWFLDFRVLGKGGFGE----VSACQMRATGKLYACKKLNKKRLKKRKGYEGAMVEKRILARVHSRFIVSLAYAFQSKTELCLVMTIMNGGDLRYHIY  281
GRK1A-2_Medaka_XP-004081538                         PYVFFL-ESMYLKRFLQWKWLEMQPMDADWFLDFRVLGKGGFGE----VSACQMKATGKLYACKKLNKKRLKKRKGFEGAMVEKIILEKVHSRFIVSLAYAFQTKEELCLVMTIMNGGDLRYHIY  280
GRK1A_Reef_shark_53758-1-1                          AFEHYK-ESMHFMRFLQWKWLEAQPVGEDWFLDFRVLGKGGFGE----VSACQMKATGKLYACKKLNKKRLKKRKGKQGAIVEKRILARVHSRFIVSLAYAFQTKADLCLVMTIMNGGDLRYHIY  282
GRK1A_Bamboo_shark_8046-1-1                         PFELYK-ESMYFMRFLQWKWLEAQPIGEDWFLEFRVLGKGGFGE----VSACQMKATGKLYACKKLNKKRLKKRKGNQGAMVEKRILARVHTRFIVSLAYAFQTKAELCLVMTIMNGGDLRYHIY  282
GRK1A_Bluespot_ray_29212-2-1                        PFEAYK-ESMYFMRFLQWKWLEAQPVGEDWFMEFRVLGKGGFGE----VSACQMKATGKLYACKKLNKKRLKKRKGNQGAMVEKRILARVHSRFIVSLAYAFQTKAELCLVMTIMNGGDLRYHIY  281
GRK1A_Western_ray_41513-9-2                         PFEGYK-ESMYFMRFLQWKWLEAQPVGEDWFLEFRVLGKGGFGE----VSACQMKATGKLYACKKLNKKRLKKRKGNQGAMVEKRILARVHSRFIVSLAYAFQTKAELCLVMTIMNGGDLRYHIY  281
GRK1A_Elephant_shark_XP-007889799                   PFENYK-ESMYFDRFLQWKWLEAQPIGEEWFLDFRVLGKGGFGE----VSACQMKATGKLYACKKLNKKRLKKRKGYEGTIVEKRILARVHSRFIVSLAYAFQTKEELCLVMTIMNGGDLRYHIY  281
GRK_C.intestinalis_XP-009859302                     PFKEFC-DSIYFNRFLQWKWLERQPVTKNTFRQYRVLGKGGFGE----VCACQVRSSGKMYACKKLEKKRIKKRKGEAMALNEKQILESVNSRFVVSLGYAYETKDALNLVLTIMNGGDLKFHIH  288
GRK_B.floridae_XP-019631688                         PFENFREDKIYFSRFLQWKYLERQPVTKNTFRQYRVLGKGGFGE----VCACQVRATGKMYACKKLEKKRIKKRKGEAMALNEKTILQKINSRFVVSLAYAYETKDALCLVLTIMNGGDLKFHIH  289
GRK_B.floridae_XP-002608270                         PFENFREDKIYFSRFLQWKYLERQPVTKNTFRQYRVLGKGGFGE----VCACQVRATGKMYACKKLEKKRIKKRKGEAMALNEKTILQKINSRFVVSLAYAYETKDALCLVLTIMNGGDLKFHIH  222
GRK6_Human_NP-002073                                PFADYL-DSIYFNRFLQWKWLERQPVTKNTFRQYRVLGKGGFGE----VCACQVRATGKMYACKRLEKKRIKKRKGESMALNEKQILEKVNSRFVVSLAYAYETKDALCLVLTIMNGGDLKFHIY  276
GRK5_Human_NP-005299                                PFHEYL-DSMFFDRFLQWKWLERQPVTKNTFRQYRVLGKGGFGE----VCACQVRATGKMYACKRLEKKRIKKRKGESMALNEKQILEKVNSQFVVNLAYAYETKDALCLVLTIMNGGDLKFHIY  276
GRK4_Human_NP-892027                                PFEEYQ-ESSYFSQFLQWKWLERQPVTKNTFRHYRVLGKGGFGE----VCACQVRATGKMYACKKLQKKRIKKRKGEAMALNEKRILEKVQSRFVVSLAYAYETKDALCLVLTIMNGGDLKFHIY  277
                                                    .......260.......270.......280.......290.......300.......310.......320.......330.......340.......350.......360.......370.....
```

```
                                                                                                                     :***:**:*:***  *: *::*:**   :          .** .:*.**::     *    *::  *  :.*:  .  **
GRK7_Human_NP-631948                                     NVG------T--RGLDMSRVIFYSAQIACGMLHLHELGIVYRDMKPENVLLDDLGNCRLSDLGLAVEMKGG-KPITQRAGTNGYMAPEILMEKVSYSYPVDWFAMGCSIYEMVAGRTPFK---DY   394
GRK7_Cattle_NP-776757                                    SVG------E--PGLDMSRVIFYSAQITCGVLHLHSLGIVYRDMKPENVLLDDLGNCRLSDLGLAVQIQDG-KPITQRAGTNGYMAPEILMEKASYSYPVDWFAMGCSIYEMVAGRTPFR---DY   394
GRK7_Opossum_XP-001364799                                NVG------E--RGLAINRTIFYSAQITCGILHLHSIGIVYRDMKPENVLLDDHGNCRLSDLGLAVQIKEG-KPITQRAGTHGYMAPEILKEEESYSYPVDWFAMGCSIYEMVAGRTPFK---DY   393
GRK7_Tasmanian_devil_XP-003766156                        HVG------E--RGLEINRIIFYSAQITCGILHLHSIGIVYRDMKPENVLLDDRGNCRLSDLGLAVQIKDG-KSITQRAGTNGYMAPEILKEEESYSYPVDWFAMGCTIYEMVAGRTPFK---DY   388
GRK7_Chicken_XP-426681                                   NVG------Q--RGLELNRVIYYSAQITCGILHLHSIKIIYRDMKPENVLLDDNGNCRLSDLGLAVQVKEG-KSITQRAGTNGYMAPEILKE-ESYSYPVDWFAMGCSIYEMIAGRTPFK---NF   390
GRK7_Pigeon_XP-005505950                                 NVG------E--KGLEMKRVIYYSAQITCGILHLHSIKIVYRDMKPENVLLDDNGNCRLSDLGLAVQVKEG-KSITQRAGTNGYMAPEILKE-EDYSYAVDWFAMGCSIYEMIAARTPFK---DF   390
GRK7_Ground-tit_XP-005527115                             NVG------E--RGLEMKRIIFYSAQITCGILHLHSIKIVYRDMKPENVLLDDNGNCRLSDLGLAVQVKEG-KSITQRAGTNGYMAPEILKE-EDYSYPVDWFAMGCTIYEMVAGRTPFK---DY   390
GRK7_Anole_XP-003218299                                  NVG------E--RGLEMNRVIFYSAQITCGILHLHSIQIVYRDMKPENVLLDDNGHCRLSDLGLAVEVKEG-KEIRQRAGTNGYMAPEILNE-EAYTYPVDWFAMGCSIYEMIAGRTPFK---DF   390
GRK7_Gecko_XP-015268958                                  STG------E--RGLKMDRVIYYSAQMTCGLLHLHSINVVYRDMKPDNVLLDEHGNCRLSDLGLAVKVKDD-KPITQRAGTNGYMAPEILKEDETYSYPADWFSLGCTIYEMVAARTPFR---DH   391
GRK7_Xenopus_NP-001120573                                NVG------E--KGIEMKRVIFYSAQICCGILHLHSLKIVYRDMKPENVLLDENGNCRLSDLGLAVKVKEG-KPITQRAGTNGYMAPEILTDE-DYSYPVDWFAMGCSIYEMVAAHTPFR---DP   391
GRK7_Nanorana_XP-018415869                               NVG------E--KGLEIKRVIFYTAQICCGILHLHSIKILYRDMKPENVLLDDNGNCRLSDLGLAVKVKEG-QPITTRAGTNGYMAPEILKDE-NYSYPVDWFAMGCSVYEMIAAQTPFK---DP   391
GRK7_Florida_gar_30401-2-1                               NIG------E--RGIEMNRAIYYTAQITCGILHLHSINIVYRDMKPENVLLDDNGQCRLSDLGLAVEIKEG-KSINQKAGTNGYMAPEILKE-EDYTYSVDWFALGCSIYEMVAGRTPFK---DF   390
GRK7_Bowfin_27257-5-2                                    NIG------E--RGIEMNRAIYYTAQITCGMLHLHSINIVYRDMKPENVLLDDNGQCRLSDLGLAVEVKEG-KTISQKAGTNGYMAPEILKE-ETYTYSVDWFALGCSIFEMVSGRTPFK---DY   390
GRK7a_Zebrafish_NP-001027011                             NIG------E--KGIEMDRIIYYTAQIATGILHLHDMDIVYRDMKPENVLLDSQGQCRLSDLGLAVEIAVG-KTISQKAGTGAYMAPEILNE-TPYRTSVDWWALGCSIYEMVAGYTPFKGPDAK   391
GRK7a_Medaka_NP-001098131                                NIGYDGKGVD--KGIEMKRIIHYTAQITTGILHLHDMDIIYRDMKPENVLLDSQGQCRLSDLGLAIEIAPG-KTVTQMAGTGAYMAPEILSK-TPYRTSVDWWALGCSIYEMVAGYTPFKGPESK   397
GRK7b_Zebrafish_NP-001028262                             HVG------E--VGIEMERIIHYTAQITSGILHLHSMDIVYRDMKPENVLLDCQGQCRLSDLGLAVELPNG-KTTTQKAGTKGYMAPEILKQ-EPYRTSVDWWALGCSIYEMVAGRVPFR---DH   389
GRK7b_Medaka_XP-004075863                                ELG------E--RGIRMERVVYYTAQIISGILHLHSMDIVYRDMKPENVLLDGKGQCRVSDLGLAVELPNG-KMICQRAGTTGYMAPEVLKQ-EYYRTSVDWWALGCSIYEMVAARLPFR---DF   408
GRK7_Reef_shark_15561-1-1                                NIG------E--KGIEMKRTIFYTAQITCGILHLHSLNIVYRDMKPENVLLDDDGNCRISDLGLAAKIKEG-KGINHKAGTNGYMAPEILKD-EEYTYSVDWFALGCSIYEMIAARTPFK---EH   390
GRK7_Bamboo_shark_35291-1-1                              NIG------E--RGIEMKRTIFYAAQITCGILHLHSIKIVYRDMKPENVLLDDNGNCRLSDLGLAVKVKEG-KSITRIAGTNGYMAPEILKE-ENYKYPVDWFALGCSIYEMIAGRTPFK---DY   390
GRK7_Bluespot_ray_37188-7-2                              NIG------E--KGIEMNRAIFYTAQITCGILHLHAQNIVYRDMKPENVLLDDNGNCRLSDLGLAVRIKEG-KAINHKAGTNGYMAPEILKQ-EKYTYSVDWFALGCSIYEMIAGRTPFK---DY   390
GRK7_Western_ray_39734-1-1                               NIG------E--KGLEMNRAMFYTAQITCGILHLHSQNIVYRDMKPENVLLDDNGNCRLSDLGLAVRIKEG-IAINHKAGTNGYMAPEILKE-EKYTYSVDWFALGCSIYEMIAGRTPFK---DY   390
GRK7_Elephant_shark_XP-007891889                         NIG------D--RGIEMSRVQFYTAQITCGLLHLHSINIVYRDMKPENVLLDENGNCRLSDLGLAVEIING-KKTNQRAGTNGYMAPEILKE-ENYTYSVDWFALGCSIYEMIAGRTPFK---DF   390
GRK7-1_M.mordax_40500-6-1                                QMG------T--PGLEKDRVLFYAAQITCGLQHLHTLRVIYRDMKPENVLLDDAGHCRLSDMGLAVLLKNDSKKTTQRAGTNGYMAPEVINE-ERYTYSCDWFSLGCTIYEMIAGRTPFK---DY   394
GRK7-1_G.australis_58623-2-1w1-2                         EIG------E--PGLETDRVLFYAAQITCGMQHLHALRIVYRDMKPENVLLDDAGHCRLSDLGLAVLLKSDSKKITQRAGTNGYMAPEVINE-EGYTYSCDWFSLGCTIYEMTAGRTPFK---DY   394
GRK7-1_P.marinus_S4RD08                                  EMG------E--LGLEMERVLFYAAQITCGMQHLHALRIIYRDMKPENVLLDEAGHCRLSDLGLAVLLKDDGMKITERAGTNGYMAPEVING-EGYTYSCDWFSLGCTIYEMIAGRTPFK---GH   398
GRK7-2-Non-contig_G.australis_26295-2-1w27699-1-1        EXX------X--XXXXXXXXXXXXXXXXXXXXXXXXXXXXXXVYRDMKPENVLLDERGHCRLSDLGLAVVLGGGQETICQRAGTNGYMSPEVVLE-QPYSFDADWFATGCTVYEMICGRAPFK---DY   386
GRK7-2_P.marinus_S4RL90                                  EMG------E--RGLSPARTLFYAAQITCGLQHLHTARIVYRDMKPENVLLDERGHCRLSDLGLAVVLADGQETIRQRAGTNGYMSPEVVLE-EPYAFDADWFATGCTVYEMICGRAPFK---DY   395
GRK7-2_L.camtschaticum_JL9580                            EMG------E--RGLSPARTLFYAAQITCGLQHLHAARIVYRDMKPENVLLDERGHCRLSDLGLAVVLADGQETIRQRAGTNGYMSPEVVLE-EPYAFDADWFATGCTVYEMICGRAPFK---DY   392
GRK7-2_E.cirrhatus_127887-1-1                            NVG--------KRGLSMDRVLFYTAQITCGLHHLHRLSIIYRDMKPENVLLDERGNCRLSDLGLAVVLRNADDKITQRAGTNGYMSPEVIKE-EPYSYDADWFALGCTIHEMISGNTPFR---NF   390
GRK-Trimmed_C.intestinalis_XP-009860997                  ETNEDG----KKRGLTPERTKFYSAQIILGLDHLHRENILYRDMKPENVLLDDKGNVRLADLGLVCILGEG-EKTKGRAGTIGFMPPEMLQS-KQYSYGVDWFSLGCTIFEMIEARGPFV---S-   200
GRK-Partial_C.savignyi_H2YPC9                            ETNNDG----KKKGLSSERAKFYTAQIILGLDHLHRENILYRDMKPENVLLDDGGNVRLADLGLVCILKDG-EQVKGRAGTIGFMPPEMLQS-KHYSYGADWFSLGCTIFEMVEAKGPFV---S-   181
GRK1X_M.mordax_42166-5-1                                 DVD------EANPGFTEARTLFYSAQIVCGLEHLHQHRIIYRDLKPENVLLDDMGHVRLSDMGLATELQEGEDKTKGYAGTPGFMAPELVKG-ELYDYSVDYFTLGVTIFEMIQAKGPFR---YR   399
GRK1X_G.australis_57773-1-1                              DVD------DANQGFSEARTLFYSAQIVCGLEHLHQHRIVYRDLKPENVLLDDMGHVRLSDLGLAIELKEGQDKTKGYAGTPGFMAPELLKG-EPYDYSVDYFTLGVTIFEMIQAKGPFR---YR   401
GRK1X-Partial_L.camtschaticum_JL8961                     DVD------DANQGFPEARSLFYAAQIVCGLEHLHQHRIVYRDLKPENVLLDDMGHVRLSDLGLAIELKEGQDKTKGYAGTPGFMAPELLKG-EPYDYSVDYFTLGVTIYEMIQAKGPFR---YR   259
GRK1B_Chicken_NP-990026                                  NVD------EENPGFPEPRAVFYTAQILLGLEHLHQHRIVYRDLKPENVLLDDAGHVRLSDMGLAVELKDGQNKTRGYAGTPGFMAPEVLRD-EEYDMAVDYFTLGVTLYEMLDAKGPFR---RR   425
GRK1B_Ground-tit_XP-005533433                            NVD------EENPGFAEPRAVFYTAQILLGLEHLHQHRIVYRDLKPENVLLDDAGHVRLSDMGLAVELKDGESKTRGYAGTPGFMAPELLKN-EDYDWSVDYFTLGVTVYEMLEAKGPFR---RR   413
GRK1B_Golden_eagle_XP-011599401                          NVD------EDNPGFPEPRAVFYTAQILLGLEHLHQHRIVYRDLKPENVLLDDAGHVRLSDMGLAVELKEGQTKTRGYAGTPGFMAPEVLRD-EEYDWAVDYFTLGVTLYEMLEAKGPFR---RR   425
GRK1B_Turtle_XP-006112219                                NVD------EKNPGFPESRAVFYTAQIICGLEHLHQNRIIYRDLKPENVLLDDAGHVRLSDLGLAVELPEGKDKTKGYAGTPGFMAPELLQN-QEYDWSVDYFTLGVTLYEMIEAKGPFR---SR   395
GRK1B_Anole_XP-003228556                                 NVD------EKNPGLSESRTIFYTAQIICGLEHLHQNRIIYRDLKPENVLLDDAGHVRLSDLGLAVELPEGKDKTKGYAGTPGFMAPELLKG-EEYDSSVDYFTLGVTIFEMIAAKGPFR---SR   392
GRK1B_Bearded_dragon_XP-020665279                        NVD------EKNPGFSEARAVFYTAQIICGLEHLHQNRIIYRDLKPENVLLDDAGHVRLSDLGLAVELPDGKDKTKGYAGTPGFMAPELLKG-EEYDSSVDYFTLGVTLYEMVAAKGPFR---SR   392
GRK1B_Spotted_gar_XP-006627541                           NVD------EKNPGLNEERTCFYTAQVICGLEHLHQNRIIYRDLKPENVLLDDAGHVRLSDLGLSVELEEGHDKTKGYAGTPGFMAPELLKN-EEYDYSVDYFTLGVTVFEMIAAKGPFR---SR   395
GRK1B_Bowfin_26676-4-1                                   NVD------EKNPGFNEERTCFYGAQIICGLEHLHQNRIIYRDLKPENVLLDDAGHVRISDLGLAIELKDGLNKTKGYAGTPGFMSPELLKN-EEYDYCVDYFTLGVTLYELVAAKGPFR---SR   395
GRK1B_Pike_XP-010901292                                  NVD------EKNPGFNETRACFYIAQIILGLEHLHQHRIVYRDLKPENVLLDDAGHVRLSDLGLAVELPAGTDKTKGYAGTPGFMAPELIEN-KEYDYTVDYFTLGVTLYEMISAKGPFR---TR   395
GRK1B_Asian_bonytongue_XP-018581705                      NVD------EKNPGFNETRACYYTAQIICGLEHLHQHRIIYRDLKPENVLLDDAGHVRISDLGLAVELKPGKDKTKGYAGTPGFMAPELLQN-KEYDYSVDYFTLGVTLYEMIAAKGPFR---SR   395
GRK1B_Zebrafish_NP-001017711                             NVD------EKNPGFNETRACYYTAQIICGLEHLHQHRIVYRDLKPENVLLDDAGHVRLSDLGLAVELPPGKDKTQGYAGTPGFMAPELLQK-KEYDYTVDYFTLGVTLYEMVAAKGPFR---CR   395
GRK1B_Bamboo_shark_26651-1-1                             NVN------EKMPGFEEARACFYSAQILCGLQHLHQNRIIYRDLKPENVLLDDNGHVRISDLGLATVLKEGCSHTRGYAGTPGFMAPEMLKG-ECYDFCVDYFTLGVTLYEMVTAKGPFR---SR   396
GRK1B_Bluespot_ray_4032-1-1                              NVD------EKNPGFQESRAQFYTAQIICGLEHLHQHRIVYRDLKPENVLLDDGGHVRISDLGLATVLKEDREYTKGYAGTPGFMAPEMLKG-EPYSFSVDYFTLGVTLYEMVAAKGPFR---TR   395
GRK1B_Western_ray_34447-1-1                              SVD------EKNPGFEESRARFYTAQIICGLEHLHQHRIIYRDLKPENVLLDDGGHVRISDLGLATVLKEGREQTKGYAGTPGFMAPEMLKG-EHYSFSVDYFTLGVTLYEMVAAKGPFR---VR   395
GRK1A_Human_NP-002920                                    NVN------EENPGFPEPRALFYTAQIICGLEHLHQRRIVYRDLKPENVLLDNDGNVRISDLGLAVELLDGQSKTKGYAGTPGFMAPELLQG-EEYDFSVDYFALGVTLYEMIAARGPFR---AR   395
GRK1A_Cattle_NP-776598                                   NVD------EDNPGFQEPRAIFYTAQIVSGLEHLHQRNIIYRDLKPENVLLDDDGNVRISDLGLAVELKAGQTKTKGYAGTPGFMAPELLLG-EEYDFSVDYFALGVTLYEMIAARGPFR---AR   392
GRK1A_Opossum_XP-001364656                               NVN------EENPGFEEPRAVFYIAQIVCGMEHLHQKRIVYRDLKPENVLLDNDGNVRISDLGLAVELKEGQTKTKGYAGTPGFMAPELLRS-EEYDFSVDYFALGVTLYEMIAATGPFR---AR   396
GRK1A_Tasmanian_devil_XP-003765754                       NVN------EENPGFEEARAIFYIAQIICGMEHLHQKRIVYRDLKPENVLLDNDGNVRISDLGLAVELKEGETKTKGYAGTPGFMAPELLQS-EEYDFSVDYFALGVTLYEMIAARGPFR---AR   396
GRK1A_Xenopus_XP-002937018                               NMD------EENPGIDEHRACFYTAQIISGLEHLHQNRIIYRDLKPENVLLDNDGNVRISDLGLAVELKEKKSKAKGYAGTPGFMAPELLQG-EEYDYAVDYFSLGVTLYEMIAARGPFR---CR   395
GRK1A_Nanorana_XP-018428616                              NVD------EENPGLDEPRACFYTAQIICGMEHLHQNRIIYRDLKPENVLLDNDGNVRISDLGLAVELKEKKNKTKGYAGTPGFMAPELLNG-EEYDYSVDYFSLGVTLYEMIAARGPFR---CR   396
GRK1A_Florida_gar_24879-1-1                              NVD------ENNPGFEELRACFYTAQIICGMEHLHQKRIIYRDLKPENVLLDNDGNVRISDLGLAVELKDDQTKIKGYAGTPGFMAPELLKG-EEYDYSVDYFTLGVTLYEMVAAKGPFR---TR   396
GRK1A_Bowfin_29529-1-1                                   NVD------ENNPGFEEARACFYTAQIICGMEHLHQKRIIYRDLKPENVLLDNDGNVRISDLGLAVELKDDQTKIKGYAGTPGFMAPELLKG-EEYDYSVDYFTLGVTLYEMIEAKGPFR---TR   396
GRK1A_Zebrafish_NP-001029353                             NVD------ENNPGFSEPRACYYAAQIIQGLEHLHQKKIIYRDLKPENVLLDNEGNVRISDLGLAVELADGQEKTKGYAGTPGFMAPELLKG-EEYDYSVDYFTLGVTLYEFIAAKGPFR---TR   396
GRK1A-1_Medaka_NP-001188419                              NVD------ENNPGFDEPRACYYAAQIIQGLEHLHQKRIIYRDLKPENVLLDNEGHVRISDLGLAVELADDQFKIKGYAGTPGFMAPELLNG-EEYDYCVDYFTLGVTLYEFLAAKGPFR---TR   396
GRK1A-2_Medaka_XP-004081538                              LVD------ENNPGFDEPRACFYIAQIIQGLEHLHQKNIIYRDLKPENVLLDNDGNVRISDLGLAVELKEGKTKTKGYAGTPGYMAPEMLKG-DKYDSSVDYFTLGVTLYEFMAAKNPFR---NR   395
GRK1A_Reef_shark_53758-1-1                               NVD------ENNPGFDEKRACFYTAQIVCGMEHLHQSRIIYRDLKPENVLLDNEGHVRISDLGLAVELVDDQTKIKGYAGTPGFMAPELLKG-LEYDYSVDYFTLGVTLYEMIAAKGPFR---YR   397
GRK1A_Bamboo_shark_8046-1-1                              NVD------ENNPGFSEERACFYTAQIVCGMEHLHQSRIIYRDLKPENVLLDNEGHVRISDLGLAVELNDDQTKIKGYAGTPGFMAPELLKG-LEYDYSVDYFTLGVTLYEMIAAKGPFR---YR   397
GRK1A_Bluespot_ray_29212-2-1                             NVD------ENNPGFEEERARFYTAQIICGLEHLHQSRIIYRDLKPENVLLDNDGHVRISDLGLAVELKPDENKTKGYAGTPGFMAPELLGG-VEYGYSVDYFTLGVTLFEMIAAKGPFR---YR   396
GRK1A_Western_ray_41513-9-2                              NVD------ENNPGFDEERARFYTAQIVCGLEHLHQCRIIYRDLKPENVLLDNDGHVRISDLGLAVELKPNETKTKGYAGTPGFMAPELLEG-LEYDYSVDYFTLGVTLYEMIAAKGPFR---YR   396
GRK1A_Elephant_shark_XP-007889799                        NVD------ENNPGFGEDRACFYAAQIVCGMEHLHQNKIVYRDLKPENVLLDNEGHVRISDLGLAVELGENQTKTKGYAGTPGFMAPELLKS-EEYDYSVDYFTLGVTVYEMIAAKGPFR---TR   396
GRK_C.intestinalis_XP-009859302                          NMG------A--PGFSIHRAIFYSAEITCGLHDLHKQRIVYRDLKPENILLDDHGHIRISDLGLAVRIPSG-EKIKGRVGTVGYMAPEVVRN-ERYEMSPDWFGLGCIIYEMIAGQAPFR---KR   400
GRK_B.floridae_XP-019631688                              SMS------D-QPGFAESRAEFYAAEVLCGLQHLHQERIVYRDLKPENILLDDHGHVRISDLGLAVEIPEG-EMIRGRVGTVGYMAPEVVKN-EKYMFSPDWWGLGCLIYEMIEGKAPFR---AR   402
GRK_B.floridae_XP-002608270                              SMS------D-PPGFAESRAEFYAAEVLCGLQHLHQERIVYRDLKPENILLDDHGHVRISDLGLAVEIPEG-EMIRGRVGTVGYMAPEVVKN-EKYMFSPDWWGLGCLIYEMIEGKAPFR---AR   335
GRK6_Human_NP-002073                                     HMG------Q--AGFPEARAVFYAAEICCGLEDLHRERIVYRDLKPENILLDDHGHIRISDLGLAVHVPEG-QTIKGRVGTVGYMAPEVVKN-ERYTFSPDWWALGCLLYEMIAGQSPFQ---QR   388
GRK5_Human_NP-005299                                     NMG------N--PGFEEERALFYAAEILCGLEDLHRENTVYRDLKPENILLDDYGHIRISDLGLAVKIPEG-DLIRGRVGTVGYMAPEVLNN-QRYGLSPDYWGLGCLIYEMIEGQSPFR---GR   388
GRK4_Human_NP-892027                                     NLG------N--PGFDEQRAVFYAAELCCGLEDLQRERIVYRDLKPENILLDDRGHIRISDLGLATEIPEG-QRVRGRVGTVGYMAPEVVNN-EKYTFSPDWWGLGCLIYEMIQGHSPFK---KY   389
                                                         ..380.......390.......400.......410.......420.......430.......440.......450.......460.......470.......480.......490.......500
```

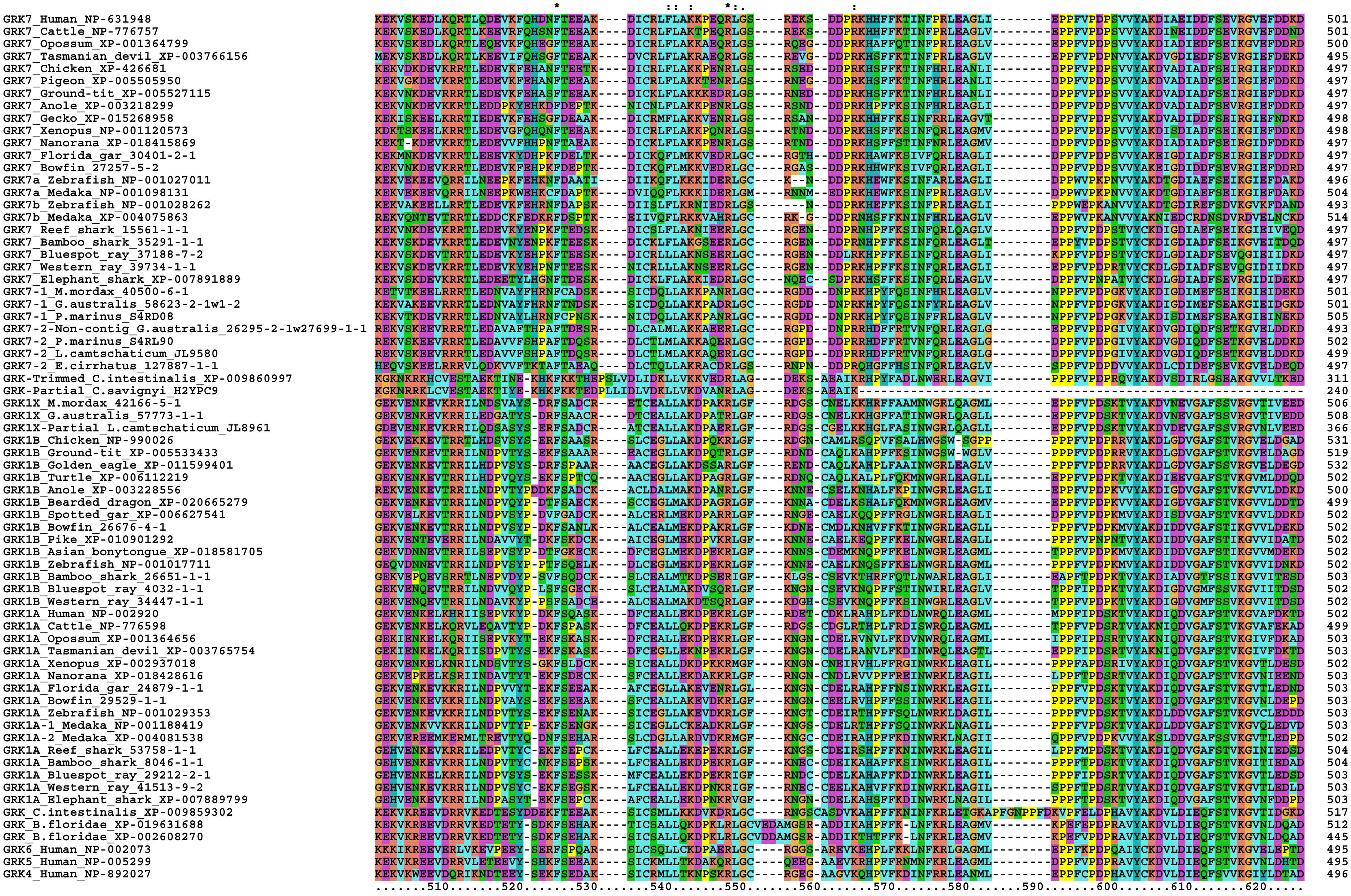
GRK7_Human_NP-631948 501
GRK7_Cattle_NP-776757 501
GRK7_Opossum_XP-001364799 500
GRK7_Tasmanian_devil_XP-003766156 495
GRK7_Chicken_XP-426681 497
GRK7_Pigeon_XP-005505950 497
GRK7_Ground-tit_XP-005527115 497
GRK7_Anole_XP-003218299 497
GRK7_Gecko_XP-015268958 498
GRK7_Xenopus_NP-001120573 498
GRK7_Nanorana_XP-018415869 497
GRK7_Florida_gar_30401-2-1 497
GRK7_Bowfin_27257-5-2 497
GRK7a_Zebrafish_NP-001027011 496
GRK7a_Medaka_NP-001098131 504
GRK7b_Zebrafish_NP-001028262 493
GRK7b_Medaka_XP-004075863 514
GRK7_Reef_shark_15561-1-1 497
GRK7_Bamboo_shark_35291-1-1 497
GRK7_Bluespot_ray_37188-7-2 497
GRK7_Western_ray_39734-1-1 497
GRK7_Elephant_shark_XP-007891889 497
GRK7-1_M.mordax_40500-6-1 501
GRK7-1_G.australis_58623-2-1w1-2 501
GRK7-1_P.marinus_S4RD08 505
GRK7-2-Non-contig_G.australis_26295-2-1w27699-1-1 493
GRK7-2_P.marinus_S4RL90 502
GRK7-2_L.camtschaticum_JL9580 499
GRK7-2_E.cirrhatus_127887-1-1 497
GRK-Trimmed_C.intestinalis_XP-009860997 311
GRK-Partial_C.savignyi_H2YPC9 240
GRK1X_M.mordax_42166-5-1 506
GRK1X_G.australis_57773-1-1 508
GRK1X-Partial_L.camtschaticum_JL8961 366
GRK1B_Chicken_NP-990026 531
GRK1B_Ground-tit_XP-005533433 519
GRK1B_Golden_eagle_XP-011599401 532
GRK1B_Turtle_XP-006112219 502
GRK1B_Anole_XP-003228556 500
GRK1B_Bearded_dragon_XP-020665279 499
GRK1B_Spotted_gar_XP-006627541 502
GRK1B_Bowfin_26676-4-1 502
GRK1B_Pike_XP-010901292 502
GRK1B_Asian_bonytongue_XP-018581705 502
GRK1B_Zebrafish_NP-001017711 502
GRK1B_Bamboo_shark_26651-1-1 503
GRK1B_Bluespot_ray_4032-1-1 502
GRK1B_Western_ray_34447-1-1 502
GRK1A_Human_NP-002920 502
GRK1A_Cattle_NP-776598 499
GRK1A_Opossum_XP-001364656 503
GRK1A_Tasmanian_devil_XP-003765754 503
GRK1A_Xenopus_XP-002937018 502
GRK1A_Nanorana_XP-018428616 503
GRK1A_Florida_gar_24879-1-1 503
GRK1A_Bowfin_29529-1-1 503
GRK1A_Zebrafish_NP-001029353 503
GRK1A-1_Medaka_NP-001188419 503
GRK1A-2_Medaka_XP-004081538 502
GRK1A_Reef_shark_53758-1-1 504
GRK1A_Bamboo_shark_8046-1-1 504
GRK1A_Bluespot_ray_29212-2-1 503
GRK1A_Western_ray_41513-9-2 503
GRK1A_Elephant_shark_XP-007889799 503
GRK_C.intestinalis_XP-009859302 517
GRK_B.floridae_XP-019631688 512
GRK_B.floridae_XP-002608270 445
GRK6_Human_NP-002073 495
GRK5_Human_NP-005299 495
GRK4_Human_NP-892027 496
.......510.......520.......530.......540.......550.......560.......570.......580.......590.......600.......610.......620.....

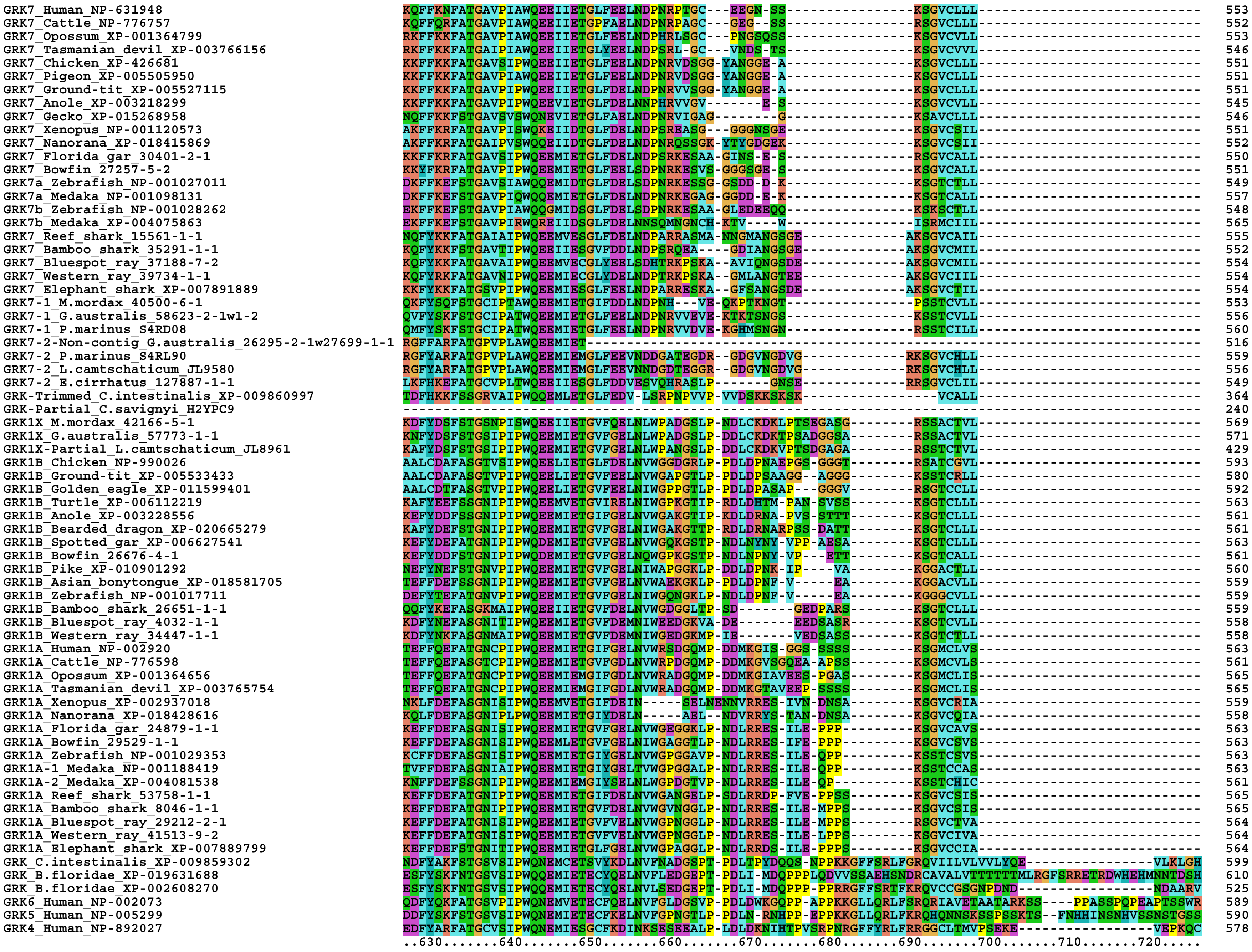

GRK7_Human_NP-631948 553
GRK7_Cattle_NP-776757 552
GRK7_Opossum_XP-001364799 553
GRK7_Tasmanian_devil_XP-003766156 546
GRK7_Chicken_XP-426681 551
GRK7_Pigeon_XP-005505950 551
GRK7_Ground-tit_XP-005527115 551
GRK7_Anole_XP-003218299 545
GRK7_Gecko_XP-015268958 546
GRK7_Xenopus_NP-001120573 551
GRK7_Nanorana_XP-018415869 552
GRK7_Florida_gar_30401-2-1 550
GRK7_Bowfin_27257-5-2 551
GRK7a_Zebrafish_NP-001027011 549
GRK7a_Medaka_NP-001098131 557
GRK7b_Zebrafish_NP-001028262 548
GRK7b_Medaka_XP-004075863 565
GRK7_Reef_shark_15561-1-1 555
GRK7_Bamboo_shark_35291-1-1 552
GRK7_Bluespot_ray_37188-7-2 554
GRK7_Western_ray_39734-1-1 554
GRK7_Elephant_shark_XP-007891889 554
GRK7-1_M.mordax_40500-6-1 553
GRK7-1_G.australis_58623-2-1w1-2 556
GRK7-1_P.marinus_S4RD08 560
GRK7-2-Non-contig_G.australis_26295-2-1w27699-1-1 516
GRK7-2_P.marinus_S4RL90 559
GRK7-2_L.camtschaticum_JL9580 556
GRK7-2_E.cirrhatus_127887-1-1 549
GRK-Trimmed_C.intestinalis_XP-009860997 364
GRK-Partial_C.savignyi_H2YPC9 240
GRK1X_M.mordax_42166-5-1 569
GRK1X_G.australis_57773-1-1 571
GRK1X-Partial_L.camtschaticum_JL8961 429
GRK1B_Chicken_NP-990026 593
GRK1B_Ground-tit_XP-005533433 580
GRK1B_Golden_eagle_XP-011599401 592
GRK1B_Turtle_XP-006112219 563
GRK1B_Anole_XP-003228556 561
GRK1B_Bearded_dragon_XP-020665279 561
GRK1B_Spotted_gar_XP-006627541 563
GRK1B_Bowfin_26676-4-1 561
GRK1B_Pike_XP-010901292 560
GRK1B_Asian_bonytongue_XP-018581705 559
GRK1B_Zebrafish_NP-001017711 559
GRK1B_Bamboo_shark_26651-1-1 559
GRK1B_Bluespot_ray_4032-1-1 558
GRK1B_Western_ray_34447-1-1 558
GRK1A_Human_NP-002920 563
GRK1A_Cattle_NP-776598 561
GRK1A_Opossum_XP-001364656 565
GRK1A_Tasmanian_devil_XP-003765754 565
GRK1A_Xenopus_XP-002937018 559
GRK1A_Nanorana_XP-018428616 558
GRK1A_Florida_gar_24879-1-1 563
GRK1A_Bowfin_29529-1-1 563
GRK1A_Zebrafish_NP-001029353 563
GRK1A-1_Medaka_NP-001188419 563
GRK1A-2_Medaka_XP-004081538 561
GRK1A_Reef_shark_53758-1-1 565
GRK1A_Bamboo_shark_8046-1-1 565
GRK1A_Bluespot_ray_29212-2-1 564
GRK1A_Western_ray_41513-9-2 564
GRK1A_Elephant_shark_XP-007889799 564
GRK_C.intestinalis_XP-009859302 599
GRK_B.floridae_XP-019631688 610
GRK_B.floridae_XP-002608270 525
GRK6_Human_NP-002073 589
GRK5_Human_NP-005299 590
GRK4_Human_NP-892027 578
..630.......640.......650.......660.......670.......680.......690.......700.......710.......720.....

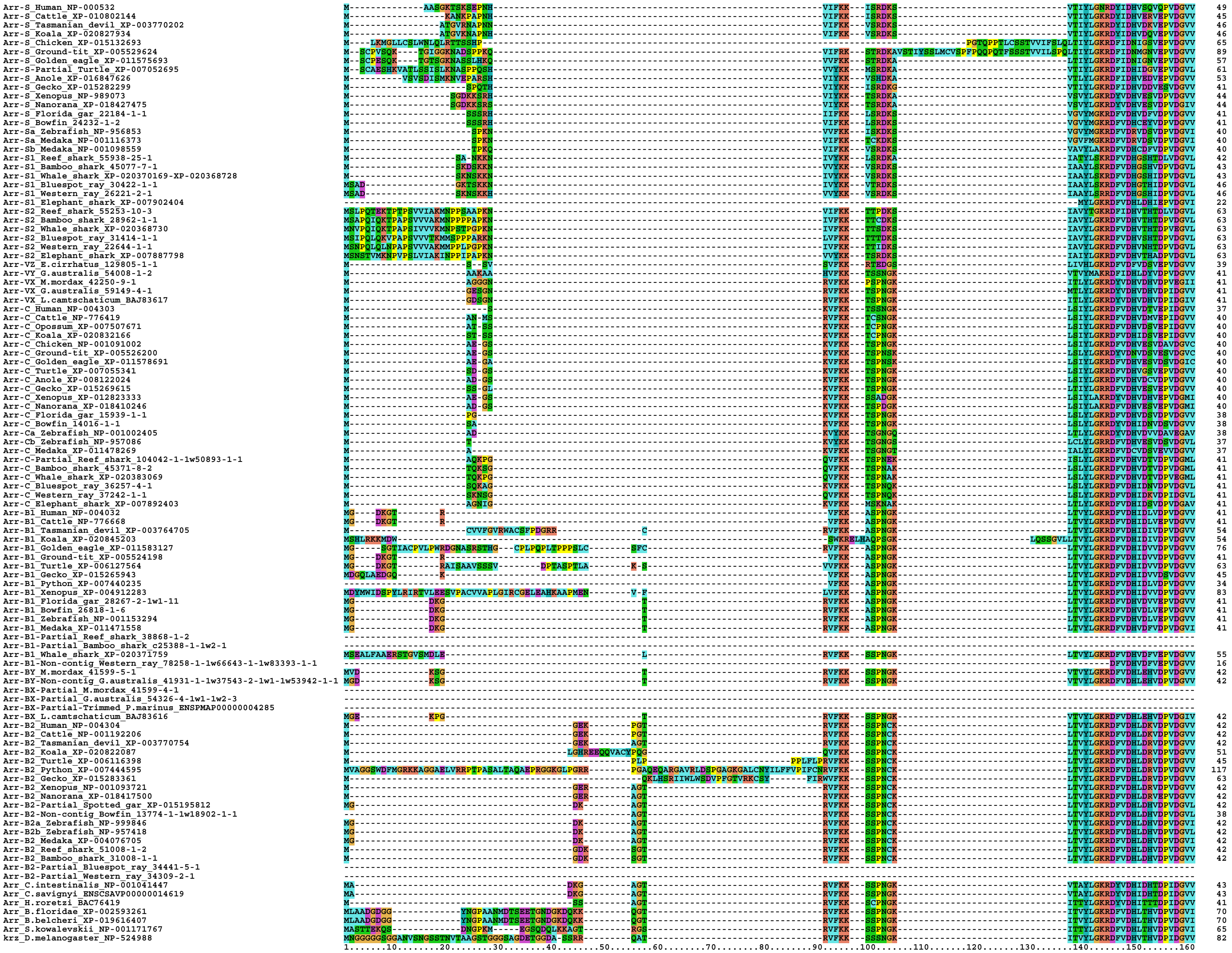
Arr-S_Human_NP-000532
Arr-S_Cattle_XP-010802144
Arr-S_Tasmanian_devil_XP-003770202
Arr-S_Koala_XP-020827934
Arr-S_Chicken_XP-015132693
Arr-S_Ground-tit_XP-005529624
Arr-S_Golden_eagle_XP-011575693
Arr-S-Partial_Turtle_XP-007052695
Arr-S_Anole_XP-016847626
Arr-S_Gecko_XP-015282299
Arr-S_Xenopus_NP-989073
Arr-S_Nanorana_XP-018427475
Arr-S_Florida_gar_22184-1-1
Arr-S_Bowfin_24232-1-2
Arr-Sa_Zebrafish_NP-956853
Arr-Sa_Medaka_NP-001116373
Arr-Sb_Medaka_NP-001098559
Arr-S1_Reef_shark_55938-25-1
Arr-S1_Bamboo_shark_45077-7-1
Arr-S1_Whale_shark_XP-020370169-XP-020368728
Arr-S1_Bluespot_ray_30422-1-1
Arr-S1_Western_ray_26221-2-1
Arr-S1_Elephant_shark_XP-007902404
Arr-S2_Reef_shark_55253-10-3
Arr-S2_Bamboo_shark_28962-1-1
Arr-S2_Whale_shark_XP-020368730
Arr-S2_Bluespot_ray_31414-1-1
Arr-S2_Western_ray_22644-1-1
Arr-S2_Elephant_shark_XP-007887798
Arr-VZ_E.cirrhatus_129805-1-1
Arr-VY_G.australis_54008-1-2
Arr-VX_M.mordax_42250-9-1
Arr-VX_G.australis_59149-4-1
Arr-VX_L.camtschaticum_BAJ83617
Arr-C_Human_NP-004303
Arr-C_Cattle_NP-776419
Arr-C_Opossum_XP-007507671
Arr-C_Koala_XP-020832166
Arr-C_Chicken_NP-001091002
Arr-C_Ground-tit_XP-005526200
Arr-C_Golden_eagle_XP-011578691
Arr-C_Turtle_XP-007055341
Arr-C_Anole_XP-008122024
Arr-C_Gecko_XP-015269615
Arr-C_Xenopus_XP-012823333
Arr-C_Nanorana_XP-018410246
Arr-C_Florida_gar_15939-1-1
Arr-C_Bowfin_14016-1-1
Arr-Ca_Zebrafish_NP-001002405
Arr-Cb_Zebrafish_NP-957086
Arr-C_Medaka_XP-011478269
Arr-C-Partial_Reef_shark_104042-1-1w50893-1-1
Arr-C_Bamboo_shark_45371-8-2
Arr-C_Whale_shark_XP-020383069
Arr-C_Bluespot_ray_36257-4-1
Arr-C_Western_ray_37242-1-1
Arr-C_Elephant_shark_XP-007892403
Arr-B1_Human_NP-004032
Arr-B1_Cattle_NP-776668
Arr-B1_Tasmanian_devil_XP-003764705
Arr-B1_Koala_XP-020845203
Arr-B1_Golden_eagle_XP-011583127
Arr-B1_Ground-tit_XP-005524198
Arr-B1_Turtle_XP-006127564
Arr-B1_Gecko_XP-015265943
Arr-B1_Python_XP-007440235
Arr-B1_Xenopus_XP-004912283
Arr-B1_Florida_gar_28267-2-1w1-11
Arr-B1_Bowfin_26818-1-6
Arr-B1_Zebrafish_NP-001153294
Arr-B1_Medaka_XP-011471558
Arr-B1-Partial_Reef_shark_38868-1-2
Arr-B1-Partial_Bamboo_shark_c25388-1-1w2-1
Arr-B1_Whale_shark_XP-020371759
Arr-B1-Non-contig_Western_ray_78258-1-1w66643-1-1w83393-1-1
Arr-BY_M.mordax_41599-5-1
Arr-BY-Non-contig_G.australis_41931-1-1w37543-2-1w1-1w53942-1-1
Arr-BX-Partial_M.mordax_41599-4-1
Arr-BX-Partial_G.australis_54326-4-1w1-1w2-3
Arr-BX-Partial-Trimmed_P.marinus_ENSPMAP00000004285
Arr-BX_L.camtschaticum_BAJ83616
Arr-B2_Human_NP-004304
Arr-B2_Cattle_NP-001192206
Arr-B2_Tasmanian_devil_XP-003770754
Arr-B2_Koala_XP-020822087
Arr-B2_Turtle_XP-006116398
Arr-B2_Python_XP-007444595
Arr-B2_Gecko_XP-015283361
Arr-B2_Xenopus_NP-001093721
Arr-B2_Nanorana_XP-018417500
Arr-B2-Partial_Spotted_gar_XP-015195812
Arr-B2-Non-contig_Bowfin_13774-1-1w18902-1-1
Arr-B2a_Zebrafish_NP-999846
Arr-B2b_Zebrafish_NP-957418
Arr-B2_Medaka_XP-004076705
Arr-B2_Reef_shark_51008-1-2
Arr-B2_Bamboo_shark_31008-1-1
Arr-B2-Partial_Bluespot_ray_34441-5-1
Arr-B2-Partial_Western_ray_34309-2-1
Arr_C.intestinalis_NP-001041447
Arr_C.savignyi_ENSCSAVP00000014619
Arr_H.roretzi_BAC76419
Arr_B.floridae_XP-002593261
Arr_B.belcheri_XP-019616407
Arr_S.kowalevskii_NP-001171767
krz_D.melanogaster_NP-524988

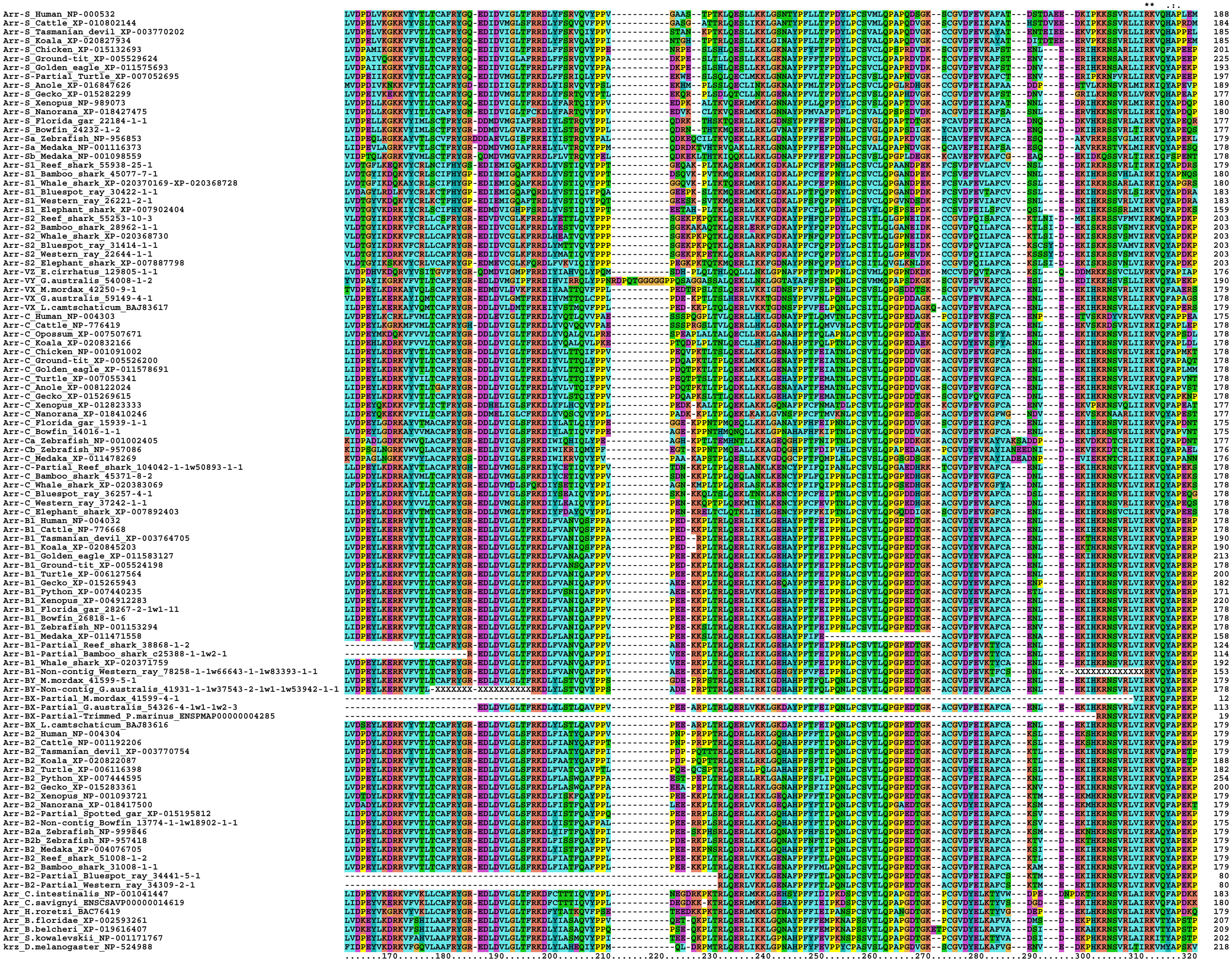

Arr-S_Human_NP-000532 188
Arr-S_Cattle_XP-010802144 184
Arr-S_Tasmanian_devil_XP-003770202 185
Arr-S_Koala_XP-020827934 185
Arr-S_Chicken_XP-015132693 201
Arr-S_Ground-tit_XP-005529624 225
Arr-S_Golden_eagle_XP-011575693 193
Arr-S-Partial_Turtle_XP-007052695 197
Arr-S_Anole_XP-016847626 189
Arr-S_Gecko_XP-015282299 177
Arr-S_Xenopus_NP-989073 180
Arr-S_Nanorana_XP-018427475 180
Arr-S_Florida_gar_22184-1-1 177
Arr-S_Bowfin_24232-1-2 177
Arr-Sa_Zebrafish_NP-956853 179
Arr-Sa_Medaka_NP-001116373 178
Arr-Sb_Medaka_NP-001098559 178
Arr-S1_Reef_shark_55938-25-1 179
Arr-S1_Bamboo_shark_45077-7-1 180
Arr-S1_Whale_shark_XP-020370169-XP-020368728 180
Arr-S1_Bluespot_ray_30422-1-1 183
Arr-S1_Western_ray_26221-2-1 183
Arr-S1_Elephant_shark_XP-007902404 159
Arr-S2_Reef_shark_55253-10-3 203
Arr-S2_Bamboo_shark_28962-1-1 203
Arr-S2_Whale_shark_XP-020368730 203
Arr-S2_Bluespot_ray_31414-1-1 203
Arr-S2_Western_ray_22644-1-1 203
Arr-S2_Elephant_shark_XP-007887798 203
Arr-VZ_E.cirrhatus_129805-1-1 176
Arr-VY_G.australis_54008-1-2 190
Arr-VX_M.mordax_42250-9-1 179
Arr-VX_G.australis_59149-4-1 178
Arr-VX_L.camtschaticum_BAJ83617 179
Arr-C_Human_NP-004303 175
Arr-C_Cattle_NP-776419 178
Arr-C_Opossum_XP-007507671 178
Arr-C_Koala_XP-020832166 178
Arr-C_Chicken_NP-001091002 178
Arr-C_Ground-tit_XP-005526200 178
Arr-C_Golden_eagle_XP-011578691 178
Arr-C_Turtle_XP-007055341 178
Arr-C_Anole_XP-008122024 178
Arr-C_Gecko_XP-015269615 178
Arr-C_Xenopus_XP-012823333 177
Arr-C_Nanorana_XP-018410246 177
Arr-C_Florida_gar_15939-1-1 175
Arr-C_Bowfin_14016-1-1 175
Arr-Ca_Zebrafish_NP-001002405 177
Arr-Cb_Zebrafish_NP-957086 176
Arr-C_Medaka_XP-011478269 176
Arr-C-Partial_Reef_shark_104042-1-1w50893-1-1 178
Arr-C_Bamboo_shark_45371-8-2 178
Arr-C_Whale_shark_XP-020383069 178
Arr-C_Bluespot_ray_36257-4-1 178
Arr-C_Western_ray_37242-1-1 178
Arr-C_Elephant_shark_XP-007892403 178
Arr-B1_Human_NP-004032 178
Arr-B1_Cattle_NP-776668 178
Arr-B1_Tasmanian_devil_XP-003764705 190
Arr-B1_Koala_XP-020845203 190
Arr-B1_Golden_eagle_XP-011583127 213
Arr-B1_Ground-tit_XP-005524198 178
Arr-B1_Turtle_XP-006127564 200
Arr-B1_Gecko_XP-015265943 182
Arr-B1_Python_XP-007440235 171
Arr-B1_Xenopus_XP-004912283 220
Arr-B1_Florida_gar_28267-2-1w1-11 178
Arr-B1_Bowfin_26818-1-6 178
Arr-B1_Zebrafish_NP-001153294 178
Arr-B1_Medaka_XP-011471558 158
Arr-B1-Partial_Reef_shark_38868-1-2 124
Arr-B1-Partial_Bamboo_shark_c25388-1-1w2-1 114
Arr-B1_Whale_shark_XP-020371759 192
Arr-B1-Non-contig_Western_ray_78258-1-1w66643-1-1w83393-1-1 153
Arr-BY_M.mordax_41599-5-1 179
Arr-BY-Non-contig_G.australis_41931-1-1w37543-2-1w1-1w53942-1-1 178
Arr-BX-Partial_M.mordax_41599-4-1 12
Arr-BX-Partial_G.australis_54326-4-1w1-1w2-3 113
Arr-BX-Partial-Trimmed_P.marinus_ENSPMAP00000004285 19
Arr-BX_L.camtschaticum_BAJ83616 179
Arr-B2_Human_NP-004304 179
Arr-B2_Cattle_NP-001192206 179
Arr-B2_Tasmanian_devil_XP-003770754 179
Arr-B2_Koala_XP-020822087 188
Arr-B2_Turtle_XP-006116398 182
Arr-B2_Python_XP-007444595 254
Arr-B2_Gecko_XP-015283361 200
Arr-B2_Xenopus_NP-001093721 179
Arr-B2_Nanorana_XP-018417500 179
Arr-B2-Partial_Spotted_gar_XP-015195812 179
Arr-B2-Non-contig_Bowfin_13774-1-1w18902-1-1 175
Arr-B2a_Zebrafish_NP-999846 179
Arr-B2b_Zebrafish_NP-957418 179
Arr-B2_Medaka_XP-004076705 179
Arr-B2_Reef_shark_51008-1-2 179
Arr-B2_Bamboo_shark_31008-1-1 179
Arr-B2-Partial_Bluespot_ray_34441-5-1 80
Arr-B2-Partial_Western_ray_34309-2-1 80
Arr_C.intestinalis_NP-001041447 183
Arr_C.savignyi_ENSCSAVP00000014619 180
Arr_H.roretzi_BAC76419 179
Arr_B.floridae_XP-002593261 207
Arr_B.belcheri_XP-019616407 209
Arr_S.kowalevskii_NP-001171767 202
krz_D.melanogaster_NP-524988 218
.......170.......180.......190.......200.......210.......220.......230.......240.......250.......260.......270.......280.......290.......300.......310.......320

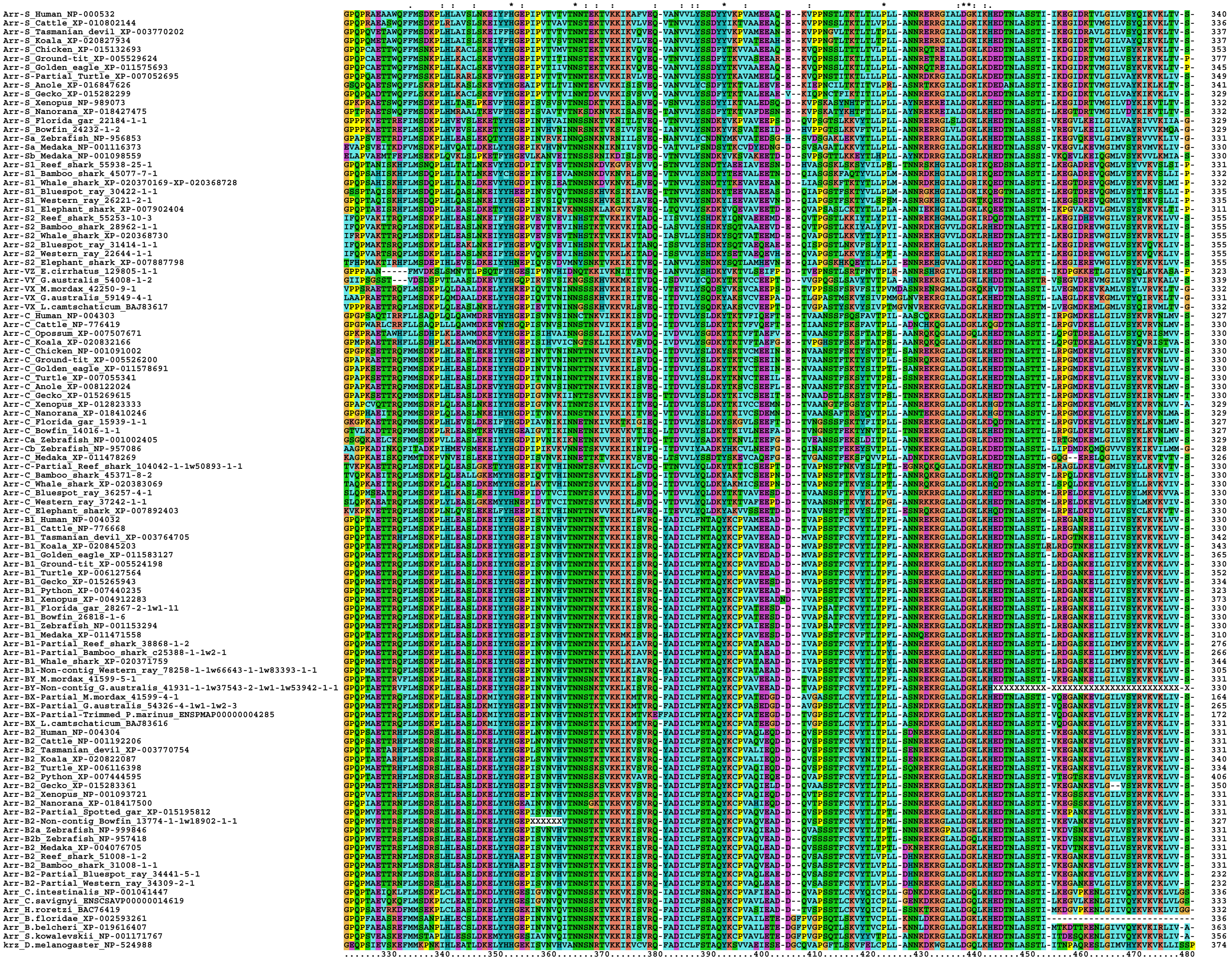
Arr-S_Human_NP-000532 340
Arr-S_Cattle_XP-010802144 336
Arr-S_Tasmanian_devil_XP-003770202 337
Arr-S_Koala_XP-020827934 337
Arr-S_Chicken_XP-015132693 353
Arr-S_Ground-tit_XP-005529624 377
Arr-S_Golden_eagle_XP-011575693 345
Arr-S-Partial_Turtle_XP-007052695 349
Arr-S_Anole_XP-016847626 341
Arr-S_Gecko_XP-015282299 329
Arr-S_Xenopus_NP-989073 332
Arr-S_Nanorana_XP-018427475 332
Arr-S_Florida_gar_22184-1-1 329
Arr-S_Bowfin_24232-1-2 329
Arr-Sa_Zebrafish_NP-956853 331
Arr-Sa_Medaka_NP-001116373 330
Arr-Sb_Medaka_NP-001098559 330
Arr-S1_Reef_shark_55938-25-1 331
Arr-S1_Bamboo_shark_45077-7-1 332
Arr-S1_Whale_shark_XP-020370169-XP-020368728 332
Arr-S1_Bluespot_ray_30422-1-1 335
Arr-S1_Western_ray_26221-2-1 335
Arr-S1_Elephant_shark_XP-007902404 311
Arr-S2_Reef_shark_55253-10-3 355
Arr-S2_Bamboo_shark_28962-1-1 355
Arr-S2_Whale_shark_XP-020368730 355
Arr-S2_Bluespot_ray_31414-1-1 355
Arr-S2_Western_ray_22644-1-1 355
Arr-S2_Elephant_shark_XP-007887798 355
Arr-VZ_E.cirrhatus_129805-1-1 323
Arr-VY_G.australis_54008-1-2 339
Arr-VX_M.mordax_42250-9-1 332
Arr-VX_G.australis_59149-4-1 331
Arr-VX_L.camtschaticum_BAJ83617 332
Arr-C_Human_NP-004303 327
Arr-C_Cattle_NP-776419 330
Arr-C_Opossum_XP-007507671 330
Arr-C_Koala_XP-020832166 330
Arr-C_Chicken_NP-001091002 330
Arr-C_Ground-tit_XP-005526200 330
Arr-C_Golden_eagle_XP-011578691 330
Arr-C_Turtle_XP-007055341 330
Arr-C_Anole_XP-008122024 330
Arr-C_Gecko_XP-015269615 330
Arr-C_Xenopus_XP-012823333 329
Arr-C_Nanorana_XP-018410246 329
Arr-C_Florida_gar_15939-1-1 327
Arr-C_Bowfin_14016-1-1 327
Arr-Ca_Zebrafish_NP-001002405 329
Arr-Cb_Zebrafish_NP-957086 328
Arr-C_Medaka_XP-011478269 326
Arr-C-Partial_Reef_shark_104042-1-1w50893-1-1 330
Arr-C_Bamboo_shark_45371-8-2 330
Arr-C_Whale_shark_XP-020383069 330
Arr-C_Bluespot_ray_36257-4-1 330
Arr-C_Western_ray_37242-1-1 330
Arr-C_Elephant_shark_XP-007892403 330
Arr-B1_Human_NP-004032 330
Arr-B1_Cattle_NP-776668 330
Arr-B1_Tasmanian_devil_XP-003764705 342
Arr-B1_Koala_XP-020845203 343
Arr-B1_Golden_eagle_XP-011583127 365
Arr-B1_Ground-tit_XP-005524198 330
Arr-B1_Turtle_XP-006127564 352
Arr-B1_Gecko_XP-015265943 334
Arr-B1_Python_XP-007440235 323
Arr-B1_Xenopus_XP-004912283 373
Arr-B1_Florida_gar_28267-2-1w1-11 330
Arr-B1_Bowfin_26818-1-6 330
Arr-B1_Zebrafish_NP-001153294 330
Arr-B1_Medaka_XP-011471558 310
Arr-B1-Partial_Reef_shark_38868-1-2 276
Arr-B1-Partial_Bamboo_shark_c25388-1-1w2-1 266
Arr-B1_Whale_shark_XP-020371759 344
Arr-B1-Non-contig_Western_ray_78258-1-1w66643-1-1w83393-1-1 305
Arr-BY_M.mordax_41599-5-1 331
Arr-BY-Non-contig_G.australis_41931-1-1w37543-2-1w1-1w53942-1-1 330
Arr-BX-Partial_M.mordax_41599-4-1 164
Arr-BX-Partial_G.australis_54326-4-1w1-1w2-3 265
Arr-BX-Partial-Trimmed_P.marinus_ENSPMAP00000004285 172
Arr-BX_L.camtschaticum_BAJ83616 331
Arr-B2_Human_NP-004304 331
Arr-B2_Cattle_NP-001192206 331
Arr-B2_Tasmanian_devil_XP-003770754 331
Arr-B2_Koala_XP-020822087 340
Arr-B2_Turtle_XP-006116398 334
Arr-B2_Python_XP-007444595 406
Arr-B2_Gecko_XP-015283361 350
Arr-B2_Xenopus_NP-001093721 331
Arr-B2_Nanorana_XP-018417500 331
Arr-B2-Partial_Spotted_gar_XP-015195812 331
Arr-B2-Non-contig_Bowfin_13774-1-1w18902-1-1 327
Arr-B2a_Zebrafish_NP-999846 331
Arr-B2b_Zebrafish_NP-957418 331
Arr-B2_Medaka_XP-004076705 331
Arr-B2_Reef_shark_51008-1-2 331
Arr-B2_Bamboo_shark_31008-1-1 331
Arr-B2-Partial_Bluespot_ray_34441-5-1 232
Arr-B2-Partial_Western_ray_34309-2-1 232
Arr_C.intestinalis_NP-001041447 336
Arr_C.savignyi_ENSCSAVP00000014619 333
Arr_H.roretzi_BAC76419 332
Arr_B.floridae_XP-002593261 336
Arr_B.belcheri_XP-019616407 363
Arr_S.kowalevskii_NP-001171767 356
krz_D.melanogaster_NP-524988 374
.......330.......340.......350.......360.......370.......380.......390.......400.......410.......420.......430.......440.......450.......460.......470.......480

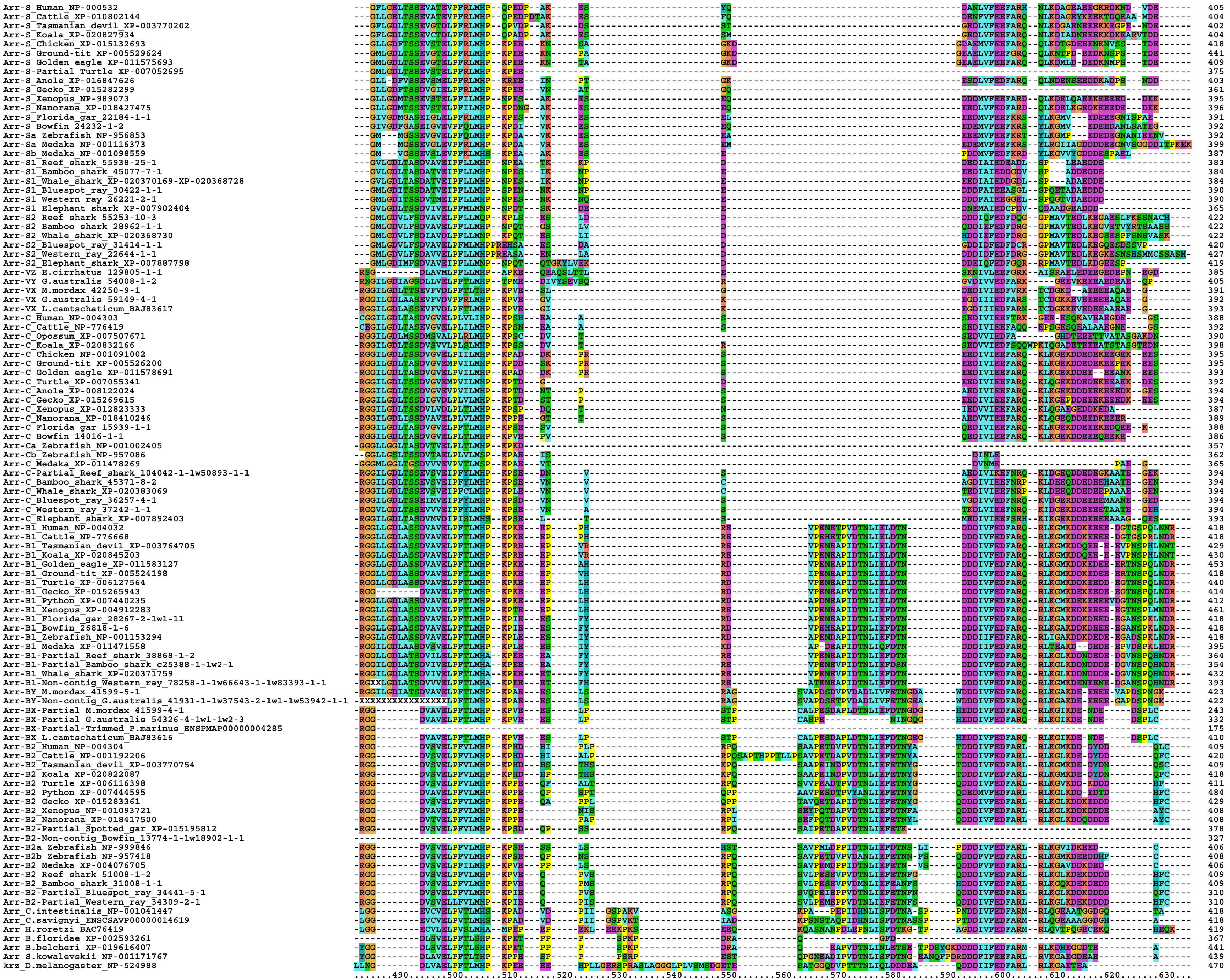

```
RGS9_Human_NP-001159405                     MT--------IRH----Q----GQQYRPRMAFLQKIEALVKDMQNPET-GVRMQNQRVLVTSVPHAMTGSDVLQWIVQRLWISSL-EAQNLGNFIVRYGYIYPLQDPKNLILKPD-GSLY  101
RGS9_Cattle_NP-776595                       MT--------IRH----Q----GQQYRPRMAFLRKIEALVKDMQDPDT-GVRVQNQKVKVVSIPHAMTGSDVLQWISQRLWISGL-EAQNLGNFIVKYGYIYPLQDPRNLTLKPD-SSLY  101
RGS9_Koala_XP-020856566                     MT--------IKH----Q----GQQYRPRMAFLRKIEDLVKEMQNPET-GIRMQSQRVMITNIPHAMTGNDILQWIAQRLWIGNI-EAQNLGNFIVKYGYIYPLQDPKNLILKPESNSLY 102
RGS9_Opossum_XP-007482817                   MT--------IRH----Q----GQQYRPRMAFLRKIEALVKEMQNPET-GIRMQSQRVMITNIPHAMTGNDILQWIAQRLWIGHM-EAQNLGNFVVKYGYVYPLQDPKNLNLKPDNSSLY 102
RGS9_Pigeon_XP-013225676                    -----------------------------MAFLKKIEALMMEMQNPDT-GVKTQTQRVMITNIPHAVAGNDVLQWILQRLQITQE-EALHLGDLFVKYGYIYPLQEPKNLTLKTD-GSLY  88
RGS9_Quail_XP-015735540                     MT--------VARL--DQ----GQRYRPRMAFLKKIEALIMEMQNPDT-GIKTQTQRVMITNIPHAVAGNDIFQWILQRLQITNE-EALHLGDLFVKYGYIYPLQEPKNLTLRTD-GILY  103
RGS9_Ground-tit_XP-014111246                MT--------VTRL--DQ----GQRYRPRMAFLKKIETLMMEMQNPDT-GIKTQTQTVMITNIPHAVTGVDIMQWILQRLQITAE-EALHLGDLFVKYGYIYPLQEPKNLTLKAD-GSLY  103
RGS9_Turtle_XP-014424786                    ----------------------------------------MEMQNPDT-GIKMHTQRVMITNIPHAMTGSDILQWIMQHLKIVEE-EALNLGNLLVKYGYIYPLQEPKNLILKTD-SSLY  77
RGS9_Python_XP-007421674                    MT--------ITHI--DQ----GQRYRPRMAFLKKLEILLVEMQDPHS-GIKIQTQKIINTSIPHAMTGSDILKWIMQRLQITSE-EALNLGSMIIKCGYIYPLQERKNLTLRAD-SSLY  103
RGS9_Garter_snake_XP-013926935              MT--------IAQI--DQ----GQRYRPRMAFLKKLEVLLVEMQDPQS-GIKTQIQNMPENSIPHTVVGCDMLQWIVKRLEITNE-EGLHLGSMMVKCGYVYPLQDRKNLNLKGD-TSLY  103
RGS9_X.laevis_XP-018090999                  MT--------VKHL--DQ----GQHFRPRMAFLKKIEALLLEMQDADT-GIKTHTQRLMITTIPHAVTGHDILEWLIQRLQIADE-EAQHLGNLMVRCGYIYPLQEPTNLVLNVD-SSLY  103
RGS9_Florida_gar_21856-1-2                  MT--------INKLC-DY----SQHYRPRMACLKKIESLVVEMQDPNN-GIKTQTQRLVITTIPHAITGLDLLQWLANRLQVETE-EAQNIGNLVVQYGYIYPLQDHKNLILKPD-GSLY  104
RGS9_Bowfin_26690-1-1                       MT--------INKLC-DH----SQKYRPRMACLKKIESLVVEMQDPNS-GIKTQTQRLVITTIPHAITGLDILQWIINRLQLENE-EAQNTCNLLVQYGYIYPLQEHKNLILKPD-ASLY  104
RGS9_Zebrafish_NP-001314729                 MT--------IRSDR-DH----GQHYRPRMTCLKKLEAMVVQMQDPST-GVKGSDQKLNVTTIPHVMTDQDIIAWIAKHMSMENN-EAQAFGTMLVAYGYIYPLQDHKKLVLKSD-GSLY  104
RGS9_Medaka_XP-004071947                    MT--------IRTVRPDR----GQHFHPRMHCLKKLEATVVEMQDPKT-GVKGSEQKLNVTTIPHVIAGNDIIAWITNKMKVTQE-EAGVFGTMLVAYGYVYPLQNHKKLVMCND-GSLY  105
RGS9_Reef_shark_51239-1-1                   MT--------IKSF--NQ----GQHYRPRMAFLRKIEPLMMEMQDPNT-GIKTQAQRLVITTIPHAMTGHDISEWLIQRLQITDE-EGYVLGNMIVKLGYIYPLQEPKNLVLRQD-SSLY  103
RGS9_Bamboo_shark_45486-8-1                 MT--------IRNF--NQ----GQQYRPRMAFLRKIEPLIKEMQDPNT-GIKTQTQRLIITTIPHAMTGQDVLEWLIQRLQITDE-EGYILGNMIVKLGYIYPLQEPRNLVLKHD-NSLY  103
RGS9_Bluespot_ray_37076-2-1                 MT--------VKSF--NQ----GQQFRPRMAFLRKIEPLIMEMQDPGT-GIKTQTQRLMITTIPHAMTGNDVLEWLIHRLQVTDE-EGSLLGNLLVKLGYIYPLQEPKNLVLKPD-NSLY  103
RGS9_Western_ray_41046-8-8                  MT--------VKSF--NQ----GQQFRPRMAFLRKIEPLIMEMQNPGT-GIKTQTQRLMITTIPHAMTGNDVLEWLIQRLQVTEE-EGSLLGNLLVKLGYIYPLQEPRNLVLRPD-NSLY  103
RGS9_Elephant_shark_XP-007886905            MT--------IKSL--SQ----GQHHRPRMAFLKKIESLVIEMQDPEN-GIKTQTQRVMIATIPHALTGQDLLEWLIQRLQIEDE-ECQNLGNLIVNLGYLYPLQEPKNLILRPD-SSLY  103
RGS9_M.mordax_34174-1-2                     MK--------IHRL--EQ----NQRHRPRQHHLAKLGALVVTMQDSEH-GVPMRDQLLGDAVIPDSTTGRDLVQWLEQRLQVTAD-EAQQLGWLLVLCGFIYPLCEPRNLVLRAD-DSFY  103
RGS9_G.australis_56905-1-2                  MK--------INKL--DQ----SQQHRPRQQHLTKLGALVVSMQDPER-GVPMRDQLLSDTIIPDATTGHDLMRWIEQRLQITAD-EALQLCWLLVACGFVYPLSEPRNLAMRPD-GSLY  103
RGS9_E.cirrhatus_137654-1-3                 MK--------INKL--DQ----GQHYRPRMAFIKKLQSLVGAMQKRED-GVIFRNQRLITTTIPHAIMGFDLLEWFIESLQITYE-EAIHLGTLVICNGFIYPVSEPRDLTLRND-SSLY  103
RGS11_Human_NP-899180                       MA--------AGP----AP--PPGRPRAQMPHLRKMERVVVSMQDPDQ-GVKMRSQRLLVTVIPHAVTGSDVVQWLAQKFCVSEE-EALHLGAVLVQHGYIYPLRDPRSLMLRPD-ETPY  103
RGS11_Cattle_NP-001069282                   MA--------ASP----AP--LGGHPRVQRPHLSKMERMVVTMQDPDQ-GVRMRSQRLLVTVIPHAVNGSDIVEWLMQKYSIAED-EALHLGSLLVQHGYLYPLRDPRRLELRPD-ETPY  103
RGS11_Koala_XP-020862219                    MC--------VHKGFRWSLAWSFSRSNGSLLKSFQMERVIVGMQDPDM-GVKMRNQRLLITVIPHAMTGSDIVEWLIQKFSISEE-ESLHLGNLIVKHGYIYPLKDPRCLVLRAD-DSPY  109
RGS11_Opossum_XP-001372371                  MT--------INS----LH--QGGSYKVQMPCLSKMERVIVGMQDPDM-GVKMRNQRLLITVIPHAMTGHDIVEWLIQKYSISEE-ESLHLGNLIVKHGYIYPLKDPRCLVLRAD-DSPY  103
RGS11_Chicken_XP-001231808                  MT--------IGG----PR--GGGQPRLQMPCLGKMERMIVSMQDPDM-GIKMRNQRLLITVIPHAMTGNDIVEWLIQKYGISEE-ESLHLGNLIVRHGYIYPLRDPRSLVLRAD-ESPY  103
RGS11_Pigeon_XP-005508486                   -----------------------------------MERMIVSMQDPDM-GIKMRNQRLLITVIPHAMTGNDIVEWLIQKYGISEE-ESLHLGNLIVKHGYIYPLRDPRSLVLRAD-ESPY  82
RGS11_Ground-tit_XP-014107999               -----------------------------------MERMIVSMQDPDM-GIKMRNQRLLITVIPHAMTGNDIVEWLIQKYGISEE-ESLHLGNLIVKHGYIYPLKDPRSLVLRAD-ESPY  82
RGS11_Turtle_XP-007066592                   -----------------------------------MEHVIVSMQDPDM-GIKMRNQRLLITVIPHAMTGNDIVEWLIQKYAISEE-ESLHLGNLLVKHGYIYPLKDPRSLVLRAD-DSPY  82
RGS11_Alligator_XP-014457643                -----------------------------------MERMIVIMQDPDM-GVKMRNQRLLITVIPHAMTGNDIVEWLIQKYTISEE-ESLHLGNLIVKHGYIYPLKDPRNLVLRAD-ESPY  82
RGS11_Anole_XP-003227697                    MT--------ISA----LRPGAGGNSRLQLPCLAKMERVVVGMQDPDM-GVKVRSQRLLITVIPHALTGSDIVQWLVQKYAISEE-EALHLGNLIVRHGYIYPLKDPRSLALRPD-ETPY  105
RGS11_Nanorana_XP-018423234                 -----------------------------------MERLIVAMQDPDI-GIKMRNQRLLITVIPHAMTGNDVVEWLIQKLSINEE-ESLHLGNLLVKYGYIYPLKDPRNLILRAD-DSPY  82
RGS11_Xenopus_NP-001096497                  MT--------INS----LQ--PGKNCKVQMSCLAKMERLVVSMQDPDM-GIKMRNQRLLITVIPHAMTGNDVVEWLIQKLSISEE-EAQHLGNLLVRYGYIYPLKDPRNLILRAD-DSPY  103
RGS11_Florida_gar_29521-1-2                 MT--------INT------LSQGGNYKVQMPCFTKMERVIVAMQDPDM-GIKMRNQRLLITVIPHAVTGNDIVEWLIQKFSITEE-EALHVGNLIVKFGYIYPLKDPKTLILRPD-DSPY  103
RGS11_Bowfin_26311-1-2w1-5                  MT--------INT------LKQGGSYKVQMPCLTRMEHIIVAMQDPDM-GIKMRNQRLLITVIPHAMTGNDIVEWLIQKFSITEE-EALHVGNLIVKYGYIYPLKDPKTLILRPD-ESPY  103
RGS11_Zebrafish_XP-005170921                MT--------INT------LSQDGVQRQQMPCLTKMERMIISMQDPDM-GIKLRNQRLLITVIPHAMTGADIVEWLIKKLTISDE-EAAHIGQFLVKYGYIYPLKEPRNLILKAD-ESAY  103
RGS11_Medaka_XP-011484761                   MT--------INT------MAKGGWQREQMICFRMMEKMIVAMQDPDM-GMKLRNQRLLITVIPHAMTGHDIIIWLIHKYSVCEE-EALHLGGMLVKYGYIYPLKEPRSLVLQPD-ESPY  103
RGS11_Reef_shark_50711-1-1                  ------------S-------ARIHTATIQMACLNKMEKVILAMQDPDI-GIKMKYQKLLITAIPHAVTGSDVVEWLLQNLSISEE-ESLHLGNLIVKYGYIYPLKDSKNLILRPD-DSPY  98
RGS11_Bamboo_shark_41815-1-3                MT--------IHT-------SSTHTTKIQMACLAKMEKVIVAMQDPDV-GIKMKYQKLIITGIPHAVTGNDIVEWLIQNFSISEE-ESLHLGDLIVKYGYIYPLKDPKNFVLRAD-DSPY  102
RGS11_Bluespot_ray_32308-1-2                MT--------IHG-------TGSQLGGAQMACLAKMETLIVAMQDPDT-GIKMRIQRFLV-PIPHLMTGNDIFQWLLQNVSATEE-ESFHLGDLIIKYGYIYPLKDPRSLVLRVD-DSPY  101
RGS11_Western_ray_41178-3-3                 MT--------IHN-------TRSHIGSAQMACLAKMEKLIVAMQDPDT-GIKMRIQRFLVTPIPHAMTGNDIFEWLLQNLSTSEE-ESFHLGDLIIKYGYIYPLKDPKNLVLRVD-DSPY  102
RGS11_M.mordax_39562-1-2                    -----------------------------MTSSKKVGCLVRRMQDTHT-GVPLLTRHWRTIAVPNAGIGHNITDWILRNLKITLE-ETFNLCNLLVRYGYLYPVYPATHLGFHL--NKLY  87
RGS11_G.australis_47497-1-2                 -----------------------------MASSEKVGCLICRMQDTHT-GVPLLTRHFLTFAIPNASIGHNISDWILRDLKTTQE-ETSNLCNLLVRYGYLYPVHPATQLGFHP--SKLY  87
RGS11-Partial-Trimmed_P.marinus_S4RW55      -----------------------------------VECLVCRMQDKQT-GVPLLTRHLITFAIPNASIGYNIADWMLRDMKTTQE-ETYNLCNLLVKYGYLYPVHPAAHLGFHP--SKLY  81
RGS11-Partial-Trimmed_L._camtschaticum_JL5627 ------------------------------------------MQDKQT-GVPLLTRHFITFAIPNASIGYNIADWMLRDMKITQE-ETSNLCNLLVKYGYLYPVRPAARLGFHP--SKLY  74
RGS11_E.cirrhatus_136920-1-2                -----------------------------MSGFEKVEMLVGAMQDPEM-GVNMFDQRLLLTVIPHAVRGHEILTWIIRNLKTSDE-EALNLSNLLVRTGYVYPLLEPANFSLQPD-DSIY  88
RGS_S.kowalevskii_XP-006813443              MVGMSEVGGDSGMV--GECGVGGENEMVGVSEVGGENKIVGVSEVGGE-SRISLIQLYVNDSIFVFITGYDLIEWLMENLDIQDSIEALHLANLFCQYGYIFPVQDIKNLVVKDD-STLY  116
RGS-Partial_S.purpuratus_XP-011673106       MT---------------------------------ASQVV--------------------------------------------------------------------------------  7
RGS6_Human_NP-001191345                     MAQGSG-DQRAVGV--AD----PEESSPNMIVYCKIEDIITKMQDDKTGGVPIRTVKSFLSKIPSVVTGTDIVQWLMKNLSIEDPVEAIHLGSLIAAQGYIFPISD-HVLTMKDD-GTFY  111
RGS7_Human_NP-002915                        MAQGNNYGQTSNGV--AD-------ESPNMLVYRKMEDVIARMQDEKN-GIPIRTVKSFLSKIPSVFSGSDIVQWLIKNLTIEDPVEALHLGTLMAAHGYFFPISD-HVLTLKDD-GTFY  108
                                            1.......10........20........30........40........50........60........70........80........90.......100.......110.......120
```

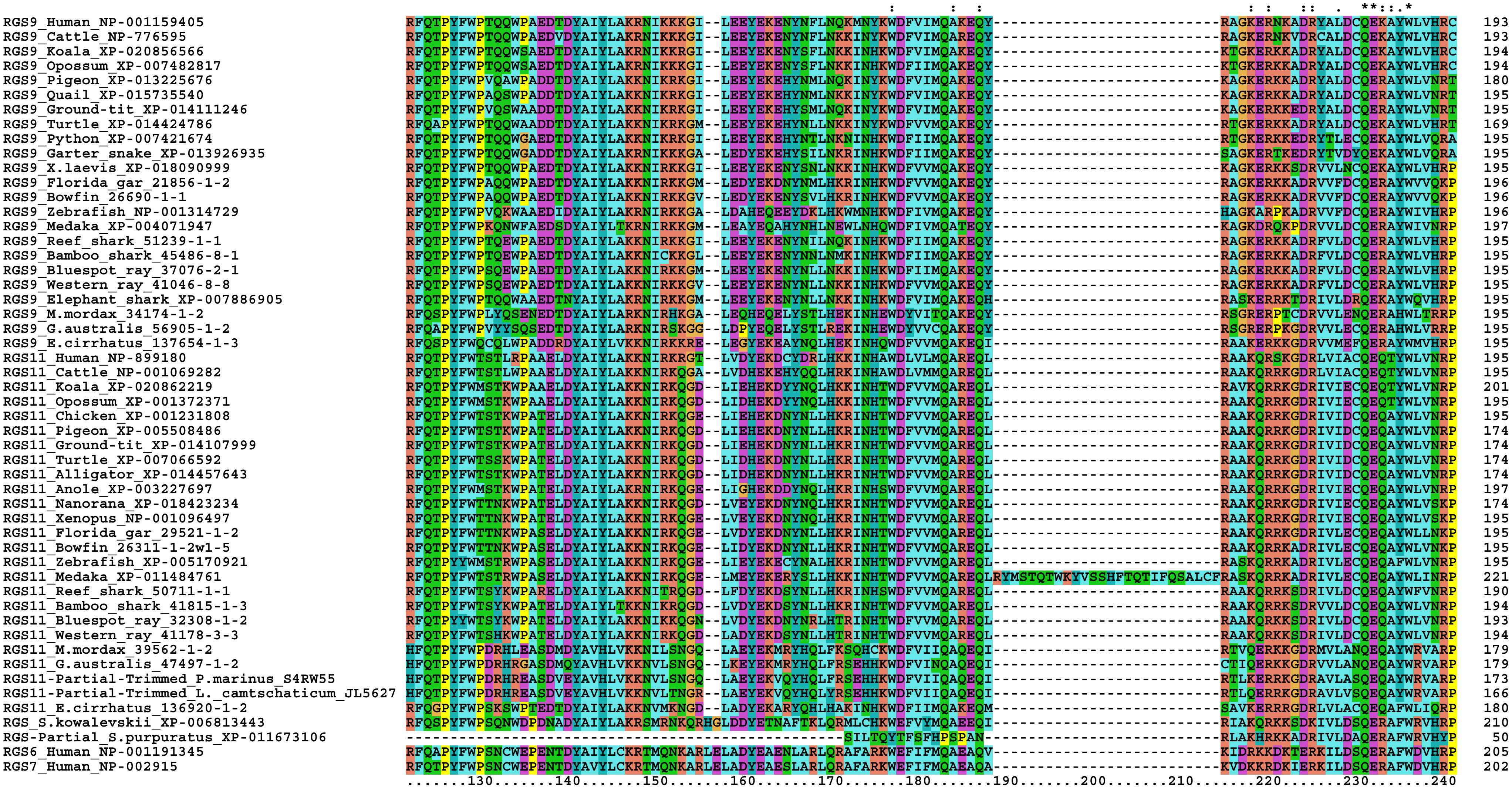
RGS9_Human_NP-001159405 193
RGS9_Cattle_NP-776595 193
RGS9_Koala_XP-020856566 194
RGS9_Opossum_XP-007482817 194
RGS9_Pigeon_XP-013225676 180
RGS9_Quail_XP-015735540 195
RGS9_Ground-tit_XP-014111246 195
RGS9_Turtle_XP-014424786 169
RGS9_Python_XP-007421674 195
RGS9_Garter_snake_XP-013926935 195
RGS9_X.laevis_XP-018090999 195
RGS9_Florida_gar_21856-1-2 196
RGS9_Bowfin_26690-1-1 196
RGS9_Zebrafish_NP-001314729 196
RGS9_Medaka_XP-004071947 197
RGS9_Reef_shark_51239-1-1 195
RGS9_Bamboo_shark_45486-8-1 195
RGS9_Bluespot_ray_37076-2-1 195
RGS9_Western_ray_41046-8-8 195
RGS9_Elephant_shark_XP-007886905 195
RGS9_M.mordax_34174-1-2 195
RGS9_G.australis_56905-1-2 195
RGS9_E.cirrhatus_137654-1-3 195
RGS11_Human_NP-899180 195
RGS11_Cattle_NP-001069282 195
RGS11_Koala_XP-020862219 201
RGS11_Opossum_XP-001372371 195
RGS11_Chicken_XP-001231808 195
RGS11_Pigeon_XP-005508486 174
RGS11_Ground-tit_XP-014107999 174
RGS11_Turtle_XP-007066592 174
RGS11_Alligator_XP-014457643 174
RGS11_Anole_XP-003227697 197
RGS11_Nanorana_XP-018423234 174
RGS11_Xenopus_NP-001096497 195
RGS11_Florida_gar_29521-1-2 195
RGS11_Bowfin_26311-1-2w1-5 195
RGS11_Zebrafish_XP-005170921 195
RGS11_Medaka_XP-011484761 221
RGS11_Reef_shark_50711-1-1 190
RGS11_Bamboo_shark_41815-1-3 194
RGS11_Bluespot_ray_32308-1-2 193
RGS11_Western_ray_41178-3-3 194
RGS11_M.mordax_39562-1-2 179
RGS11_G.australis_47497-1-2 179
RGS11-Partial-Trimmed_P.marinus_S4RW55 173
RGS11-Partial-Trimmed_L._camtschaticum_JL5627 166
RGS11_E.cirrhatus_136920-1-2 180
RGS_S.kowalevskii_XP-006813443 210
RGS-Partial_S.purpuratus_XP-011673106 50
RGS6_Human_NP-001191345 205
RGS7_Human_NP-002915 202
130 140 150 160 170 180 190 200 210 220 230 240

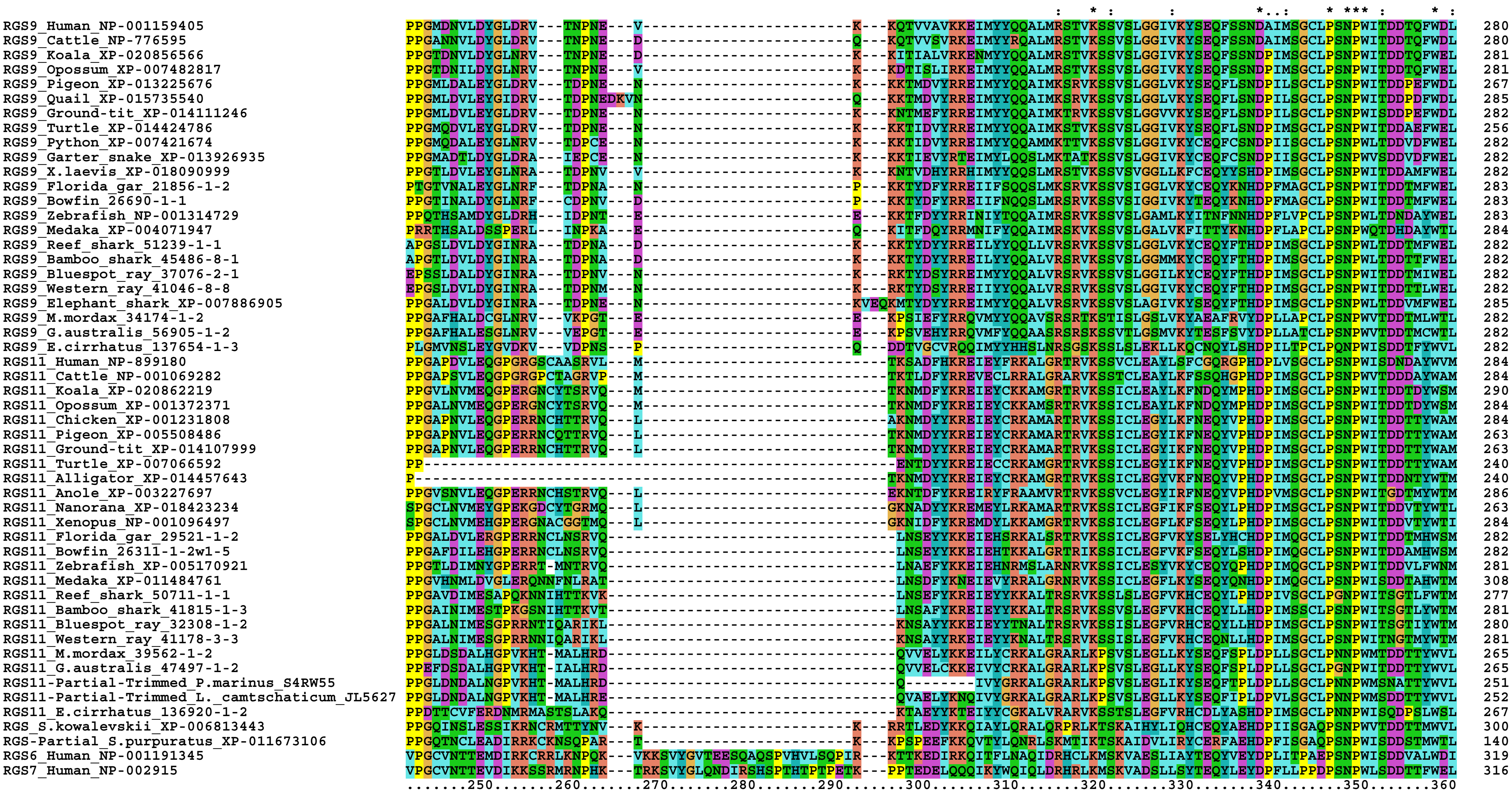
RGS9_Human_NP-001159405 280
RGS9_Cattle_NP-776595 280
RGS9_Koala_XP-020856566 281
RGS9_Opossum_XP-007482817 281
RGS9_Pigeon_XP-013225676 267
RGS9_Quail_XP-015735540 285
RGS9_Ground-tit_XP-014111246 282
RGS9_Turtle_XP-014424786 256
RGS9_Python_XP-007421674 282
RGS9_Garter_snake_XP-013926935 282
RGS9_X.laevis_XP-018090999 282
RGS9_Florida_gar_21856-1-2 283
RGS9_Bowfin_26690-1-1 283
RGS9_Zebrafish_NP-001314729 283
RGS9_Medaka_XP-004071947 284
RGS9_Reef_shark_51239-1-1 282
RGS9_Bamboo_shark_45486-8-1 282
RGS9_Bluespot_ray_37076-2-1 282
RGS9_Western_ray_41046-8-8 282
RGS9_Elephant_shark_XP-007886905 285
RGS9_M.mordax_34174-1-2 282
RGS9_G.australis_56905-1-2 282
RGS9_E.cirrhatus_137654-1-3 282
RGS11_Human_NP-899180 284
RGS11_Cattle_NP-001069282 284
RGS11_Koala_XP-020862219 290
RGS11_Opossum_XP-001372371 284
RGS11_Chicken_XP-001231808 284
RGS11_Pigeon_XP-005508486 263
RGS11_Ground-tit_XP-014107999 263
RGS11_Turtle_XP-007066592 240
RGS11_Alligator_XP-014457643 240
RGS11_Anole_XP-003227697 286
RGS11_Nanorana_XP-018423234 263
RGS11_Xenopus_NP-001096497 284
RGS11_Florida_gar_29521-1-2 282
RGS11_Bowfin_26311-1-2w1-5 282
RGS11_Zebrafish_XP-005170921 281
RGS11_Medaka_XP-011484761 308
RGS11_Reef_shark_50711-1-1 277
RGS11_Bamboo_shark_41815-1-3 281
RGS11_Bluespot_ray_32308-1-2 280
RGS11_Western_ray_41178-3-3 281
RGS11_M.mordax_39562-1-2 265
RGS11_G.australis_47497-1-2 265
RGS11-Partial-Trimmed_P.marinus_S4RW55 251
RGS11-Partial-Trimmed_L._camtschaticum_JL5627 252
RGS11_E.cirrhatus_136920-1-2 267
RGS_S.kowalevskii_XP-006813443 300
RGS-Partial_S.purpuratus_XP-011673106 140
RGS6_Human_NP-001191345 319
RGS7_Human_NP-002915 316
250 260 270 280 290 300 310 320 330 340 350 360

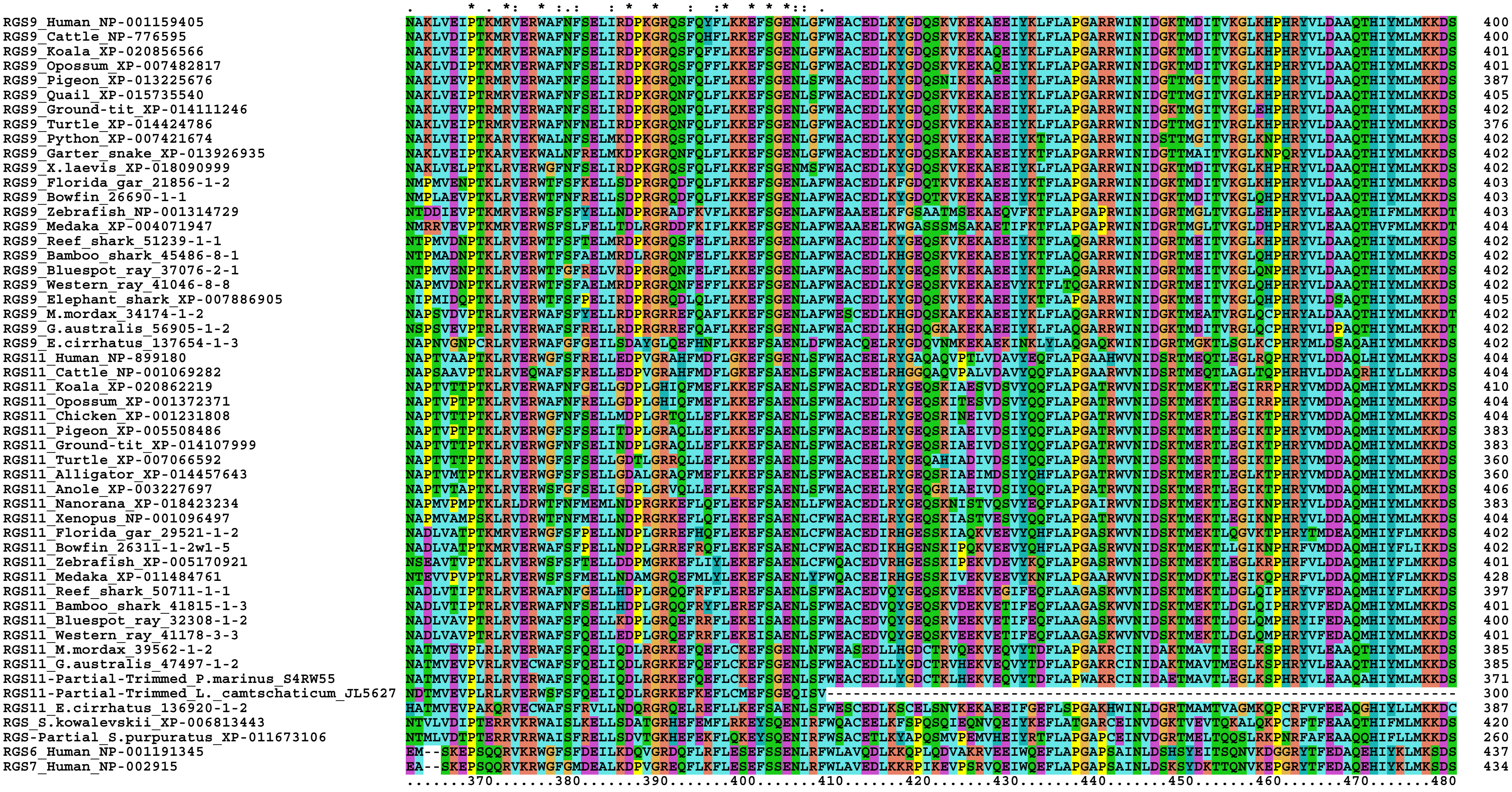

RGS9_Human_NP-001159405 400
RGS9_Cattle_NP-776595 400
RGS9_Koala_XP-020856566 401
RGS9_Opossum_XP-007482817 401
RGS9_Pigeon_XP-013225676 387
RGS9_Quail_XP-015735540 405
RGS9_Ground-tit_XP-014111246 402
RGS9_Turtle_XP-014424786 376
RGS9_Python_XP-007421674 402
RGS9_Garter_snake_XP-013926935 402
RGS9_X.laevis_XP-018090999 402
RGS9_Florida_gar_21856-1-2 403
RGS9_Bowfin_26690-1-1 403
RGS9_Zebrafish_NP-001314729 403
RGS9_Medaka_XP-004071947 404
RGS9_Reef_shark_51239-1-1 402
RGS9_Bamboo_shark_45486-8-1 402
RGS9_Bluespot_ray_37076-2-1 402
RGS9_Western_ray_41046-8-8 402
RGS9_Elephant_shark_XP-007886905 405
RGS9_M.mordax_34174-1-2 402
RGS9_G.australis_56905-1-2 402
RGS9_E.cirrhatus_137654-1-3 402
RGS11_Human_NP-899180 404
RGS11_Cattle_NP-001069282 404
RGS11_Koala_XP-020862219 410
RGS11_Opossum_XP-001372371 404
RGS11_Chicken_XP-001231808 404
RGS11_Pigeon_XP-005508486 383
RGS11_Ground-tit_XP-014107999 383
RGS11_Turtle_XP-007066592 360
RGS11_Alligator_XP-014457643 360
RGS11_Anole_XP-003227697 406
RGS11_Nanorana_XP-018423234 383
RGS11_Xenopus_NP-001096497 404
RGS11_Florida_gar_29521-1-2 402
RGS11_Bowfin_26311-1-2w1-5 402
RGS11_Zebrafish_XP-005170921 401
RGS11_Medaka_XP-011484761 428
RGS11_Reef_shark_50711-1-1 397
RGS11_Bamboo_shark_41815-1-3 401
RGS11_Bluespot_ray_32308-1-2 400
RGS11_Western_ray_41178-3-3 401
RGS11_M.mordax_39562-1-2 385
RGS11_G.australis_47497-1-2 385
RGS11-Partial-Trimmed_P.marinus_S4RW55 371
RGS11-Partial-Trimmed_L._camtschaticum_JL5627 300
RGS11_E.cirrhatus_136920-1-2 387
RGS_S.kowalevskii_XP-006813443 420
RGS-Partial_S.purpuratus_XP-011673106 260
RGS6_Human_NP-001191345 437
RGS7_Human_NP-002915 434
370 380 390 400 410 420 430 440 450 460 470 480

```
RGS9_Human_NP-001159405                   YARYLKSPIYKDMLAKAIEPQETTK----KSSTLPFMRRHLRSSPSPVILR-----QL-EEEAKAREAANT---VDITQVM-SKLDRRSQL------------------------QKELPP  483
RGS9_Cattle_NP-776595                     YARYLKSPIYKEMLAKAIEPQGTTR----KSSSLPFMRRHLRSSPSPVILR-----QL-EEEAKAREAATT---VDITQVM-SKLDRRSQL------------------------RKEPPP  483
RGS9_Koala_XP-020856566                   YARYLKSPIYKEMLAKAIEPQETSR----RSSSLPFRRRHLRSSPSPVILR-----QL-EEEAKAREAANT---VDITQVM-SKLDRRSQL------------------------KKEIPP  484
RGS9_Opossum_XP-007482817                 YARYLKSPIYKEMLAKAIEPQETSK----R-SSLPFMRRHLRSSPSPVILR-----QL-EEEAKAREAANT---VDITQVM-SKLDRRSQL------------------------KKEIPP  483
RGS9_Pigeon_XP-013225676                  YGRYLKSPIYKEMLAKAVVPQETVK----KSSNFPFGRRHLRSSPSPVILR-----QQ-EEEAKAKEAATT---VDITQVM-SKLDRRSQL------------------------QKD-PP  469
RGS9_Quail_XP-015735540                   YGRYLKSPVYKEMLAKAVVPQETVK----KSSSFPFGRRHLRSSPSPIILR-----QQ-EEEAKAKEAAAT---VDITQVM-SKLDRRSQL------------------------KQE-PP  487
RGS9_Ground-tit_XP-014111246              YGRYLKSPIYKEMLAKAVVPQEGVK----KSTGL-FGRRHLRSSPSPIILR-----QQ-EEEAKAKEAANT---VDITQVM-SKLDRRNQP------------------------QKEAPP  484
RGS9_Turtle_XP-014424786                  YGRYLKSPIYKEMLAKAIVPQETVK----KSSSFPFARRHLRSSPSPVILR-----QQ-EEEAKAKEAATN---VDITQVM-SKLDRRSQL------------------------KQE-PP  458
RGS9_Python_XP-007421674                  YGRYLKSPIYKEMLSKAITPQEAVK----KSSNNPFVRKHLRSSPSPILLR-----QL-EEEARAKEAAAN---VDITQVM-SKLDRRSQL------------------------RQEAPP  485
RGS9_Garter_snake_XP-013926935            YGRYLKSPVYKEMLSKAIVPQEVVK----KSSYNPFARKHLRSSPSPILLR-----QL-EEEAKAKEAATN---VDITQVM-SKLDRRSQL------------------------KQEAPP  485
RGS9_X.laevis_XP-018090999                YARYLKSPIYKEILAKAVVPQETKK------SNFPFMRRHRRSSPSPVILR-----QL-EEEARAKEAANT---VDITQVM-SKLDRRNQL------------------------KQETPK  483
RGS9_Florida_gar_21856-1-2                FGRYMKSPIFKETMKKAIVPDETKL----NEGKFGFNRKHRRSSPSPVILR-----QL-EEEAKAKAAASAP--VDITQVM-SKLDRRNQL------------------------KQEPPQ  487
RGS9_Bowfin_26690-1-1                     FGRYMKSPIFKDTMKKAIIPEETKV----SESKFGFSKKHRRSSPSPVILR-----QL-EEEAKAKAAANAP--VDITQVM-SKLDRRNQL------------------------KQEPAK  487
RGS9_Zebrafish_NP-001314729               FYRYLKSPVYKDIQKKAISPAPHNF----TEAQLEQNMRNRRPSISPIITY-----QK-EQEEKAKAAAAAGP-VDIKKLMASKLDRK---------------------------------  480
RGS9_Medaka_XP-004071947                  FFRYLKSPTYKDIQKKALNPEAHNF----SPAQLEQNAQNRNPGIDPIILW-----QQ-DEEEKAKAAAASAP-VDVKAMM-SKIDRKK--------------------------------  481
RGS9_Reef_shark_51239-1-1                 YPRFLRSPIYKETEGKALIPEETK-----QKSNFPFMKRHRRSSPSPVILR-----QL-ELEAKAKAAASGTKVVDITQVM-SKLDRRNQL------------------------KQEPPK  487
RGS9_Bamboo_shark_45486-8-1               YPRFLRSPIYKEMQSKALIPEETK-----PKSNFPFMKRHRRSSPSPVILR-----QL-EQEARAKAAASGPKVVDITQVM-SKLDRRNQL------------------------KQEPSK  487
RGS9_Bluespot_ray_37076-2-1               YPRFLKSPIYKETQNKALVPEETK-----QKSNFPFMRRQRRSSPSPVVLR-----QL-EEEARARAAASN-KVVDITQVM-SKLDRRHQL------------------------KQEPPR  486
RGS9_Western_ray_41046-8-8                YPRFLKSPIYKETQNKALVPEETK-----QKSNFPFMRRQRRSSPSPVVLR-----QL-EEEARARAAAST-KVVDITQVM-SKLDRRHQL------------------------KQEPPK  486
RGS9_Elephant_shark_XP-007886905          YPRYLKSPIYKETQAKALIPDQTK-----QKS-FPFMKRHRRSSPSPVLLR-----QL-EEEARARAAASGPKILDITQVM-SKLDRRNQL------------------------KPEPPK  489
RGS9_M.mordax_34174-1-2                   FYRYLKSDLYKKLLAQAIVPRETDK------S----ARFARHTSPSPVSLR-----AE-QEEEARRAAAAGPPPADLTQVL-SQLDRRNRP------------------------AK----  478
RGS9_G.australis_56905-1-2                FYRYLKSDLYKSLLARAVVPRETDK------S----ARFPRHASPSPLVIR-----AA-EEEEEARKAAAAGPPTDLTQVM-SKLDRRNQP------------------------AK----  478
RGS9_E.cirrhatus_137654-1-3               YPRYLKCDLYKNLLINAVVPQETKI------STLPFVHPKRHSNPSPVVLR-----NE-EDTAPLIPLPSASEVSGFSSIF-SPRIYRGPP------------------------AVGPGQ  486
RGS11_Human_NP-899180                     YPRFLKSDMYKALLAEAGIPLEMKR------RVFPFTWRPRHSSPSPAL-LPTPVEPTAACGPGGGD-------------G-VA-------------------------------------  467
RGS11_Cattle_NP-001069282                 YPRFLKSDLYRDLLAEAVVPLETKR------RVFPFTRKPRHSSPSPAL-LP----ASASGEPVGGD-----------GDG-ET-------------------------------------  465
RGS11_Koala_XP-020862219                  YPRFLKSDMYKTLLAEAVIPPETKK------RVFPFMRKQRHSSPSPAL-LL----PS-EQEPKGEDKNKALASAASAGEG-EG-------------------------------------  481
RGS11_Opossum_XP-001372371                YPRFLKSDMYKTLLAEAVIPPETKK------RVFPFMRKQRHSSPSPAL-FL----PSGEQEPKGDDKGKALAPGTSAGEG-EV-------------------------------------  476
RGS11_Chicken_XP-001231808                YPRFLKSELYRNLLAEAVIPPETKK------RVFPFMRKQRHSSPSPAL-LL----PAGDTEAKGEEKSRSPAA--VPEE--ES-------------------------------------  473
RGS11_Pigeon_XP-005508486                 YPRFLKSELYRNLLAEAVIPPETKK------RVFPFMRKQRHSSPSPAL-LL----PAGDTE----EKSRGPTASPVPEE--ES-------------------------------------  450
RGS11_Ground-tit_XP-014107999             YPRFLKSDLYKNLLAEAIIPPETKK------RVFPFMRKQRHSSPSPAL-LL----PAGDAE----ERSRSPSTAAVPEE--ES-------------------------------------  450
RGS11_Turtle_XP-007066592                 YPRFLKSELYRSLLAEAIVPPETKK------RVFPFMRKQRHSSPSPAL-LL----PTGEPEAKAEDQSKAVA---VPME---S-------------------------------------  427
RGS11_Alligator_XP-014457643              YPRFLKSDLYKSLLAEAIIPPETKK------RVFPFMRKQRHSSPSPALLLL----PGGEPEAKAEDKSRTPVAAPVPEE--ES-------------------------------------  432
RGS11_Anole_XP-003227697                  YPRFIKSDLYKNLLAEAIIPPETKR------RVFPFMRKPRHASPSPALQQL----PPGDSEGRAEPEQ---------PEG-GT-------------------------------------  470
RGS11_Nanorana_XP-018423234               YPRYLKSEMYKNLLGQAFIPPETKK------GVFPFMRRQRHSSPSPAF-LF----PPGDQESKADGDSRVNSPAAMSQE-----------------------------------------  452
RGS11_Xenopus_NP-001096497                YPRYLKSDMYKNLLGQAFIPPETKK------GVFPFMRRQRHSSPSPAF-LL----PQGEQEGRTEP----LTPAALSQP-----------------------------------------  469
RGS11_Florida_gar_29521-1-2               YPRFLKSDLYKNLLARAIVPQETKK------SVFPFMRRQRHSSPSPA-LVS----QPAEDDGKSKAA---PSSSGTVSQV-EE-------------------------------------  471
RGS11_Bowfin_26311-1-2w1-5                YPRYLKSDLYKNMLARAIVPQETKK------SVFPFMRRQRHSSPSPA-LVS----HPTEDDGKAKAS---P--AGTVSQV-EE-------------------------------------  469
RGS11_Zebrafish_XP-005170921              FPRYLKSDLYKSLLARAIVPQETKK------SVFPFMRRQRHSSPSPA-LVT----HTTEDDGKAKNSNSVKSNSGTASRL----------------------------------------  471
RGS11_Medaka_XP-011484761                 YPRYLKSDLYKNMLAKAIVPQETKK------SVFPFMRRQRHSSPSPAQAVA----QVTEEEGRHKGA---G-------------------------------------------------  487
RGS11_Reef_shark_50711-1-1                YPRYLKSDMYKNILARSVVPQETKK------CMIPFLRWYRNSSQSSA-QMF----YPKDGDGKGKDT--------TVSQV-QN-------------------------------------  461
RGS11_Bamboo_shark_41815-1-3              YPRYLKSDMYKNILARSIVPQETKR------SMVPFMRWHRYSNASSV-QMF----YPKDNDGKGKDA--------TVSQV-QN-------------------------------------  465
RGS11_Bluespot_ray_32308-1-2              YPRYLKSQMYKNLLESSIVPQESRK------SVFPFTKWHRQPNQNPS-QTP----RPKVSDGKDKAA-----PAGTVSQV-QN-------------------------------------  467
RGS11_Western_ray_41178-3-3               YPRYLKSQIYKNLLATSIVAQESKK------SAFPFTRWHRQPSQNPA-QTP----RP---DGKDKAA-----TSATVSQV-QN-------------------------------------  465
RGS11_M.mordax_39562-1-2                  YPRYLKSDLYKTMLARAIVASAVNA------HSH---------------------------------------------------------------------------------------  413
RGS11_G.australis_47497-1-2               YPRYLKSDLYKTMLAGAIVPCAVNA------HPLSGTRSL---------------------------------------------------------------------------------  419
RGS11-Partial-Trimmed_P.marinus_S4RW55    YPRYLKSDLYKTMLAGAIVP-----------------------------------------------------------------------------------------------------  391
RGS11-Partial-Trimmed_L._camtschaticum_JL5627 -------------------------------------------------------------------------------------------------------------------------  300
RGS11_E.cirrhatus_136920-1-2              YPRYLKSEMYKSLLERAIVPQDNKR------RIFPLMMRPRLSSPPIT-LQG----I----------------------------------------------------------------  433
RGS_S.kowalevskii_XP-006813443            YPRFLRSDAYKNLLAHSLQPSSKKKFFTFGNR-LKTAKVTPNASPQPLRRR-----GS-ANDMTHKAHNARHGLSLSTGNL-QDLDKSDEKINADFLYSDVYQGGESKENSPN-QSPTSN  531
RGS-Partial_S.purpuratus_XP-011673106     YQRFLKSDTYKGLLARAMQNGTKRKFFTFGGRFLRSAKVTPDVSPLGYPRR-----GSLGTDISDSLSDRPMTHSLSASNL-QD----------FELANMYNGTENGHKTEEVYTSVKS  363
RGS6_Human_NP-001191345                   YARFLRSNAYQDLLLAKKKPESEQG------RRTSLEKFTRSVGKSLAGKR-------------------------LTGLM-QS-------------------------------------  489
RGS7_Human_NP-002915                      YPRFIRSSAYQELLQAKKKSGNSMD------RRTSFEKFAQNVGKSLTSKR-------------------------LTSLA-QS-------------------------------------  486
                                          .......490.......500.......510.......520.......530.......540.......550.......560.......570.......580.......590.......600
```

```
RGS9_Human_NP-001159405                   -K--------------------------------------------------------------------------  484
RGS9_Cattle_NP-776595                     -K--------------------------------------------------------------------------  484
RGS9_Koala_XP-020856566                   -K--------------------------------------------------------------------------  485
RGS9_Opossum_XP-007482817                 -K--------------------------------------------------------------------------  484
RGS9_Pigeon_XP-013225676                  -K--------------------------------------------------------------------------  470
RGS9_Quail_XP-015735540                   -K--------------------------------------------------------------------------  488
RGS9_Ground-tit_XP-014111246              -K--------------------------------------------------------------------------  485
RGS9_Turtle_XP-014424786                  -K--------------------------------------------------------------------------  459
RGS9_Python_XP-007421674                  AN--------------------------------------------------------------------------  487
RGS9_Garter_snake_XP-013926935            AK--------------------------------------------------------------------------  487
RGS9_X.laevis_XP-018090999                P--------------------------------------------------------------------------T  485
RGS9_Florida_gar_21856-1-2                KT--------------------------------------------------------------------------  489
RGS9_Bowfin_26690-1-1                     ----------------------------------------------------------------------------  487
RGS9_Zebrafish_NP-001314729               ----------------------------------------------------------------------------  480
RGS9_Medaka_XP-004071947                  ----------------------------------------------------------------------------  481
RGS9_Reef_shark_51239-1-1                 QE--AQ----------------------------------------------------------------------  491
RGS9_Bamboo_shark_45486-8-1               QE--A-----------------------------------------------------------------------  490
RGS9_Bluespot_ray_37076-2-1               PE--AQ----------------------------------------------------------------------  490
RGS9_Western_ray_41046-8-8                PE--AQ----------------------------------------------------------------------  490
RGS9_Elephant_shark_XP-007886905          EEPPKQ----------------------------------------------------------------------  495
RGS9_M.mordax_34174-1-2                   ----------------------------------------------------------------------------  478
RGS9_G.australis_56905-1-2                ----------------------------------------------------------------------------  478
RGS9_E.cirrhatus_137654-1-3               SNAISCNPMQN----------------------------------------------------------------G  498
RGS11_Human_NP-899180                     ----------------------------------------------------------------------------  467
RGS11_Cattle_NP-001069282                 ----------------------------------------------------------------------------  465
RGS11_Koala_XP-020862219                  ----------------------------------------------------------------------------  481
RGS11_Opossum_XP-001372371                ----------------------------------------------------------------------------  476
RGS11_Chicken_XP-001231808                ----------------------------------------------------------------------------  473
RGS11_Pigeon_XP-005508486                 ----------------------------------------------------------------------------  450
RGS11_Ground-tit_XP-014107999             ----------------------------------------------------------------------------  450
RGS11_Turtle_XP-007066592                 ----------------------------------------------------------------------------  427
RGS11_Alligator_XP-014457643              ----------------------------------------------------------------------------  432
RGS11_Anole_XP-003227697                  ----------------------------------------------------------------------------  470
RGS11_Nanorana_XP-018423234               ----------------------------------------------------------------------------  452
RGS11_Xenopus_NP-001096497                ----------------------------------------------------------------------------  469
RGS11_Florida_gar_29521-1-2               ----------------------------------------------------------------------------  471
RGS11_Bowfin_26311-1-2w1-5                ----------------------------------------------------------------------------  469
RGS11_Zebrafish_XP-005170921              ----------------------------------------------------------------------------  471
RGS11_Medaka_XP-011484761                 ----------------------------------------------------------------------------  487
RGS11_Reef_shark_50711-1-1                ----------------------------------------------------------------------------  461
RGS11_Bamboo_shark_41815-1-3              ----------------------------------------------------------------------------  465
RGS11_Bluespot_ray_32308-1-2              ----------------------------------------------------------------------------  467
RGS11_Western_ray_41178-3-3               ----------------------------------------------------------------------------  465
RGS11_M.mordax_39562-1-2                  ----------------------------------------------------------------------------  413
RGS11_G.australis_47497-1-2               ----------------------------------------------------------------------------  419
RGS11-Partial-Trimmed_P.marinus_S4RW55    ----------------------------------------------------------------------------  391
RGS11-Partial-Trimmed_L._camtschaticum_JL5627 ------------------------------------------------------------------------  300
RGS11_E.cirrhatus_136920-1-2              ----------------------------------------------------------------------------  433
RGS_S.kowalevskii_XP-006813443            KKFIIGSPITSRKKVNFTTQEHVNMSLEVPKRYPVNSPDSPEVDTNSKPLSFPVPSKKSLITPWEEQDIPTDSSLN  607
RGS-Partial_S.purpuratus_XP-011673106     SRFASLQPMNSTNLKRHGSLG------------------------------------------------------G  385
RGS6_Human_NP-001191345                   ---------------------------------------------------------------------------S  490
RGS7_Human_NP-002915                      ---------------------------------------------------------------------------Y  487
                                          .......610.......620.......630.......640.......650.......660.......670......
```

```
                                                                                     . :    : *::  *  *   ** :* :** **:***:  *::: : *  : :*.**
Gb5L_Human_NP-057278                  MCDQTFLVNVFGSCDKCFKQRALRPVFKKSQQLSYCSTCAEIMATEG-LHENETLASLKSEAESLKGKLEEERAKLHDVELHQVAERVEALGQFVMKTRR   99
Gb5L_Cattle_XP-010807658              M-DQTFLVNVFGSCDRCFKQRALRPVFKKSEQLNYCSLCVEIMAADG-LHENETLASLKSEAESLKGKLEEERAKLHDVELHQVAERVEALGQFVMKTRR   98
Gb5L_Opossum_XP-007480030             MCDQTFLVNVFGSCDKCFKQRALRPVFIRSQQLSYCSVCAALMAAEG-LHESETLASLKSEAESLKSRLEEERAKLHDVELHQVAERVEALGQFVMKTRR   99
Gb5L_Ground_tit_XP-005521542          M-DQTFLAIVFGPCDKCSKEKPLRPVSVKSEQRSYCSLCVEMMATEG-LHENETLASLKSEAESLKGKLEEERAKLHDVELHQVAERVEALGQFVMKTRR   98
Gb5L_Crow_XP-008638229                M-DQTFLAIVFGPCDKCSKDKPLRPVSVKSEQRSYCSLCVAMMATEG-LHENETLASLKSEAESLKGKLEEERAKLHDVEVHQVAERVEALGQFVMKTRR   98
Gb5L_Falcon_XP-005241495              MCDQTFLAIVFGPCDKCSKEKPLRAVYIKSEQLSYCSLCVEMMATEG-LHENETLASLKNEAESLKGKLEEERAKLHDVELHQVAERVEALGQFVMKTRR   99
Gb5L_Turtle_XP-007056427              MCDQTFLAIVFGPCDKCSTEKPLRATYIKSEQLNYCSLCVEMMATEG-LHENETLASLKSEAESLKGRLEEERAKLHDVELHQVAERVEALGQFVMKTRR   99
Gb5L_Alligator_XP-019333396           MCDQTFLATVFGPCDKCSNLKPLRAIYIKSSQLDYCSLCVEMMATEG-LHENETLASLKSEAESLKGKLEEERAKLHDVELHQVADRVEALGQFVMKTRR   99
Gb5L_Xenopus_XP-012814636             MCDQTFLTVLFGPCGKCKRERPLRTIYVKAEPVTYCSLCVEMMATEG-LQENETLASLKNEAESLKEKLEEERAKLHDVELHQVAERVESLGQFVMKTRR   99
Gb5L_Nanorana_XP-018431551            MCDQTFLVVLFGPCAKCTRERPLRTIYLKAEPVTLCSLCVELMATEG-LQENETLASLKNEAEGLKGKLEEERAKLHDVELHQVAERVESLGQFVMKTRR   99
Gb5L_Coelacanth_XP-014351988          MCDQTFLAVAFGPCEKCQQEKPLRNIYIKTEQVNYCTLCVELMATEG-LQENETLASLKNEAESLKGKLEEERAKLHDVELHQVADRVDGLGQFVMKTRR   99
Gb5L_Florida_gar_16504-1-1            MCDQTFLAINFGPCDKCQKTKPLMNIYIKSEPINYCALCVELMATEG-LQESETLASLKNESETLKSKLEEERAKLHDVELHQVADRVEALGQFVMKTRR   99
Gb5L_Bowfin_22627-1-1                 MCDQTFLATNFGPCEKCGKTKPLMNIYIKTDPINYCSLCVELMATEG-LQENETLASLKNESETLKSKLEEERAKLHDVELHQVADRVEALGQFVMKTRR   99
Gb5L_Zebrafish_NP-957040              MCDQTFLAITFGPCDKCSENKPLMNIYLKNEPINYCSLCVEMMACQG-LAKGETVQSLKAESESLKAKLEEERAKLHDVELHQVAEKMEALGQFVMKTRR   99
Gb5L_Medaka_XP-011491247              MCDQTFMAATFGPCDSCGKASPLMNIYIKSEAINYCSFCVEMMACQQ-LQKGETLQSLKKESDSLKKKLEEERGKLNDVELHQVAEKVEVLSSLSIKTRR   99
Gb5L_Reef_shark_40872-1-1             MCDQTFAAATFGPCEKCQEVGPLRNLYINEEQINYCSLCVEMMATEG-LQENETVSSLRDEAETLKGKLEEERAKLHDVELHQVAERTEALGQFVMKTRR   99
Gb5L_Bamboo_shark_37645-2-1           MCDQTFAAATFGPCEKCQEVGPLRNLYIDTEQINFCSLCVEMMATEG-LQENESVSSLREEAETLKGKLEEERAKLHDVELHQVAERTEALGQFVMKTRR   99
Gb5L_Bluespot_ray_28709-1-1           MCDQTFAAAVHGPCERCQEVGPLRNLYIKDEQINVCSLCVEMMATEG-LQENETVSSLRNEAEALKGKLEEERAKLHDVELHQVAERTEALGQFVMKTRR   99
Gb5L_Western_ray_37263-2-2            MCDQTFAAAVYGPCEKCQEIGPLRNLYINAEQINYCSLCVEMMATEG-LQENETVSTLRNEAEALKGKLEEERAKLHDVELHQVAERTEALGQFVMKTRR   99
Gb5S_Elephant_shark_XP-007904318      ------------------------------------------MATEA-LQENETVASLKDEAESLKSKLEEERAKLHDVELHQVAERAEALGQFVMKTRR   57
Gb5L_M.mordax_41947-2-2               MCDQTFLTTCFGQCDACCKERPLRSVLSKAQPRNVCTICAELMASEG-LQENETIDGLQGEAENLKIKLEEERAKLHDVELHQVAERVEALGQFVMKTRR   99
Gb5L_G.australis_57438-2-2            MCDQTFLVICFGKCDVCSNERPLRGVFIQAQPRNVCTSCAEMMASEG-LQENETIDGLQGEAENLKIKLEEERAKLHDVELHQVAERVDALGQFVMKTRR   99
Gb5L-Partial_P.marinus_S4RZE3         MCDQTFLVVCFGQCDLCFKERPLRGVLINAQSQNVCTSCAELMASEG-LQENETIDGLQGEAENLKMKLEEERAKLHDVELHQVAERVEALGQFVMKTRR   99
Gb5L_E.cirrhatus_130391-1-2           MSDQIFSAVNFGPCESCECVHALQEVWSHGKAYSLCTECKQLMASEV-LQEGETLSVLQQEAAALKARLEDERAKLHDVELRQVAERVEPLGQFVMKPRR   99
Gb5_S.kowalevskii_XP-002740524        -----------------------------------------MATTDA-AMKEESISGLSRELETLKAKLEEERHKLNDVELRLVSQRLELLPQFNMKPRR   58
Gb5_S.purpuratus_XP-792481            -----------------------------------------MAVTDGNAVKEETLTALTREAENLKQKLLEERHKLNDVEMYTVAQRLEPLPPLNLKPRR   59
                                      1.......10........20........30........40........50........60........70........80........90.......100

                                       **** .***.:**.:*:*::*:****** ****.:* ****.:*:* ******:*****  :***  *******:  *: :::   ::**::*****.*
Gb5L_Human_NP-057278                  TLKGHGNKVLCMDWCKDKRRIVSSSQDGKVIVWDSFTTNKEHAVTMPCTWVMACAYAPSGCAIACG--GLDNKCSVYPLTFDKNENMAAKKKSVAMHTNY  197
Gb5L_Cattle_XP-010807658              TLKGHGNKVLCMDWCKDKRRLVSSSQDGKVIVWDSFTTNKEHAVTMPCTWVMACAYAPSGCAIACG--GLDNKCSVYPLTFDKNENMAAKKKSVAMHTNY  196
Gb5L_Opossum_XP-007480030             TLKGHGNKVLCMDWCKDKRRIVSSSQDGKVIVWDSFTTNKEHAVTMPCTWVMACAYAPSGCAIACG--GLDNKCSVYPLTFDKNENMAAKKKSVAMHTNY  197
Gb5L_Ground_tit_XP-005521542          TLKGHGNKVLCMDWCKDKRRIVSSSQDGKVIVWDSFTTNKEHAVTMPCTWVMACAYAPSGCAIACG--GLDNKCSVYPLTFEKNENMAAKKKSVAMHTNY  196
Gb5L_Crow_XP-008638229                TLKGHGNKVLCMDWCKDKRRIVSSSQDGKVIVWDSFTTNKEHAVTMPCTWVMACAYAPSGCAIACG--GLDNKCSVYPLTFEKNENMAAKKKSVAMHTNY  196
Gb5L_Falcon_XP-005241495              TLKGHGNKVLCMDWCKDKRRIVSSSQDGKVIVWDSFTTNKEHAVTMPCTWVMACAYAPSGCAIACG--GLDNKCSVYPLTFDKNENMAAKKKSVAMHTNY  197
Gb5L_Turtle_XP-007056427              TLKGHGNKVLCMDWCKDKRRIVSSSQDGKVIVWDSFTTNKEHAVTMPCTWVMACAYAPSGCAIACG--GLDNKCSVYPLTFDKNENMAAKKKSVAMHTNY  197
Gb5L_Alligator_XP-019333396           TLKGHGNKVLCMDWCKDKRRIVSSSQDGKVIVWDSFTTNKEHAVTMPCTWVMACAYAPSGCAIACG--GLDNKCSVYPLTFDKNENMAAKKKSVAMHTNY  197
Gb5L_Xenopus_XP-012814636             TLKGHGNKVLCMDWCKDKRRIVSSSQDGKVIVWDAFTTNKEHAVTMPCTWVMACAYAPSGCAIACG--GLDNKCSVYPLTFDKNENMAAKKKSVAMHTNY  197
Gb5L_Nanorana_XP-018431551            TLKGHGNKVLCMDWCKDKRRIVSSSQDGKVIVWDAFTTNKEHAVTMPCTWVMACAYAPSGCAIACG--GLDNKCSVYPLTFDKNENMASKKKSVAMHTNY  197
Gb5L_Coelacanth_XP-014351988          TLKGHGNKVLCMDWCKDKRRIVSSSQDGKVIVWDAFTTNKEHAVTMPCTWVMACAYAPSGCAIACG--GLDNKCSVYPLTFDKNENMAAKKKSVAMHTNY  197
Gb5L_Florida_gar_16504-1-1            TLKGHGNKVLCMDWCKDKRRIVSSSQDGKVIVWDAFTTNKEHAVTMPCTWVMACAYAPSGCAVACG--GLDNKCSVYPLTFDKNENMASKKKSVAMHTNY  197
Gb5L_Bowfin_22627-1-1                 TLKGHGNKVLCMDWCKDKRRIVSSSQDGKVIVWDAFTTNKEHAVTMPCTWVMACAYAPSGCAIACG--GLDNKCSVYPLTFDKNENMAAKKKSVAMHTNY  197
Gb5L_Zebrafish_NP-957040              TLKGHGNKVLCMDWCRDKRRIVSSSQDGKVIVWDAYTTNKEHAVTMPCTWVMACAYAPSGCAVACG--GLDNKCSVYPLSLDKNENLASKKKSVAMHTNY  197
Gb5L_Medaka_XP-011491247              VLKGHGNKVLCVDWCKDKRRLVSSSQDGKAIVWDAFTLNKEHGVTLPCTWVMACAYAPSGCAVACG--GLDNKCSVVPLSLDKNENLAAKKKSVAMHTNY  197
Gb5L_Reef_shark_40872-1-1             TLKGHGNKVLCMDWCRDKRRIVSSSQDGKVIVWDAFTTNKEHAVTMPCTWVMACAYAPSGCAIACG--GLDNKCSVYPLTFDKNESMAAKKKSVAMHTNY  197
Gb5L_Bamboo_shark_37645-2-1           TLKGHGNKVLCMDWCRDKRRIVSSSQDGKVIVWDAFTTNKEHAVTMPCTWVMACAYAPSGCAVACG--GLDNKCSVYPLTFDKNESMAAKKKSVAMHTNY  197
Gb5L_Bluespot_ray_28709-1-1           TLKGHGNKVLCIDWCRDKRRIVSSSQDGKVIVWDAFTTNKEHAVTMPCTWVMACAYAPSGCAIACG--GLDNKCSVYPLTFDKNESMAAKKKSVAMHTNY  197
Gb5L_Western_ray_37263-2-2            TLKGHGNKVLCMDWCRDKRRIVSSSQDGKVIVWDAFTTNKEHAVTMPCTWVMACAYAPSGCAIACG--GLDNKCSVYPLTFDKNETMAAKKKSVAMHTNY  197
Gb5S_Elephant_shark_XP-007904318      TLKGHGNKVLCMDWCRDKRRIVSSSQDGKVIVWDAFTTNKEHAVTMPCTWVMACAYAPSGCAVACG--GLDNKCSVYPLTFDKNESMAAKKKSVAMHTNY  155
Gb5L_M.mordax_41947-2-2               TLKGHGNKVLCMDWCKDKRRLVSSSQDGKVIVWDAFTTNKEHAVTMPCTWVMACAYAPSGCAIACG--GLDNKCSVYPLSTDKNENLAAKKKSVAMHTNY  197
Gb5L_G.australis_57438-2-2            TLKGHGNKVLCMDWCKDKRRLVSSSQDGKVIVWDAFTTNKEHAVTMPCTWVMACAYAPSGCAIACG--GLDNKCSVYPLSMDKNENLAAKKKSVAMHTNY  197
Gb5L-Partial_P.marinus_S4RZE3         TLKGHGNKVLCMDWCKDKRRLVSSSQDGKVIVWDAFTTNKEHAVTMPCTWVMACAYAPSGCAIACGDRGLDNKCSVYPLSMDKNENLAAKKKSVAMHTNY  199
Gb5L_E.cirrhatus_130391-1-2           TLKGHGNKVLCMDWCRDKRRLVSSSQDGKVIVWDGFTCNKEHAVTMPCTWVMACAYAPSGCAIACG--GLDNKCSVYPLSFDKNENMAAKKKSVAMHTNY  197
Gb5_S.kowalevskii_XP-002740524        VLKGHQGKVLAMDWSQDKRHVVSSSQDGKMIVWDAFTTNKEHAVTMPTTWVMACSYAPSGTMVACG--GLDNKCSIYQLSLDEDV--ASKKRTVAMHTSY  154
Gb5_S.purpuratus_XP-792481            ILKGHQGKVLCLDWARDQRHMVTSSQDGKVIVWDAFTTNKEHAITMPTTWVMACAYAPSGTMVACG--GLDNKCSVYQLTQEEDLS-SSKKRAVAMHTSY  156
                                      .......110.......120.......130.......140.......150.......160.......170.......180.......190.......200
```

```
                                  :* * * :** *:*
Gb5L_Human_NP-057278             LSACSFTNSDMQILTASGDGTCALWDVESGQLLQSFHGHGADVLCLDLAPSETGNTFVSGGCDKKAMVWDMRSGQCVQAFETHESDINSVRYYPSGDAFA  297
Gb5L_Cattle_XP-010807658         LSACSFTNSDMQILTASGDGTCALWDVESGQLLQSFHGHGADVLCLDLAPSETGNTFVSGGCDKKAMVWDMRSGQCVQAFETHESDINSVRYYPSGDAFA  296
Gb5L_Opossum_XP-007480030        LSACSFTNSDMQILTASGDGTCALWDVESGQLLQSFHGHGADVLCLDLAPSETGNTFVSGGCDKKAMVWDMRSGQCIQSFETHESDINSVRYYPSGDAFA  297
Gb5L_Ground_tit_XP-005521542     LSACSFTNSDMQILTASGDGTCALWDVESGQLLQSFHGHGADVLCLDLAPSETGNTFVSGGCDKKAMVWDMRSGQCVQSFETHDSDINSVRYYPSGDAFA  296
Gb5L_Crow_XP-008638229           LSACSFTNSDMQILTASGDGTCALWDVESGQLLQSFHGHGADVLCLDLAPSETGNTFVSGGCDKKAMVWDMRSGQCVQSFETHDSDINSVRYYPSGDAFA  296
Gb5L_Falcon_XP-005241495         LSACSFTNSDMQILTASGDGTCALWDVESGQLLQSFHGHGADVLCLDLAPSETGNTFVSGGCDKKAMVWDMRSGQCVQSFETHDSDINSVRYYPSGDAFA  297
Gb5L_Turtle_XP-007056427         LSACSFTNSDMQILTASGDGTCALWDVESGQLLQSFHGHGADVLCLDLAPSETGNTFVSGGCDKKAMVWDMRSGQCIQSFETHESDINSVRYYPSGDAFA  297
Gb5L_Alligator_XP-019333396      LSACSFTNSDMQILTASGDGTCALWDVESGQLLQSFHGHGADVLCLDLAPSETGNTFVSGGCDKKAMVWDMRSGQCIQSFETHESDINSVRYYPSGDAFA  297
Gb5L_Xenopus_XP-012814636        LSACSFTNSDMQILTASGDGTCALWDVESGQLLQSFHGHGADVLCLDLAPSETGNTFVSGGCDKKAMVWDMRTGQCIQSFETHESDINSVRYYPSGDAFA  297
Gb5L_Nanorana_XP-018431551       LSACSFTNSDMQILTASGDGTCALWDVESGQLLQSFHGHGADVLCLDLAPSETGNTFVSGGCDKKAMVWDMRTGQCIQSFETHESDINSVRYYPSGDAFA  297
Gb5L_Coelacanth_XP-014351988     LSSCSFTNSDMQILTASGDGTCALWDVESGQLLQSFHGHGADVLCLDLAPSETGNTFVSGGCDKKAMVWDMRSGQCIQSFETHESDINSVRYYPSGDAFA  297
Gb5L_Florida_gar_16504-1-1       LSACSFTNSDMQILTASGDGTCALWDVESGQLLQSFHGHAADVLSLDLAPSETGNTFVSGGCDKKAMVWDLRSGQCIQSFETHESDINSVRYYPSGDAFA  297
Gb5L_Bowfin_22627-1-1            LSACSFTNSDMQILTASGDGTCALWDVESGQLLQSFHGHAADVLCLDLAPSETGNTFVSGGCDKKAMVWDLRSGQCIQSFETHESDINSVRYYPSGDAFA  297
Gb5L_Zebrafish_NP-957040         LSSCSFTKSDMQILTSSGDGTCALWDVESGQLLQSFHGHSADVLSLDLAPSETGSTFVSGGCDKKANVWDMRSGQNVQSFETHDSDINSVKYYPSGDAFA  297
Gb5L_Medaka_XP-011491247         VSGCTFTNSDMQLLTSSGDGTCALWDVESGQLLQSFHGHTADVLSLDLAPSETGNTFVSGGCDMKANVWDMRSGQNIQSFESHESDINCVRYYPSGDAFA  297
Gb5L_Reef_shark_40872-1-1        LSACSFTNSDMQILTSSGDGTCALWDVESGQLLQSFHGHGADVLCLDLAPSETGHTFVSGGCDKKAMVWDMRSGQCIQSFETHESDINSVRYFPSGDAFA  297
Gb5L_Bamboo_shark_37645-2-1      LSACSFTNSDMQILTSSGDGTCALWDVESGQLLQSFHGHGADVLCLDLAPSETGHTFVSGGCDKKAMVWDMRSGQCIQSFETHESDINSVRYFPSGDAFA  297
Gb5L_Bluespot_ray_28709-1-1      LSACSFTNSDMQILTSSGDGTCALWDVESGQLLQSFHGHGADVLCLDLAPSETGHTFVSGGCDKKAMVWDMRSGQCIQSFETHESDINSVRYFPSGDAFA  297
Gb5L_Western_ray_37263-2-2       LSACSFTNSDMQILTSSGDGTCALWDVESGQLLQSFHGHGADVLCLDLAPSETGHTFVSGGCDKKAMVWDMRSGQCIQSFETHESDINSVRYFPSGDAFA  297
Gb5S_Elephant_shark_XP-007904318 LSACSFTNSDMQILTASGDGTCALWDVESGQLLQSFHGHGADVLCLDLAPSETGHTFVSGGCDKKAMVWDMRSGQCIQSFETHESDINSVRYYPSGDAFV  255
Gb5L_M.mordax_41947-2-2          LSACCFTNSDMQILTSSGDGTCALWDVESGQLLQSFHGHGADVLCLDLAPSETGNTFVSGGCDKKAMVWDMRTGQCVQCFETHESDINSVRYYPSGDAFA  297
Gb5L_G.australis_57438-2-2       LSACCFTNSDMQILTSSGDGTCALWDVESGQLLQSFHGHGADVLCLDLAPSETGNTFVSGGCDKKAMVWDMRTGQCVQCFETHESDINSVRYYPSGDAFA  297
Gb5L-Partial_P.marinus_S4RZE3    LSACCFTNSDMQVL--------------------------------------------------------------------------------------  213
Gb5L_E.cirrhatus_130391-1-2      LSACCFTNSDMQLLTASGDGTCALWDVESGQLLQSFHGHGADVLCLDLAPSETGNTFISGGCDKKALVWDIRTGQCVQCFETHESDINSVRFYPSGDAFA  297
Gb5_S.kowalevskii_XP-002740524   MSCCSFTNSDQQILTGSGDSTCALWDVESGQLLQSFHGHNADVMSLDLSPSESGNTFVSGGCDKQALVWDMRTGQCVQSFDGHESDINAVRFFPSGDAFA  254
Gb5_S.purpuratus_XP-792481       VSCCTFMNSDQQILTGSGDSTCALWDVESGQLLQSFHGHNADAMALDLSPSEACNTFVSGGCDKQALVWDMRTGQCVQSFEGHDSDINAVRFFPSGDGFA  256
                                 .......210.......220.......230.......240.......250.......260.......270.......280.......290.......300

Gb5L_Human_NP-057278             SGSDDATCRLYDLRADREVAIYSKESIIFGASSVDFSLSGRLLFAGYNDYTINVWDVLKGSRVSILFGHENRVSTLRVSPDGTAFCSGSWDHTLRVWA  395
Gb5L_Cattle_XP-010807658         SGSDDATCRLYDLRADREVAIYSKESIIFGASSVDFSLSGRLLFAGYNDYTINVWDVLKGSRVSILFGHENRVSTLRVSPDGTAFCSGSWDHTLRVWA  394
Gb5L_Opossum_XP-007480030        SGSDDATCRLYDLRADREVAIYSKESIIFGASSVDFSLSGRLLFAGYNDYTINVWDVLKGSRVSILFGHENRVSTLRVSPDGTAFCSGSWDHTLRVWA  395
Gb5L_Ground_tit_XP-005521542     SGSDDATCRLYDLRADREVAIYSKESIIFGASSVDFSLSGRLLFAGYNDYTINVWDVLKGARVSILFGHENRVSTLRVSPDGTAFCSGSWDHTLRIWA  394
Gb5L_Crow_XP-008638229           SGSDDATCRLYDLRADREVAIYSKESIIFGASSVDFSLSGRLLFAGYNDYTINVWDVLKGSRVSILFGHENRVSTLRVSPDGTAFCSGSWDHTLRIWA  394
Gb5L_Falcon_XP-005241495         SGSDDATCRLYDLRADREVAIYSKESIIFGASSVDFSLSGRLLFAGYNDYTINVWDVLKGSRVSILFGHENRVSTLRVSPDGTAFCSGSWDHTLRIWA  395
Gb5L_Turtle_XP-007056427         SGSDDATCRLYDLRADREVAIYSKESIIFGASSVDFSLSGRLLFGGYNDYTINVWDVLKGSRVSILFGHENRVSTLRVSPDGTAFCSGSWDHTLRIWA  395
Gb5L_Alligator_XP-019333396      SGSDDATCRLYDLRADREVAIYSKESIIFGASSVDFSLSGRLLFAGYNDYTINVWDVLKGSRVSILFGHENRVSTLRVSPDGTAFCSGSWDHTLRIWA  395
Gb5L_Xenopus_XP-012814636        SGSDDASCRLYDLRADREVAIYSKESIIFGASSVDFSLSGRLLFAGYNDYTINVWDVLKGTRVSILFGHENRVSTLRVSPDGTAFCSGSWDHTLRIWA  395
Gb5L_Nanorana_XP-018431551       SGSDDASCRLYDLRADREVAIYSKESIIFGASSVDFSLSGRLLFAGYNDYTINVWDVLKGSRVSILFGHENRVSTLRVSPDGTAFCSGSWDHTLRIWA  395
Gb5L_Coelacanth_XP-014351988     SGSDDATCRLYDLRADREVAIYSKESIIFGASSVDFSLSGRLLFAGYNDYTINVWDVLKGTRVSILFGHENRVSTLRVSPDGTAFCSGSWDHTLRIWA  395
Gb5L_Florida_gar_16504-1-1       SGSDDATCRLYDLRADREVAIYSKESIIFGASSVDFSLSGRLLFAGYNDYTINVWDVLKGTRVSILFGHENRVSTLRVSPDGTAFCSGSWDHTLRIWA  395
Gb5L_Bowfin_22627-1-1            SGSDDATCRLYDLRADREVAIYSKESIIFGASSVDFSLSGRLLFAGYNDYTINVWDVLKGTRVSILFGHENRVSTLRVSPDGTAFCSGSWDHTLRIWA  395
Gb5L_Zebrafish_NP-957040         SGSDDATCRLYDLRADREVAIYSKDSIIFGASSVDFSLSGRLLFAGYNDYNINVWDVLKGTRVAILFGHENRVSTVRVSPDGTAFCSGSWDNTLRIWA  395
Gb5L_Medaka_XP-011491247         SASDDATCRFYDLRADREVAVYQKDSVIFGASSVDFSLSGRLLFAGYNDYTINVWDVLKGTRVSILFGHENRVSRVRVSPDGTALCSASWDNTLRIWA  395
Gb5L_Reef_shark_40872-1-1        SGSDDATCRLYDLRADREVAIYSKDSIIFGASSVDFSLSGRLLFAGYNDYTINVWDVLKGTRVSILFGHENRVSTLRVSPDGTAFCSGSWDHTLRIWA  395
Gb5L_Bamboo_shark_37645-2-1      SGSDDATCRLYDLRADREVAIYSKDSIIFGASSVDFSLSGRLLFAGYNDYTINVWDVLKGTRVSILFGHENRVSTLRVSPDGTAFCSGSWDHTLRIWA  395
Gb5L_Bluespot_ray_28709-1-1      SGSDDATCRLYDLRADREVAIYSKDSIIFGASSVDFSLSGRLLFAGYNDYTINVWDVLKGTRVSILFGHENRVSTLRVSPDGTAFCSGSWDHTLRIWA  395
Gb5L_Western_ray_37263-2-2       SGSDDATCRLYDLRADREVAIYSKDSIIFGASSVDFSLSGRLLFAGYNDYTINVWDVLKGTRVSILFGHENRVSTLRVSPDGTAFSSGSWDHTLRIWA  395
Gb5S_Elephant_shark_XP-007904318 SGSDDATCRLYDLRADREVAVYSKDSIIFGASSVDFSLSGRLLFSGYNDYTINVWDVLKGTRVSILFGHENRVSTLRVSPDGTAFCSGSWDHTLRIWA  353
Gb5L_M.mordax_41947-2-2          TGSDDATCRLYDLRADREVAVFSKESIIFGASSVDFSLSGRLLFSGYNDYTINVWDVLKGSRVSILFGHENRVSTLRVSPDGTAFCTGSWDHTLRVWA  395
Gb5L_G.australis_57438-2-2       TGSDDATCRLYDLRADREVAVFSKESIIFGASSVDFSLSGRLLFSGYNDYTINVWDVLKGSRVSILFGHENRVSTLRVSPDGTAFCTGSWDHTLRVWA  395
Gb5L-Partial_P.marinus_S4RZE3    ----------------------------------------------------------------------------------------------------  213
Gb5L_E.cirrhatus_130391-1-2      TGSDDATCRLYDLRADREVAIYSKESIIFGASSVDFSLSGRLLFAGYNDYTINVWDVLKGTRVAILFGHENRVSCLRVSPDGTAFCSASWDHTLKIWA  395
Gb5_S.kowalevskii_XP-002740524   TASDDATCRLYDLRADREVNIYTKESILFGATCLDFSLSGRLLFVGYNDYTVNIWDTLKGIRLAILYGHENRVSCLSVSPDGTSLCTGSWDYTLRVWA  352
Gb5_S.purpuratus_XP-792481       TASDDASCRMYDLRADREISCYSKESIIFGATSLDFSLSGRLLFVGYNDYSVNIWDTLKGHRLTMLYGHENRVSCLRVSPDGTAMCTGSWDYTLRIWA  354
                                 .......310.......320.......330.......340.......350.......360.......370.......380.......390........
```

```
                                                                                                                                                                :            *:   .
R9AP_Human_NP-997274                     M------------------------------------AREE-----------------------------------------------------CKALLDGLNKTTACYHHLVLTVGGSADSQNLRQELQ  41
R9AP_Cattle_NP-001159988                 M------------------------------------AREE-----------------------------------------------------CKALLDALNKATACYHHLVLTIGGSADSQNLREELQ  41
R9AP_Degu_XP-004636749                   M------------------------------------AREE-----------------------------------------------------SKALLDAFNKTTACYHHLVLTVGGSADSQNLREELQ  41
R9AP_Opossum_XP-007474849                M------------------------------------AKEE-----------------------------------------------------CKALLDALNRVTACYRHMVLSVGGTSDSQNLREELK  41
R9AP_Tasmanian_devil_XP-003757239        M------------------------------------AKEE-----------------------------------------------------CKVLLDALNRVTACYRHMVLSVGGTADSQNLREELK  41
R9AP_Koala_XP-020840579                  M------------------------------------AKEE-----------------------------------------------------CKALLDALSRVTACYRHMVLSVGGTADSQNLREELK  41
R9AP_Ground-tit_XP-014110984             M------------------------------------VKEE-----------------------------------------------------CKALLDALNKVTACYRHMVLTIGGTSDSQNLREELK  41
R9AP_Quail_XP-015729517                  M------------------------------------VKEE-----------------------------------------------------CKALLDALSRVTACYRHMVLTIGGTSDSQNLREELK  41
R9AP_Golden_eagle_XP-011572427           M------------------------------------VKEE-----------------------------------------------------CKALLDALNKVTACYRHMVLTIGGTSDSQNLREELK  41
R9AP_Turtle_XP-007058713                 M------------------------------------VKEE-----------------------------------------------------CKALLDALSKVTACYRHMVLTIGGTSDSQNLREELK  41
R9AP_Alligator_XP-014451063              M------------------------------------VKEE-----------------------------------------------------CKALLDALNKVAACYRHMVLTIGGTADSQNLREELR  41
R9AP_Garter_snake_XP-013923971           M------------------------------------AKEE-----------------------------------------------------CKALLDALSKVTACYRHLVLTVGGAADSQELREELK  41
R9AP_Gecko_XP-015270350                  M------------------------------------VKEE-----------------------------------------------------CKALLDALNKVTACYRQLVLTIGGTSDSQNLREELK  41
R9AP_Anole_XP-016852891                  M------------------------------------AKEE-----------------------------------------------------CKALLDALSKVTACYRHLVLTIGGASDSQNLREELQ  41
R9AP_Xenopus_XP-004913611                M------------------------------------LKEE-----------------------------------------------------CKALLDALNKVTACYRHLVLTIGGTSDSQNLREELK  41
R9AP_Nanorana_XP-018425514               M------------------------------------PAEE-----------------------------------------------------CRALLDALNKVTACYRHLVLTIGGTSDSQNLREELK  41
R9AP_Coelacanth_XP-006005212             M------------------------------------VKDE-----------------------------------------------------CKTLLDALNKVTACYRHMVIGVGGTSDSQNLREELK  41
R9AP_Florida_gar_28835-2-1               M------------------------------------GKEE-----------------------------------------------------CKTLLDALNKVTACYRHLVVAVGGTSDSQNLREELK  41
R9AP_Bowfin_1668-1-1                     M------------------------------------GKEE-----------------------------------------------------CKTMLDALNKVTACYRHLVIAVGGTSDSQNLREELK  41
R9AP_Zebrafish_XP-005169975              M------------------------------------GKDE-----------------------------------------------------CKTMLDALNKVTACYRHLVTALGSTSDSQNLREELK  41
R9AP_Asian_bonytongue_XP-018594265       M------------------------------------GKEE-----------------------------------------------------CKTMLDALNRVTACYRHLVITLGSTSDSQNLREELR  41
R9AP_Reef_shark_40575-1-1                -------------------------------------MKDE-----------------------------------------------------CKALLDALNKVSACYRHLVICAGGTSDSQNLREELK  40
R9AP_Bamboo_shark_37642-1-1              M------------------------------------VKDE-----------------------------------------------------CKTLLDALNKVSACYRHLVICAGGSSDSQNLREELK  41
R9AP_Bluespot_ray_25945-2-2              M------------------------------------VNDE-----------------------------------------------------CKTLLDALNKVSACYRHLVICAGGTSDSQNLREELK  41
R9AP_Western_ray_30726-1-1               M------------------------------------VKDE-----------------------------------------------------CKTLLDALNKVSACYRHLVICAGGTSDSQNLREELK  41
R9AP_Elephant_shark_XP-007887765         M------------------------------------VRDE-----------------------------------------------------CKTLLDALNKVSACYRHLVVCVGGTSDSQNLREELR  41
R9AP_M.mordax_41679-5-1                  MA-----------------------------GASSAETDAQ-----------------------------------------------------CAALVQSLNKVTACHHHLAVSLGGSADCARLREELR  48
R9AP_G.australis_56404-2-1               M----------------------------------AETDAQ-----------------------------------------------------CAALVQSLNKVTACHHHLAVSLGGSADCARLRDELR  43
R9AP_L.camtschaticum_JL14914             MA-----------------------------TEGAAESDSQ-----------------------------------------------------CAALVQSLNKVTACHHHLAVSLGGSADCARLHEELR  48
R9AP_E.cirrhatus_138946-5-1              MP-----------------------------GSVMA-ACEE-----------------------------------------------------CRALVQALNKVTACFRQLVMSIGGSSDSLNLREELR  47
R9AP-B_Xenopus_XP-002941417              MP-------------------LQNVKVADEGSVNFQKVKEE-----------------------------------------------------CITAVESLHKVVACYRHLALTIGGSSDSIHLRDELR  58
R9AP-B_Nanorana_XP-018419161             MP-------------------IQNNKVGNEACINSLKVKEE-----------------------------------------------------CSTVVEALNKVVACYRHLAVSVGSSSDSKRLRDELR  58
R9AP-B-Partial_Coelacanth_XP-006013654   MP-------------------IQNIKVASEGSVNSQKTKEE-----------------------------------------------------CRALVEALNKVIACYRHLATCVGSSSDCCNLRDELR  58
R9AP-B_Florida_gar_25574-1-1             MP-------------------VSNNKIANDGNADSIKSREE-----------------------------------------------------AKALVEALIKVVACYRHLAASVGGSTDSSNLRDELR  58
R9AP-B_Zebrafish_NP-001314844            MP-------------------LSNNKVADDGS--STQPLQE-----------------------------------------------------GKAMVDSLIKVVACYRHLASCVGGCTDSSSLRRDLR  56
R9AP-B_Asian_bonytongue_XP-018613071     MT-------------------LCNSKVADRGGDGSQQPLEE-----------------------------------------------------AKTLVDTLAKVVACYRQLSSCVGGSSDSVRLRGELR  58
R9AP-B_Reef_shark_612-1-1                MP-------------------IQNSKVADEGTANSAKARDD-----------------------------------------------------CRSLVDSLNKVTGCYKHLAISVGGSSDCTNLRDELR  58
R9AP-B_Bamboo_shark_45543-9-1            MP-------------------IQSSKVADEGTANSARERDE-----------------------------------------------------CRALVDSLNKVTGCYRHLATGVGGSADCSNLRDELR  58
R9AP-B_Bluespot_ray_37118-18-1           MP-------------------IQNIKVADEGTVGSAKARDD-----------------------------------------------------CRALVDSLNKVTGCYRHLAIGIGGSADSAGLRDELR  58
R9AP-B_Western_ray_39739-1-1             MP-------------------IQNIKVADEGAAGSAKARDD-----------------------------------------------------CRALVDSLNKVTGCYRHLAIGVGGSADSVGLRDELR  58
R9AP-B_Elephant_shark_XP-007902235       MP-------------------IHSTKVADEGTAKSERARGE-----------------------------------------------------CKALVQSLNKVIGCYKHLAVNLGGSSDSTNLRDELR  58
R9AP-like_Degu_XP-004636181              MQ---------------------RQ-PG-PREPE-DAATAE-----------------------------------------------------SRAALDALCRFAAGYRHLAMGVGSSADGPRLRLALE  53
R9AP-like_Opossum_XP-007475029           MR---------------------GGGAG-ERAREAREAVAE-----------------------------------------------------CRAAYEALNRFTASYRQLVLGVGSSADGRRLRRALE  55
R9AP-like_Tasmanian_devil_XP-003759057   MR---------------------GAGPG-ERAREAREAVAE-----------------------------------------------------CRAAYEALNRFTASYRQLVLGVGSSADGRRLRRALE  55
R9AP-like_Koala_XP-020821390             MR---------------------GAGPG-ERAREAREAVAE-----------------------------------------------------CRAAYEALNRFTASYRQLVLGVGSSADGRRLRRALE  55
R9AP-like_Quail_XP-015737481             MA------------------------PG-RGAASGCGARGV-----------------------------------------------------CVATQAALCKAAAGHRQLVLQLGGSADGPQLREERR  52
R9AP-like_Ground-tit_XP-005524972        MA------------------------PGRRDACRGRGAVGT-----------------------------------------------------CATAQVALCKATAGHRQLVLQLGGSSDSPQLREERR  53
R9AP-like_Turtle_XP-007067829            MS---------------------HA----PDSHSVGEVRGA-----------------------------------------------------CAATHTSLSKVIVWHNQLALQLGGTSDSPQLRDELQ  52
R9AP-like_Alligator_XP-014450013         MA--------------------------------SRAPVGE-----------------------------------------------------CAAAHAALGRVTARHRQLALLLGSAADCAQLREELQ  45
R9AP-like_Coelacanth_XP-005986755        MP--------------------LLRSRSVDDAQNIGELVKE-----------------------------------------------------TKKAQALLSKILSCFRHLVLCIGGTSDSAKLREELE  57
R9AP-like_Xenopus_NP-001011277           MAAPEVSSAGPNMLHPNSRTLTLRGKRGHAAEDSSQGAVSD-----------------------------------------------------CKKMHSSLNKVTACYRQLVLCVGGTSDCTRLREELE  77
R9AP-like_Nanorana_XP-018419805          MSAPEVASPAPPHLHP-SRTLTLRGIRGHATESSGQGTVSE-----------------------------------------------------CKKVHISLNKVTACYRQLVLCVGGTSDCTRLREELE  76
R9AP-like_Spotted_gar_XP-006639786       MS---------------------PWRRSVGELPAAQGSVAE-----------------------------------------------------CKKAQASLTRVTACFQQLAMCVGGSTDCSRLREELE  56
R9AP-like_Zebrafish_NP-001018364         MN---------------------RWQRSVVEIQTRKRQVNE-----------------------------------------------------CERAQVALSKVTACFQQMSTSLGSNVDGSFLREEME  56
R9AP-like_Asian_bonytongue_XP-018611369  MN---------------------RWRKSVGEIQARRRAVAE-----------------------------------------------------CTRAQVAFSRVTACFQQLATCVGSSSDCSRLREELE  56
R9AP_S.kowalevskii_XP-006814063          MH-------------------LTIFPE---------------------------------------------------------------------RMLVREFNSATAYYRNLIITLGGSHDSESLRDEIR  43
R9AP_S.purpuratus_XP-011683108           MG-------------------TWIFLLGLLGRGTPLQKRASEFVKPSSAIHSSGMNALHFGKNMIRRHNSCNRNHSDIQSDPETDEIEDCGMPLCTKLVKDFNVETARYHDLTLTLGGSSDSEYLRDELK  111
R9AP_S.purpuratus_XP-011666321           ---------------------------------------------------------------------------------------------------------------MKKTLHVPDISEYLRDELK  19
                                         1.......10........20........30........40........50........60........70........80........90.......100.......110.......120.......130
```

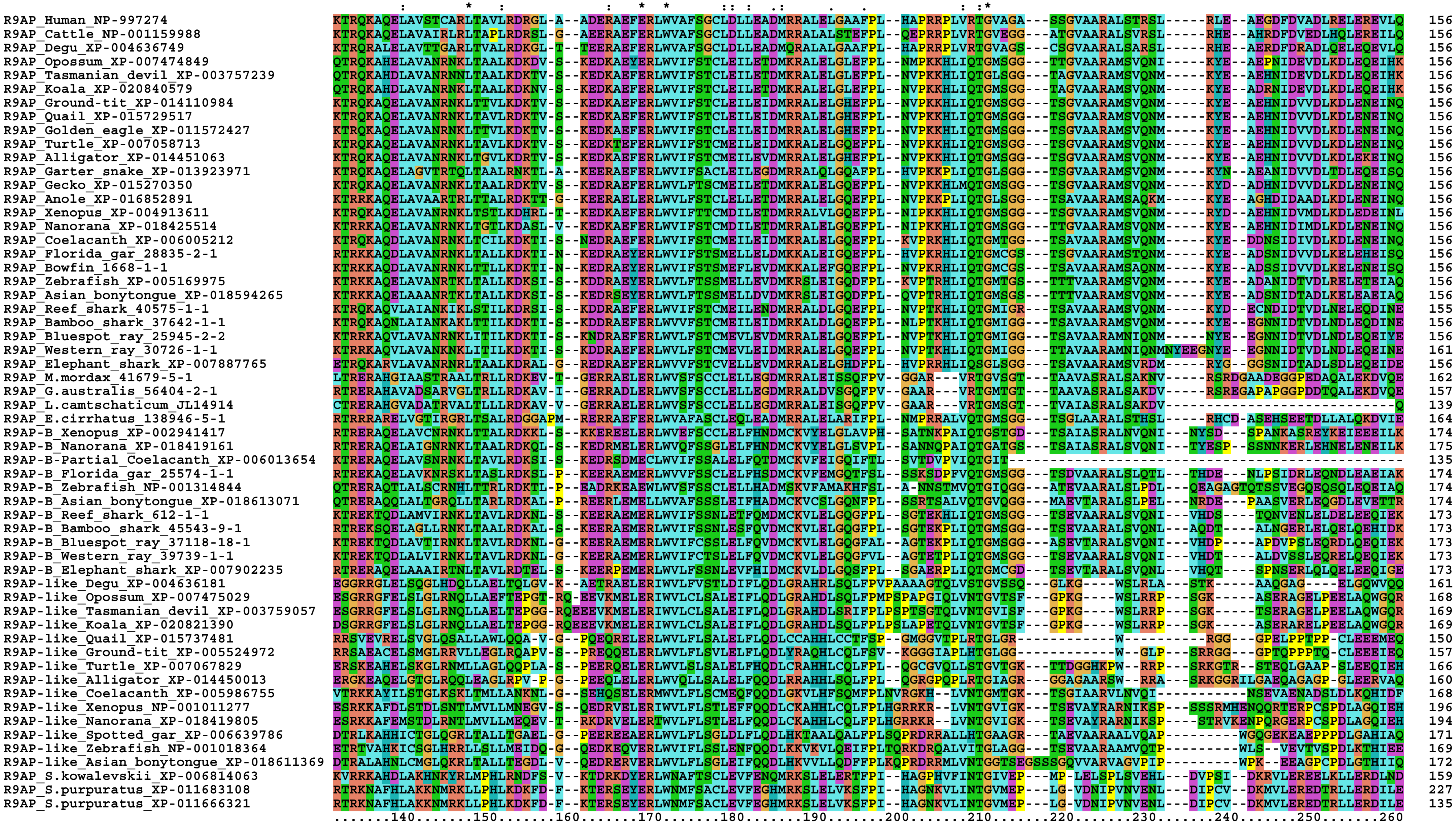

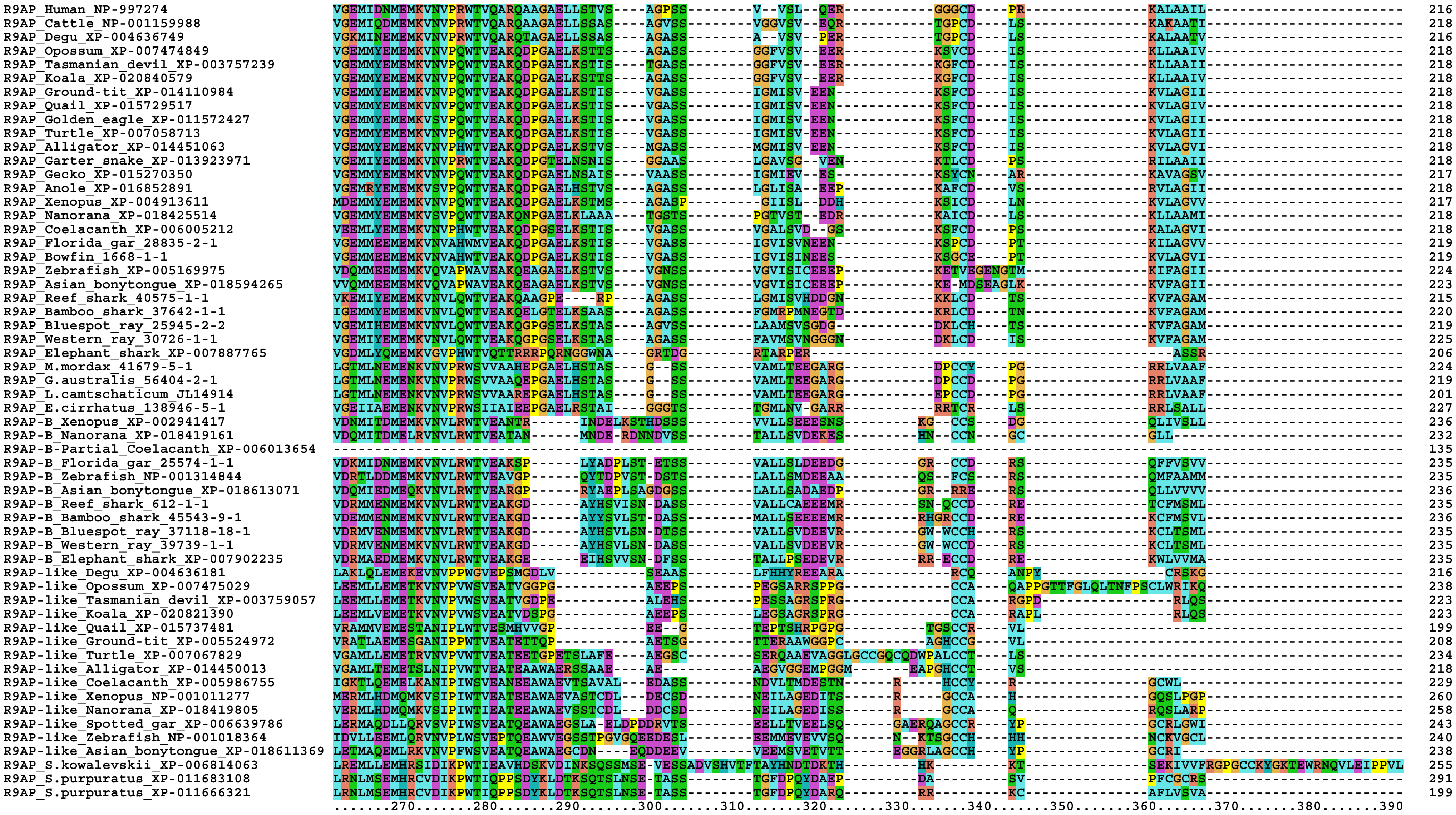

R9AP_Human_NP-997274 216
R9AP_Cattle_NP-001159988 218
R9AP_Degu_XP-004636749 216
R9AP_Opossum_XP-007474849 218
R9AP_Tasmanian_devil_XP-003757239 218
R9AP_Koala_XP-020840579 218
R9AP_Ground-tit_XP-014110984 218
R9AP_Quail_XP-015729517 218
R9AP_Golden_eagle_XP-011572427 218
R9AP_Turtle_XP-007058713 218
R9AP_Alligator_XP-014451063 218
R9AP_Garter_snake_XP-013923971 218
R9AP_Gecko_XP-015270350 217
R9AP_Anole_XP-016852891 218
R9AP_Xenopus_XP-004913611 217
R9AP_Nanorana_XP-018425514 218
R9AP_Coelacanth_XP-006005212 218
R9AP_Florida_gar_28835-2-1 219
R9AP_Bowfin_1668-1-1 219
R9AP_Zebrafish_XP-005169975 224
R9AP_Asian_bonytongue_XP-018594265 223
R9AP_Reef_shark_40575-1-1 215
R9AP_Bamboo_shark_37642-1-1 220
R9AP_Bluespot_ray_25945-2-2 219
R9AP_Western_ray_30726-1-1 225
R9AP_Elephant_shark_XP-007887765 206
R9AP_M.mordax_41679-5-1 224
R9AP_G.australis_56404-2-1 219
R9AP_L.camtschaticum_JL14914 201
R9AP_E.cirrhatus_138946-5-1 227
R9AP-B_Xenopus_XP-002941417 236
R9AP-B_Nanorana_XP-018419161 232
R9AP-B-Partial_Coelacanth_XP-006013654 135
R9AP-B_Florida_gar_25574-1-1 235
R9AP-B_Zebrafish_NP-001314844 235
R9AP-B_Asian_bonytongue_XP-018613071 236
R9AP-B_Reef_shark_612-1-1 235
R9AP-B_Bamboo_shark_45543-9-1 236
R9AP-B_Bluespot_ray_37118-18-1 235
R9AP-B_Western_ray_39739-1-1 235
R9AP-B_Elephant_shark_XP-007902235 235
R9AP-like_Degu_XP-004636181 216
R9AP-like_Opossum_XP-007475029 238
R9AP-like_Tasmanian_devil_XP-003759057 223
R9AP-like_Koala_XP-020821390 223
R9AP-like_Quail_XP-015737481 199
R9AP-like_Ground-tit_XP-005524972 208
R9AP-like_Turtle_XP-007067829 234
R9AP-like_Alligator_XP-014450013 218
R9AP-like_Coelacanth_XP-005986755 229
R9AP-like_Xenopus_NP-001011277 260
R9AP-like_Nanorana_XP-018419805 258
R9AP-like_Spotted_gar_XP-006639786 243
R9AP-like_Zebrafish_NP-001018364 240
R9AP-like_Asian_bonytongue_XP-018611369 238
R9AP_S.kowalevskii_XP-006814063 255
R9AP_S.purpuratus_XP-011683108 291
R9AP_S.purpuratus_XP-011666321 199
.......270.......280.......290.......300.......310.......320.......330.......340.......350.......360.......370.......380.......390

```
R9AP_Human_NP-997274                        -----------FGAVLLAAVALAVCVAKLS---------------------------------------------------------------------   235
R9AP_Cattle_NP-001159988                    -----------FSAVLLAAVALAVCVAKLS---------------------------------------------------------------------   237
R9AP_Degu_XP-004636749                      -----------FSAVLLAAVALALCVAKLS---------------------------------------------------------------------   235
R9AP_Opossum_XP-007474849                   -----------FSAVLIIAVILAVCVVKLS---------------------------------------------------------------------   237
R9AP_Tasmanian_devil_XP-003757239           -----------FSAVLIIAVILAVCVVKLS---------------------------------------------------------------------   237
R9AP_Koala_XP-020840579                     -----------FSAVLIIAVILAVCVVKLS---------------------------------------------------------------------   237
R9AP_Ground-tit_XP-014110984                -----------FTAVLLIAIVLAVCVVKLS---------------------------------------------------------------------   237
R9AP_Quail_XP-015729517                     -----------FSAVLIIAIVLAVCVVKLS---------------------------------------------------------------------   237
R9AP_Golden_eagle_XP-011572427              -----------FSAVLIIAIVLAVCVVKLS---------------------------------------------------------------------   237
R9AP_Turtle_XP-007058713                    -----------FSAVLIIAIVLAVCVVKLS---------------------------------------------------------------------   237
R9AP_Alligator_XP-014451063                 -----------FSAVLIIAIVLAVCVVKLS---------------------------------------------------------------------   237
R9AP_Garter_snake_XP-013923971              -----------FTAVLLIAIGLAVCVAKLS---------------------------------------------------------------------   237
R9AP_Gecko_XP-015270350                     -----------FSAVLLIAVVLAVCVVKLS---------------------------------------------------------------------   236
R9AP_Anole_XP-016852891                     -----------FTAVLLVAIILAVCVAKLS---------------------------------------------------------------------   237
R9AP_Xenopus_XP-004913611                   -----------FSAVLIIAIILAVCVVKLS---------------------------------------------------------------------   236
R9AP_Nanorana_XP-018425514                  -----------FSAVLLVAVILAVCVVKLS---------------------------------------------------------------------   237
R9AP_Coelacanth_XP-006005212                -----------FTAVLMIAIVLAVCVVKLS---------------------------------------------------------------------   237
R9AP_Florida_gar_28835-2-1                  -----------FTAVLFVAIVLALCVIKLS---------------------------------------------------------------------   238
R9AP_Bowfin_1668-1-1                        -----------FTAVLLIAIILAFCVIKLS---------------------------------------------------------------------   238
R9AP_Zebrafish_XP-005169975                 -----------FTAVLLIAGILGYLVINL----------------------------------------------------------------------   242
R9AP_Asian_bonytongue_XP-018594265          -----------FTAIILMAGILAFCVIKLS---------------------------------------------------------------------   242
R9AP_Reef_shark_40575-1-1                   -----------FAAVLIFAIALAVCVVKFA---------------------------------------------------------------------   234
R9AP_Bamboo_shark_37642-1-1                 -----------FTAVLVFAIVLALCVVKFA---------------------------------------------------------------------   239
R9AP_Bluespot_ray_25945-2-2                 -----------FSAVLIIAIVLAFCIVKFA---------------------------------------------------------------------   238
R9AP_Western_ray_30726-1-1                  -----------FSAVLIIAIVLAACIVKFA---------------------------------------------------------------------   244
R9AP_Elephant_shark_XP-007887765            -----------FP-------TRAPTRSRTA---------------------------------------------------------------------   218
R9AP_M.mordax_41679-5-1                     -----------CGGALLLALVLAAAVVIFS---------------------------------------------------------------------   243
R9AP_G.australis_56404-2-1                  -----------CGGALLLAFVLAAAVVMFA---------------------------------------------------------------------   238
R9AP_L.camtschaticum_JL14914                -----------CGGALLLALVLAAAVVMFA---------------------------------------------------------------------   220
R9AP_E.cirrhatus_138946-5-1                 -----------FTMIILLAVVLSVCVAKFA---------------------------------------------------------------------   246
R9AP-B_Xenopus_XP-002941417                 -----------FCGTALVAITLYS----IL---------------------------------------------------------------------   251
R9AP-B_Nanorana_XP-018419161                ----------------------------FL---------------------------------------------------------------------   234
R9AP-B-Partial_Coelacanth_XP-006013654      ---------------------------------------------------------------------------------------------------   135
R9AP-B_Florida_gar_25574-1-1                -----------LCGIAMVAVVLSVCVVYLT---------------------------------------------------------------------   254
R9AP-B_Zebrafish_NP-001314844               -----------LCGVAMFAVVLSVCVVFLV---------------------------------------------------------------------   254
R9AP-B_Asian_bonytongue_XP-018613071        -----------VSVAVAVATMLFVCIVYLV---------------------------------------------------------------------   255
R9AP-B_Reef_shark_612-1-1                   -----------FCTVALIAVVLSVCVVNLA---------------------------------------------------------------------   254
R9AP-B_Bamboo_shark_45543-9-1               -----------FCTVALIAVGLSVCVVNLS---------------------------------------------------------------------   255
R9AP-B_Bluespot_ray_37118-18-1              -----------LCTAALAAVVLSVCAANIA---------------------------------------------------------------------   254
R9AP-B_Western_ray_39739-1-1                -----------LCTAALTAVVLSVCAANLA---------------------------------------------------------------------   254
R9AP-B_Elephant_shark_XP-007902235          -----------LCTAALVAVVLSVCVATFA---------------------------------------------------------------------   254
R9AP-like_Degu_XP-004636181                 ---------------------------------------------------------------------------------------------------   216
R9AP-like_Opossum_XP-007475029              -----------HKNSF-----------------------------------------------------------------------------------   243
R9AP-like_Tasmanian_devil_XP-003759057      -----------HCIVA-----------------------------------------------------------------------------------   228
R9AP-like_Koala_XP-020821390                -----------HCIVA-----------------------------------------------------------------------------------   228
R9AP-like_Quail_XP-015737481                ---------------------------------------------------------------------------------------------------   199
R9AP-like_Ground-tit_XP-005524972           ---------------------------------------------------------------------------------------------------   208
R9AP-like_Turtle_XP-007067829               ---------------------------------------------------------------------------------------------------   234
R9AP-like_Alligator_XP-014450013            ---------------------------------------------------------------------------------------------------   218
R9AP-like_Coelacanth_XP-005986755           -----------ICVIS-----------------------------------------------------------------------------------   234
R9AP-like_Xenopus_NP-001011277              -----------LCMVS-----------------------------------------------------------------------------------   265
R9AP-like_Nanorana_XP-018419805             -----------LCMVS-----------------------------------------------------------------------------------   263
R9AP-like_Spotted_gar_XP-006639786          -----------LCLVN-----------------------------------------------------------------------------------   248
R9AP-like_Zebrafish_NP-001018364            -----------LCLLS-----------------------------------------------------------------------------------   245
R9AP-like_Asian_bonytongue_XP-018611369     -----------LCMLH-----------------------------------------------------------------------------------   243
R9AP_S.kowalevskii_XP-006814063             LSGLRGAHFIDIKYLVEFKAVVPLAPFDLQVKLPITIGSVPLQQIHAQLDQRAASLGPGCATPELSAQQDDVPVPNIPPPTYEQVCGGGQVNDAEDNKL   354
R9AP_S.purpuratus_XP-011683108              -----------V---------------------------------------------------------------------------------------   292
R9AP_S.purpuratus_XP-011666321              -----------LITLVAFAAVLIICLVLLEGK-------------------------------------------------------------------   220
                                            .......400.......410.......420.......430.......440.......450.......460.......470.......480.........
```
